# Supplementary material for: Synthesis and Evaluation of Biological Activities of Bis(spiropyrazolone)cyclopropanes: A Potential Application against Leishmaniasis
Source: Molecules. 2021 Aug 17;26(16):4960. doi: 10.3390/molecules26164960 (PMC8398714; doi:10.3390/molecules26164960)
Supplement: Supplementary file 1 [file molecules-26-04960-s001.zip › molecules-1302950-supplementary.pdf]

Supporting Information

Synthesis and evaluation of biological activities of Bis(spiropyrazolone)cyclopropanes: a potential application against leishmaniasis.

Olalla Barreiro-Costa[1,†], Gabriela Morales-Noboa[2,†], Patricio Rojas-Silva[1], Eliana Lara-Barba[2], Javier Santamaría-Aguirre[2], Natalia Bailón-Moscoso[3], Juan Carlos Romero-Benavides[4], Ana Herrera[3], Cristina Cueva[3], Lenin Ron-Garrido[2], Ana Poveda[2,*] and Jorge Heredia-Moya[1,*]

[1] Centro de Investigación Biomédica (CENBIO), Facultad de Ciencias de la Salud Eugenio Espejo, Universidad UTE, Quito 170527, Ecuador; olallabarreirocosta@gmail.com, (O.B.-C.) projas1@usfq.edu.ec (P.R.-S.), jorgeh.heredia@ute.edu.ec (J.H.-M.)

[2] DNA replication and Genome Instability Unit, Grupo de Investigación en Biodiversidad, Zoonosis y Salud Pública (GIBCIZ), Instituto de Investigación en Salud Pública y Zoonosis-CIZ, Facultad de Ciencias Químicas, Facultad de Ciencias Agrícolas, Universidad Central del Ecuador, Quito 170521, Ecuador; gemoralesnoboa@hotmail.com, (G.M.-N.) eliluci1@hotmail.com, (E.L.-B.), jrsantamaria@uce.edu.ec, (J. S.-A.), ljron@uce.edu.ec, (L.R.-G.), apoveda@uce.edu.ec (A.P.)

[3] Departamento de Ciencias de la Salud, Universidad Técnica Particular de Loja, Loja 1101608, Ecuador; ncbailon@utpl.edu.ec (N. B.-M.), mccueva5@utpl.edu.ec (C.C.), agherrera5@utpl.edu.ec (A. H.)

[4] Departamento de Química y Ciencias Exactas, Universidad Técnica Particular de Loja, Loja 1101608, Ecuador; jcromerob@utpl.edu.ec (J.C.R.-B.)

* Correspondence: apoveda@uce.edu.ec (A.P.) and jorgeh.heredia@ute.edu.ec (J.H.-M.)

† These authors contributed equally to this work.

Supporting Figures

Figure S1. Cell morphology of RKO, PC-3 and HeLa cell lines exposed to the Inhibitory concentration (IC_{50}) of the most active bis (spiropyrazolone) cyclopropanes for 48 hours. Data are given as mean and standard error (SEM) of at least three independent experiments. Horizontal bar in the figure below = 50 μm. 4

Figure S2. Drop test for *S. cerevisiae* strains in presence of bis(spiropyrazolone)cyclopropanes **4**. Each line of serial-diluted cells dots corresponds with the strain indicated at the left of the panel. The compound evaluated in each panel is indicated below. 4

Figure S3. Stability of bis(spiropyrazolone)cyclopropanes **4**. (A) Absorbance spectra at 480 nm of **4b** (100 μM) in DMSO at different time of incubation at 25 °C. (B) Absorbance spectra at 480 nm of **4b** (100 μM) in PBS 1X pH 7.4 at different time of incubation at 25 °C. (C) Stability of **4b** (2300 μM) in DMSO and PBS 1X pH 7.4 at different time of incubation at 25 °C. (D) Inhibitory activity of products of thermal isomerization of bis(spiropyrazolone)cyclopropanes **4** against *L. mexicana* promastigotes at 10 μM. 5

Figure S4. Representative examples of *Leishmania mexicana* cells exposed to bis(spiropyrazolone)cyclopropanes **4** and subjected to comet assay. C, control unexposed cells; DMSO, dimethyl sulfoxide; MMS, methyl methanesulfonate. 6

Figure S5. ^{1}H NMR spectrum of compound **2a** 7

Figure S6. ^{1}H NMR spectrum of compound **2b** 7

Figure S7. ^{1}H NMR spectrum of compound **2c** 8

Figure S8. ^{1}H NMR spectrum of compound **2d** 8

Figure S9. ^{1}H NMR spectrum of compound **2e** 9

Figure S10. ^{1}H NMR spectrum of compound **2f** 9

Figure S11. ^{1}H NMR spectrum of compound **2g** 10

Figure S12. ^{1}H NMR spectrum of compound **2h** 10
Figure S13. ^{13}C NMR spectrum of compound **2h** 11
Figure S14. FTIR spectrum of compound **2h** 11
Figure S15. HRMS spectrum of compound **2h** 12
Figure S16. ^{1}H NMR spectrum of compound **2i**. 12
Figure S17. ^{1}H NMR spectrum of compound **2j**. 13
Figure S18. ^{1}H NMR spectrum of compound **2k**. 13
Figure S19. ^{1}H NMR spectrum of compound **2l**. 14
Figure S20. ^{1}H NMR spectrum of compound **2m** 14
Figure S21. ^{13}C NMR spectrum of compound **2m** 15
Figure S22. FTIR spectrum of compound **2m** 15
Figure S23. HRMS spectrum of compound **2m** 16
Figure S24. ^{1}H NMR spectrum of compound **2n**. 16
Figure S25. ^{1}H NMR spectrum of compound **2o**. 17
Figure S26. ^{1}H NMR spectrum of compound **2p**. 17
Figure S27. ^{19}F NMR spectrum of compound **2p**. 18
Figure S28. ^{1}H NMR spectrum of compound **2q**. 18
Figure S29. ^{1}H NMR spectrum of compound **2r**. 19
Figure S30. ^{1}H NMR spectrum of compound **2s**. 19
Figure S31. ^{1}H NMR spectrum of compound **2t**. 20
Figure S32. ^{1}H NMR spectrum of compound **2u**. 20
Figure S33. ^{1}H NMR spectrum of compound **4b** 21
Figure S34. ^{13}C NMR spectrum of compound **4b** 21
Figure S35. FTIR spectrum of compound **4b** 22
Figure S36. ESI-MS spectrum of compound **4b** 22
Figure S37. ^{1}H NMR spectrum of compound **4c** 23
Figure S38. ^{13}C NMR spectrum of compound **4c** 23
Figure S39. FTIR spectrum of compound **4c** 24
Figure S40. ESI-MS spectrum of compound **4c** 24
Figure S41. ^{1}H NMR spectrum of compound **4d** 25
Figure S42. ^{13}C NMR spectrum of compound **4d** 25
Figure S43. FTIR spectrum of compound **4d** 26
Figure S44. ESI-MS spectrum of compound **4d** 26
Figure S45. ^{1}H NMR spectrum of compound **4e** 27
Figure S46. ^{13}C NMR spectrum of compound **4e** 27
Figure S47. FTIR spectrum of compound **4e** 28
Figure S48. ESI-MS spectrum of compound **4e** 28
Figure S49. ^{1}H NMR spectrum of compound **4f** 29
Figure S50. ^{13}C NMR spectrum of compound **4f** 29
Figure S51. ^{1}H NMR spectrum of compound **4k** 30
Figure S52. ^{13}C NMR spectrum of compound **4k** 30
Figure S53. FTIR spectrum of compound **4k** 31
Figure S54. ESI-MS spectrum of compound **4k** 31
Figure S55. ^{1}H NMR spectrum of compound **4p** 32
Figure S56. ^{13}C NMR spectrum of compound **4p** 32
Figure S57. ^{19}F NMR spectrum of compound **4p** 33
Figure S58. FTIR spectrum of compound **4p** 33
Figure S59. ESI-MS spectrum of compound **4p** 34
Figure S60. ^{1}H NMR spectrum of compound **4q** 34
Figure S61. ^{13}C NMR spectrum of compound **4q** 35
Figure S62. FTIR spectrum of compound **4q** 35
Figure S63. ESI-MS spectrum of compound **4q** 36
Figure S64. ^{1}H NMR spectrum of compound **4r** 36

Figure S65. ^{13}C NMR spectrum of compound **4r** 37
Figure S66. ^{19}F NMR spectrum of compound **4r** 37
Figure S67. FTIR spectrum of compound **4r** 38
Figure S68. ESI-MS spectrum of compound **4r** 38
Figure S69. ^{1}H NMR spectrum of compound **4s** 39
Figure S70. ^{13}C NMR spectrum of compound **4s** 39
Figure S71. ^{19}F NMR spectrum of compound **4s** 40
Figure S72. FTIR spectrum of compound **4s** 40
Figure S73. ESI-MS spectrum of compound **4s** 41
Figure S74. ^{1}H NMR spectrum of compound **4t** 41
Figure S75. ^{13}C NMR spectrum of compound **4t** 42
Figure S76. ^{19}F NMR spectrum of compound **4t** 42
Figure S77. FTIR spectrum of compound **4t** 43
Figure S78. ESI-MS spectrum of compound **4t** 43
Figure S79. ^{1}H NMR spectrum of compound **4u** 44
Figure S80. ^{13}C NMR spectrum of compound **4u** 44
Figure S81. FTIR spectrum of compound **4u** 45
Figure S82. ESI-MS spectrum of compound **4u** 45
Figure S83. ^{1}H NMR spectrum of compound **5g** 46
Figure S84. ^{1}H NMR spectrum of compound **6** 46
Figure S85. ^{13}C NMR spectrum of compound **6** 47
Figure S86. DEPT spectrum of compound **6** 47
Figure S87. HSQC spectrum of compound **6** 48
Figure S88. HMBC spectrum of compound **6** 48
Figure S89. FTIR spectrum of compound **6** 49
Figure S90. ESI-MS spectrum of compound **6** 49

Supporting Tables

Table S1. Percentage (%) of inhibition of five human cancer cell lines, and immortalized cell CHO K-1 exposed 48 h to 100 μM of each bis(spiropyrazolone)cyclopropanes **4.** 50
Table S2. Inhibitory activity of bis(spiropyrazolone)cyclopropanes **4** against *S. cerevisiae* (W303 background). 50
Table S3. DPPH scavenging activity of bis(spiropyrazolone)cyclopropanes **4.** 51

Statistical Analyses

Statistical Analyses S1. Test for statistical significance were based on one-way ANOVA test applied to leishmanicidal and cytotoxicity activities of bis(spiropyrazolone)cyclopropanes **4** (Table 3). 55
Statistical Analyses S2. Kruskal Wallis test´s applied to comet assay of *Leishmania mexicana* cells exposed to bis(spiropyrazolone)cyclopropanes **4** (Figure 2). 55
Statistical Analyses S3. Kruskal Wallis test´s applied to comet assay of CHO cells exposed to bis(spiropyrazolone)cyclopropanes **4** (Figure 3). 58

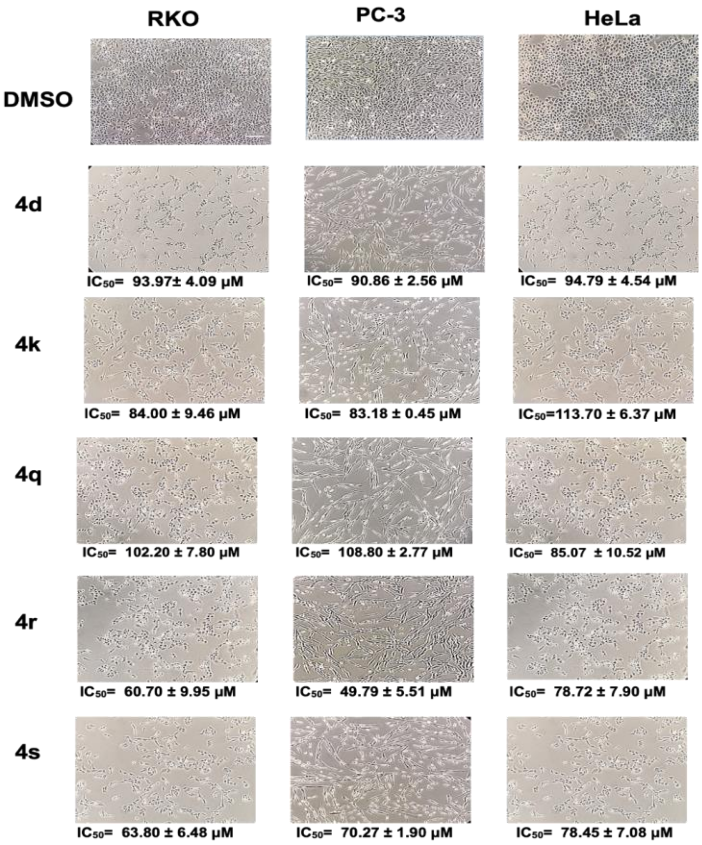

Figure S1. Cell morphology of RKO, PC-3 and HeLa cell lines exposed to the Inhibitory concentration (IC_{50}) of the most active bis (spiropyrazolone) cyclopropanes for 48 hours. Data are given as mean and standard error (SEM) of at least three independent experiments. Horizontal bar in the figure below = 50 µm.

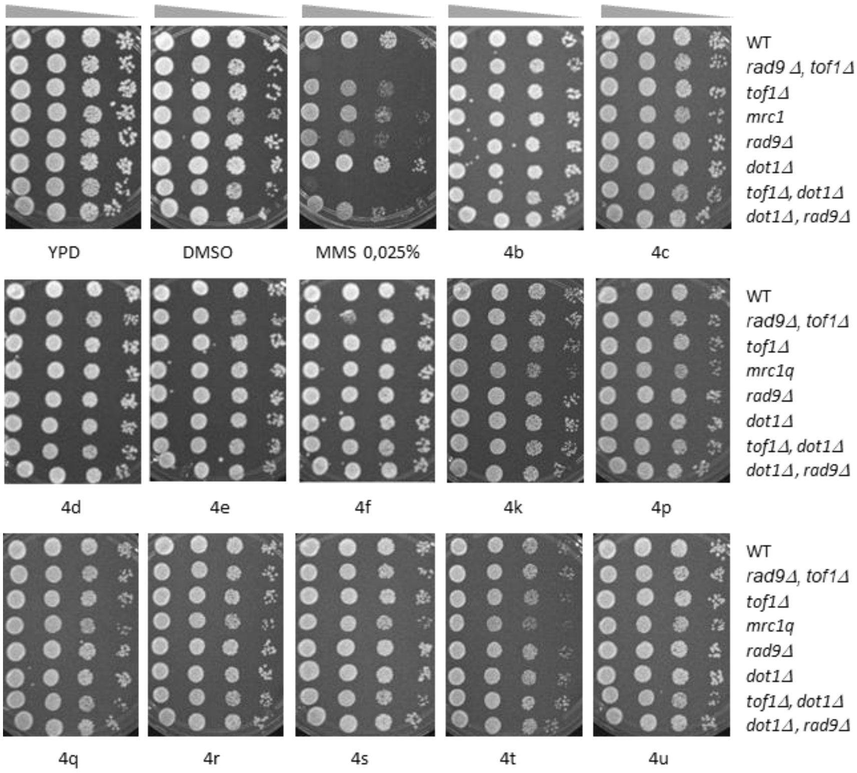

Figure S2. Drop test for *S. cerevisiae* strains in presence of bis(spiropyrazolone)cyclopropanes **4**. Each line of serial-diluted cells dots corresponds with the strain indicated at the left of the panel. The compound evaluated in each panel is indicated below.

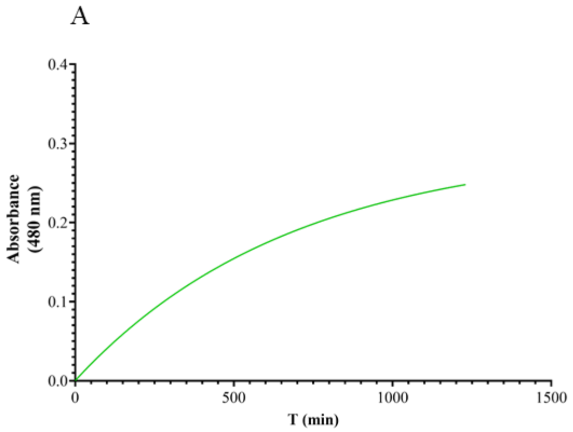

	4b in DMSO
One phase exponential decay	
Best-fit values	
Span	-0.2963
K	0.001472
Plateau	0.2965
HalfLife	471.0
Tau	679.5
95% CI (profile likelihood)	
Span	-0.3016 to -0.2914
K	0.001406 to 0.001538
Plateau	0.2906 to 0.3027
HalfLife	450.8 to 493.0
Tau	650.3 to 711.3
Goodness of Fit	
Degrees of Freedom	39
R squared	0.9991
Sum of Squares	0.0001981
Sy.x	0.002254
Constraints	
K	K > 0
Number of points	

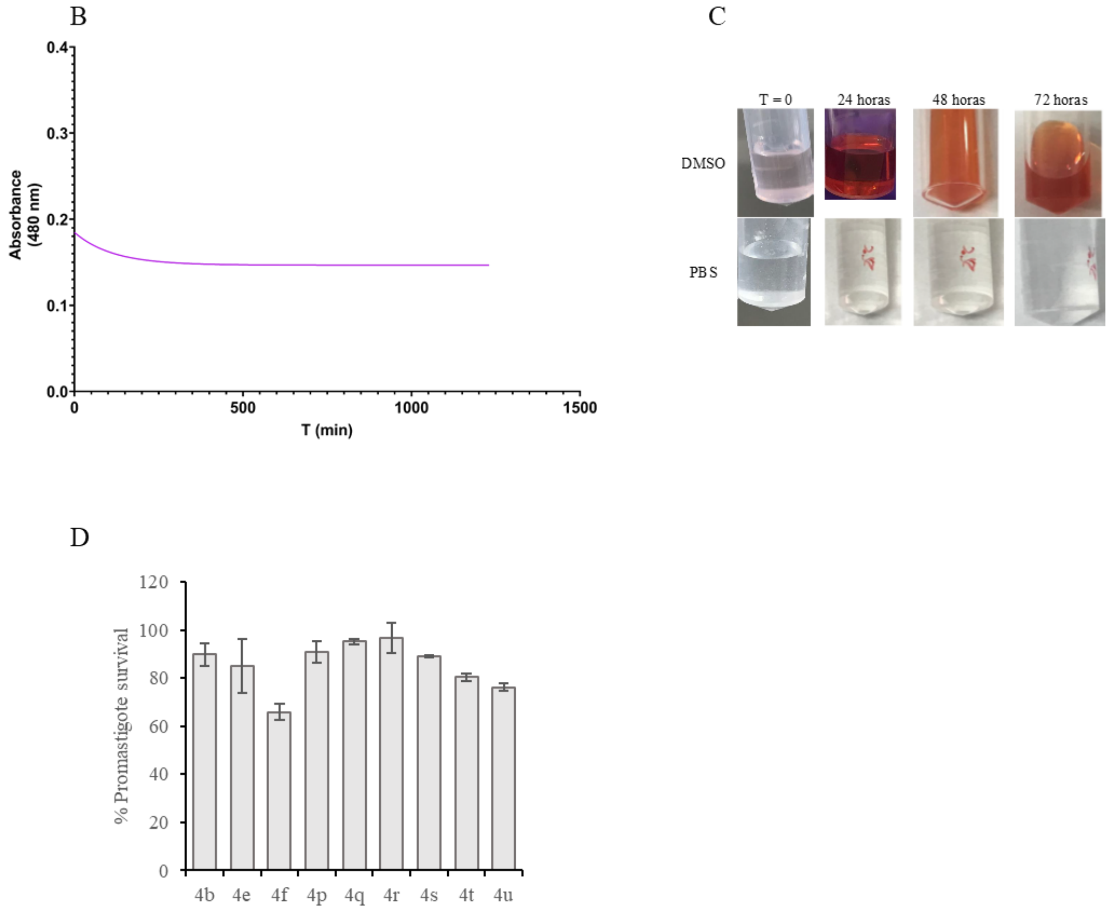

Figure S3. Stability of bis(spiropyrazolone)cyclopropanes **4**. (A) Absorbance spectra at 480 nm of **4b** (100 µM) in DMSO at different time of incubation at 25 °C. (B) Absorbance spectra at 480 nm of **4b** (100 µM) in PBS 1X pH 7.4 at different time of incubation at 25 °C. (C) Stability of **4b** (2300 µM) in DMSO and PBS 1X pH 7.4 at different time of incubation at 25 °C. (D) Inhibitory activity of products of thermal isomerization of bis(spiropyrazolone)cyclopropanes **4** against *L. mexicana* promastigotes at 10 µM.

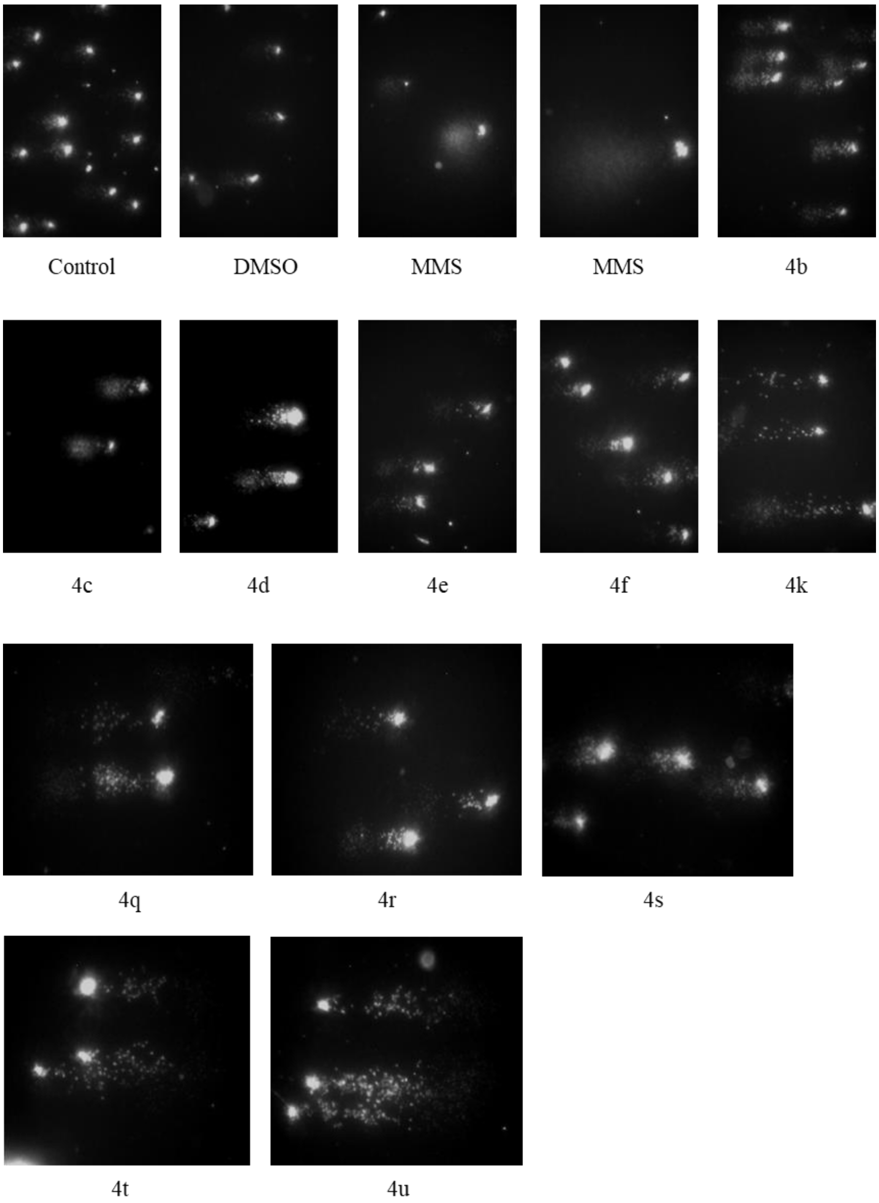

Figure S4. Representative examples of *Leishmania mexicana* cells exposed to bis(spiropyrazolone)cyclopropanes **4** and subjected to comet assay. C, control unexposed cells; DMSO, dimethyl sulfoxide; MMS, methyl methanesulfonate.

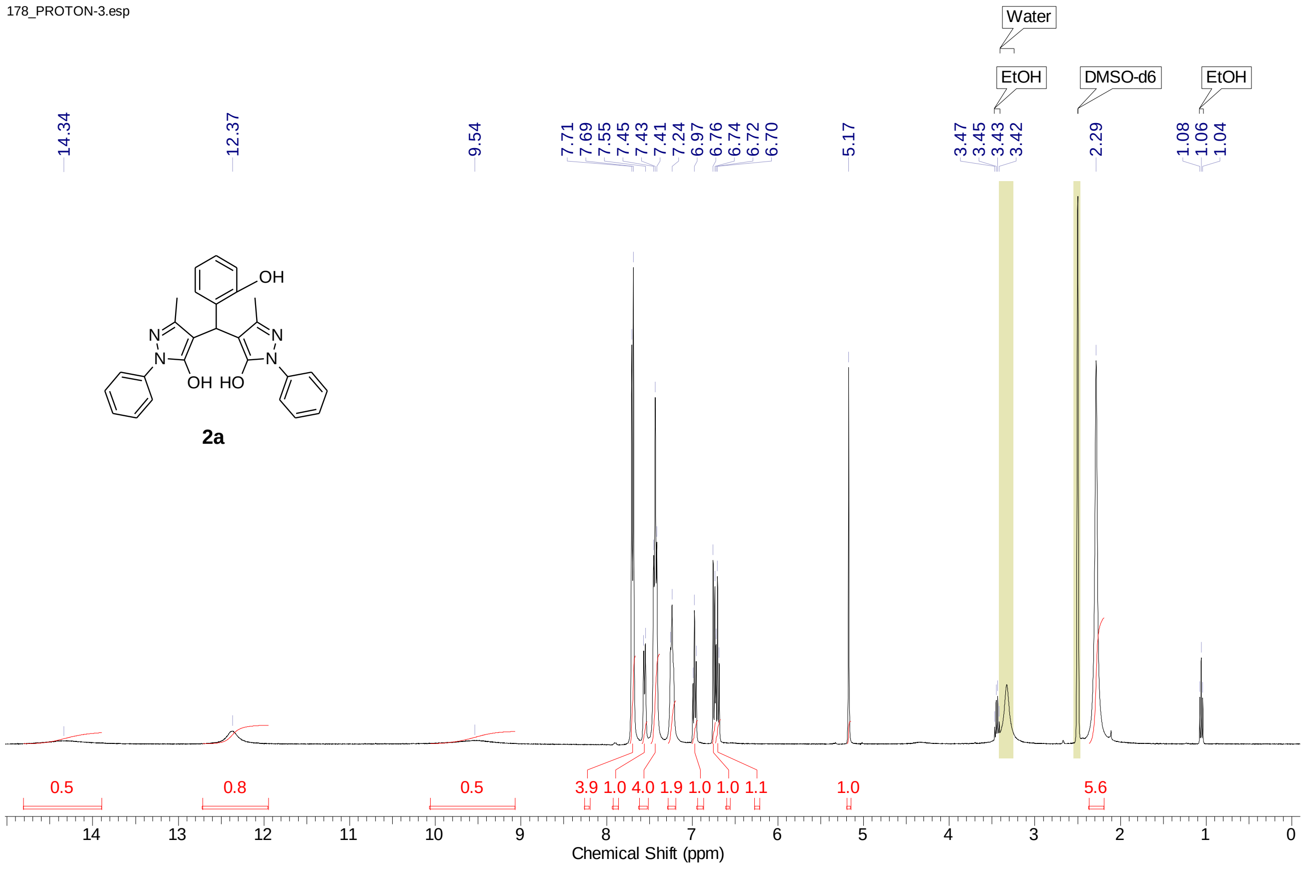

Figure S5. ¹H NMR spectrum of compound **2a**

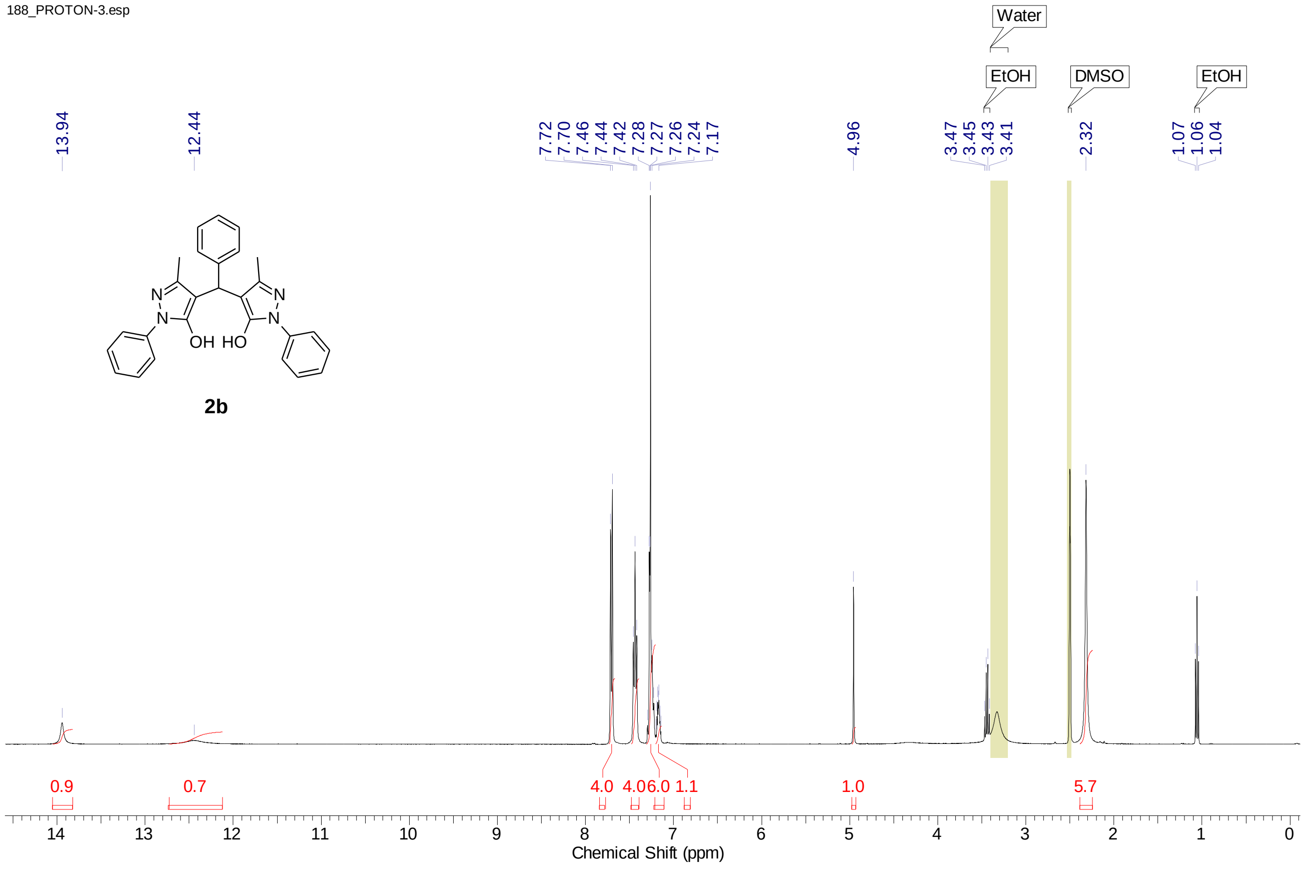

Figure S6. ¹H NMR spectrum of compound **2b**

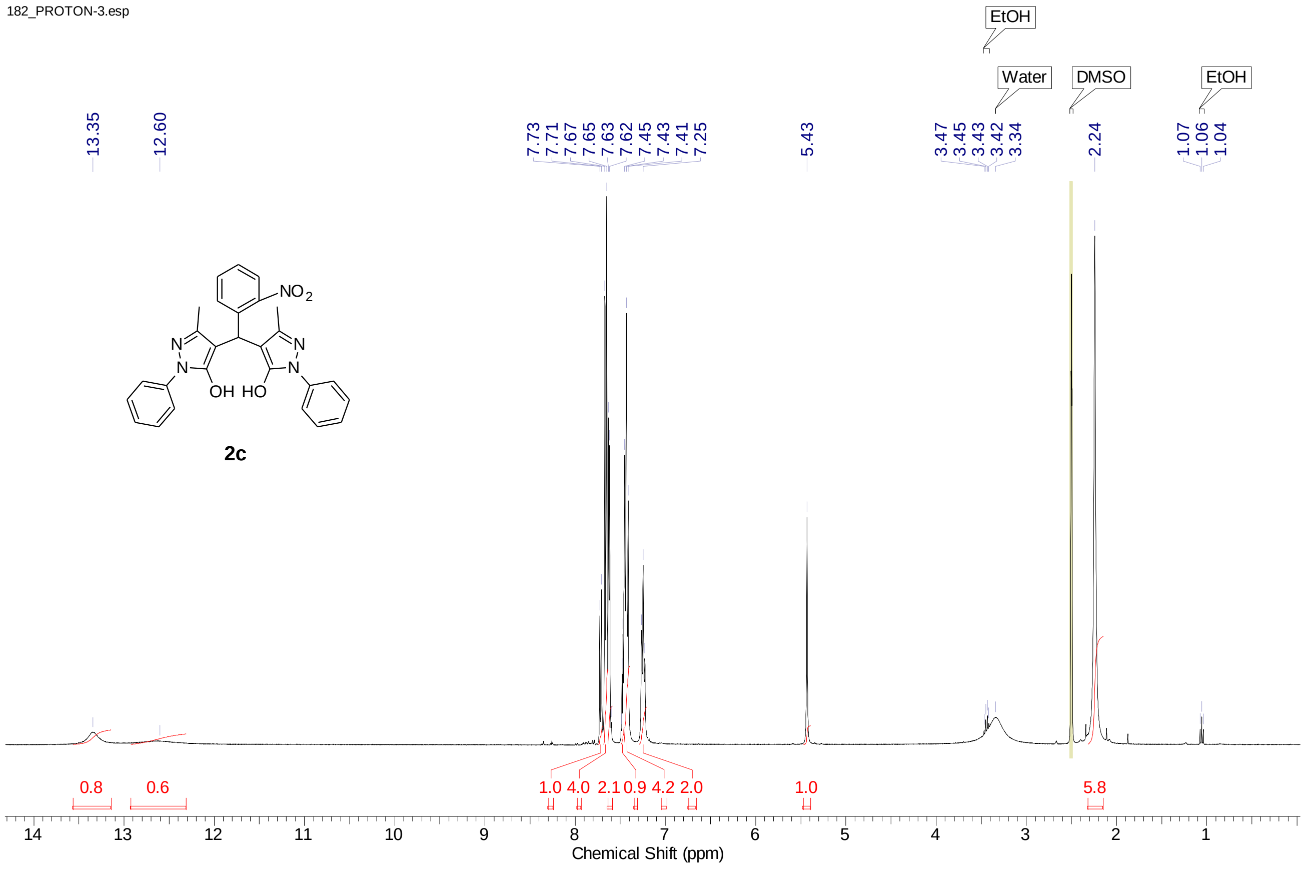

Figure S7. ^{1}H NMR spectrum of compound **2c**

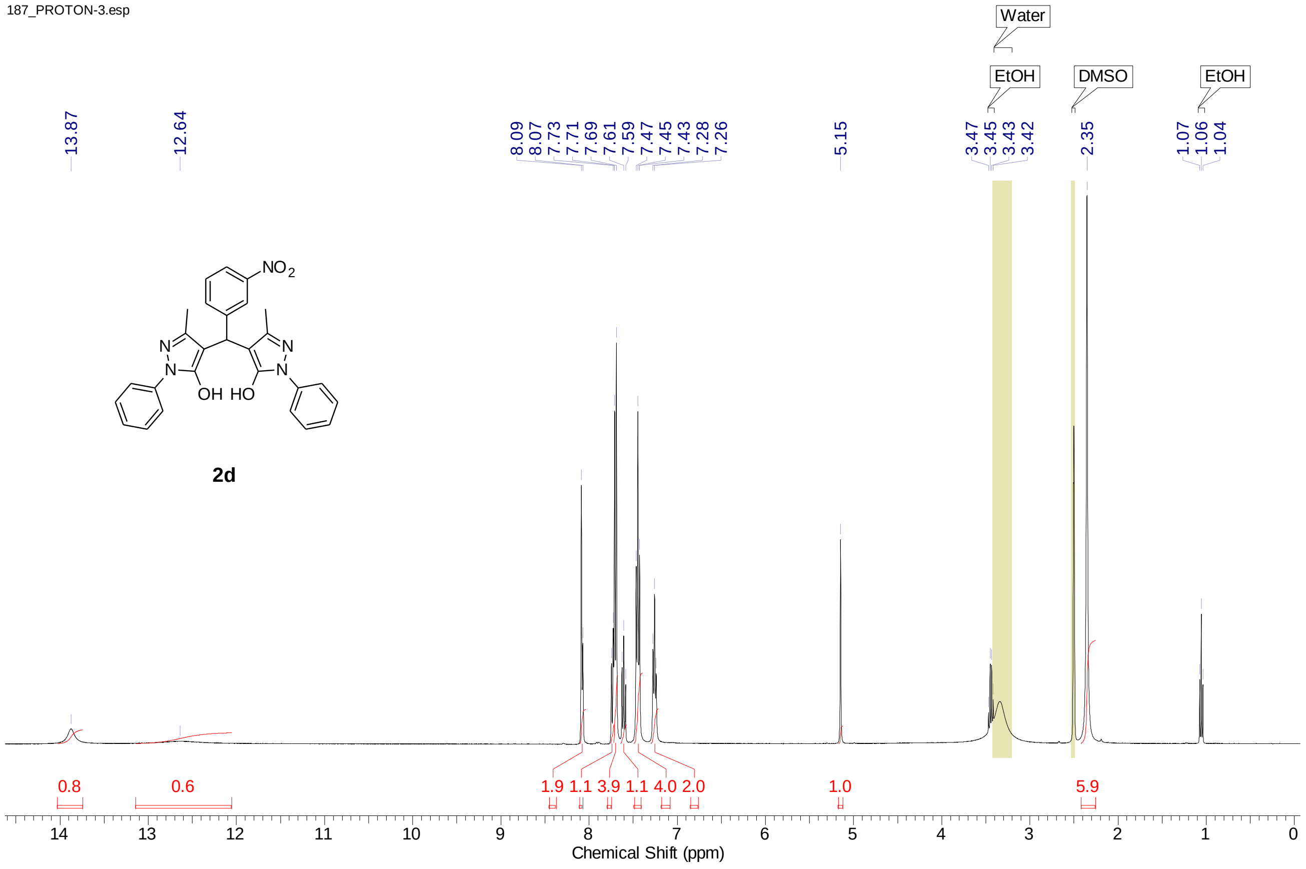

Figure S8. ^{1}H NMR spectrum of compound **2d**

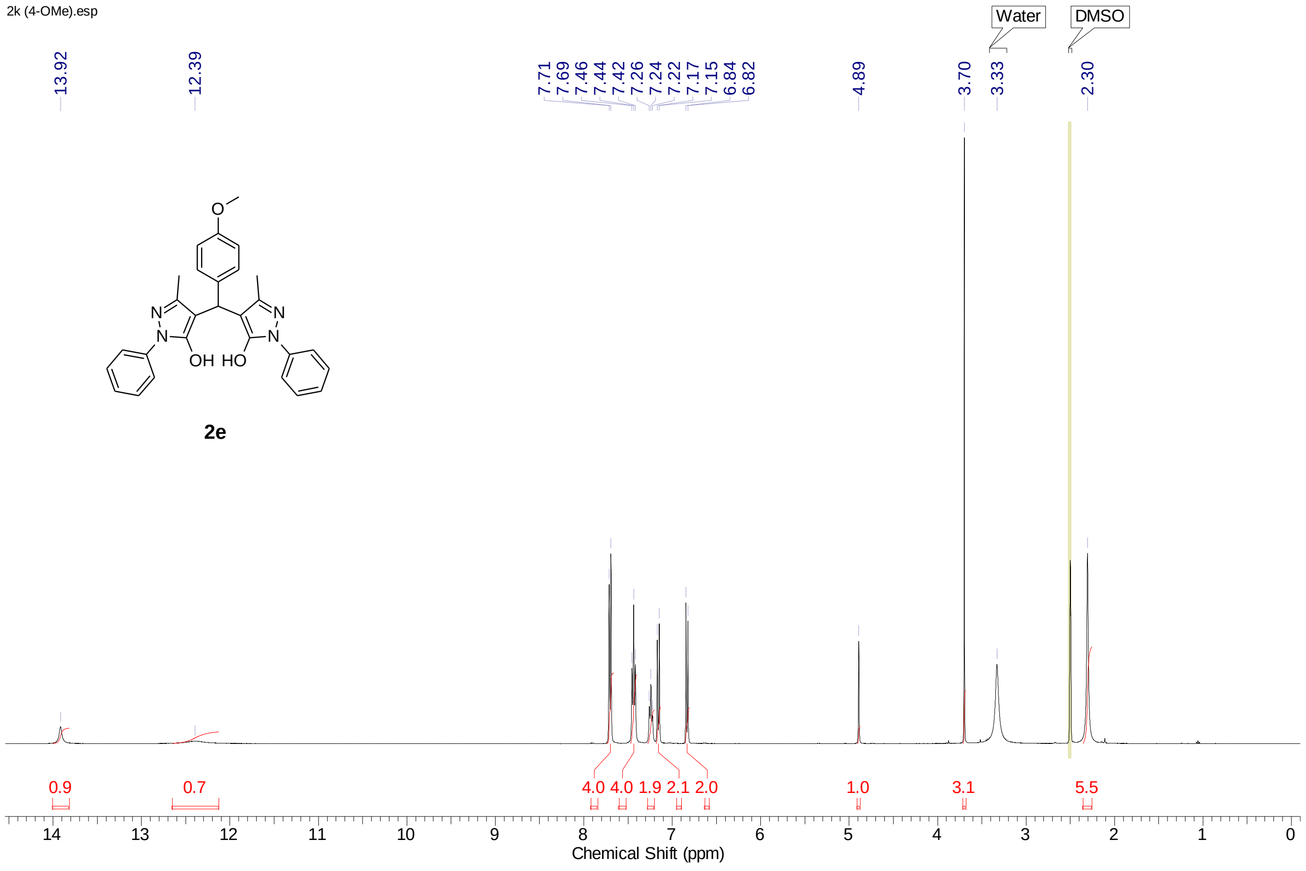

Figure S9. ^{1}H NMR spectrum of compound **2e**

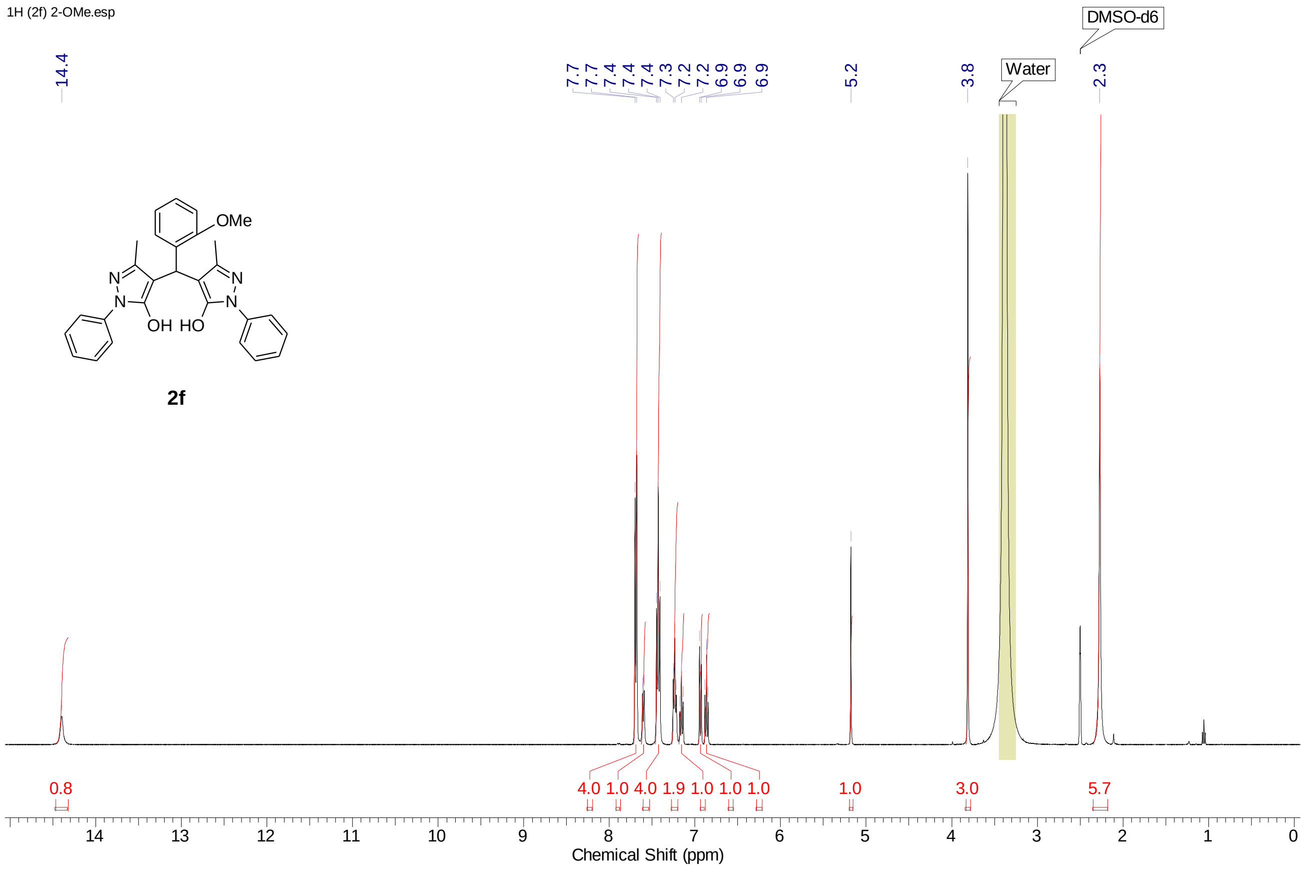

Figure S10. ^{1}H NMR spectrum of compound **2f**

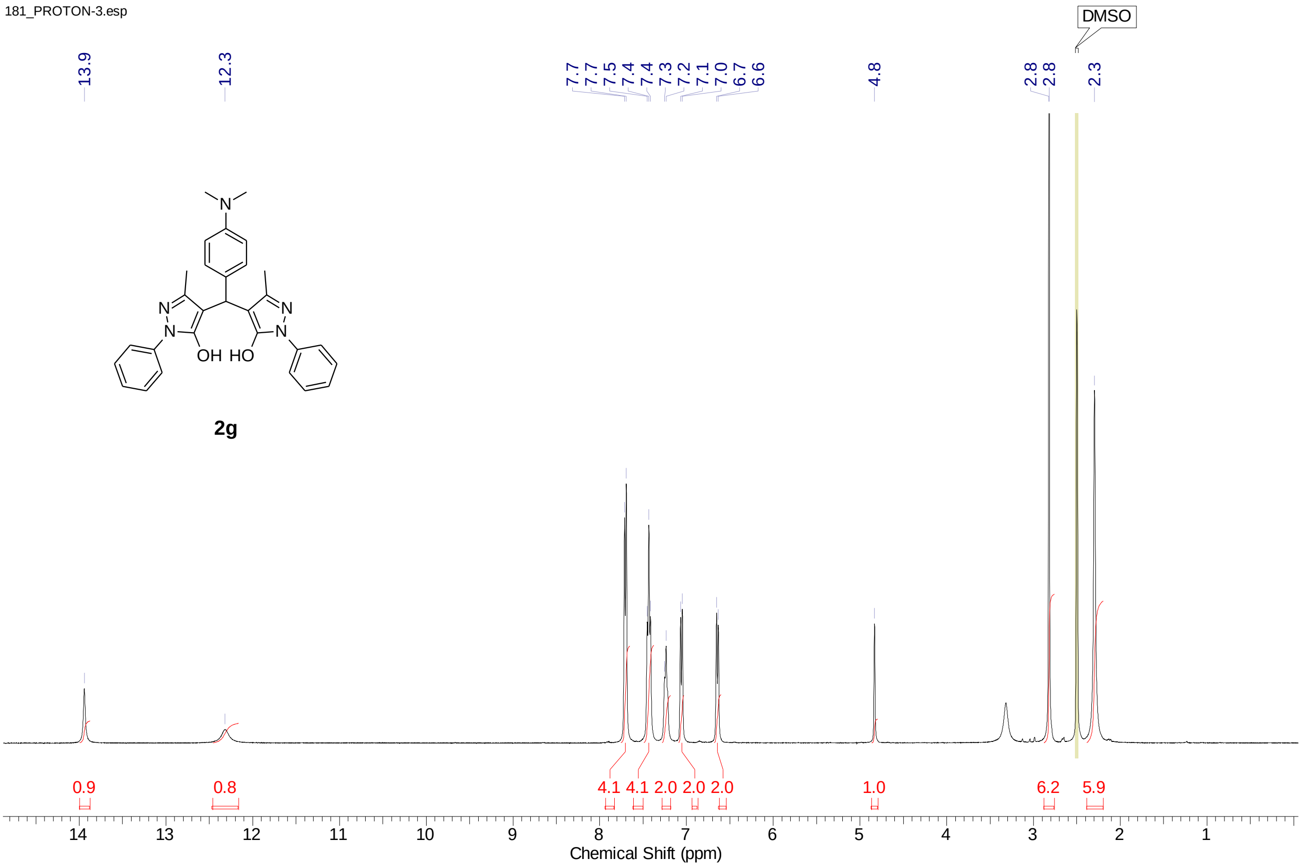

Figure S11. ^{1}H NMR spectrum of compound **2g**

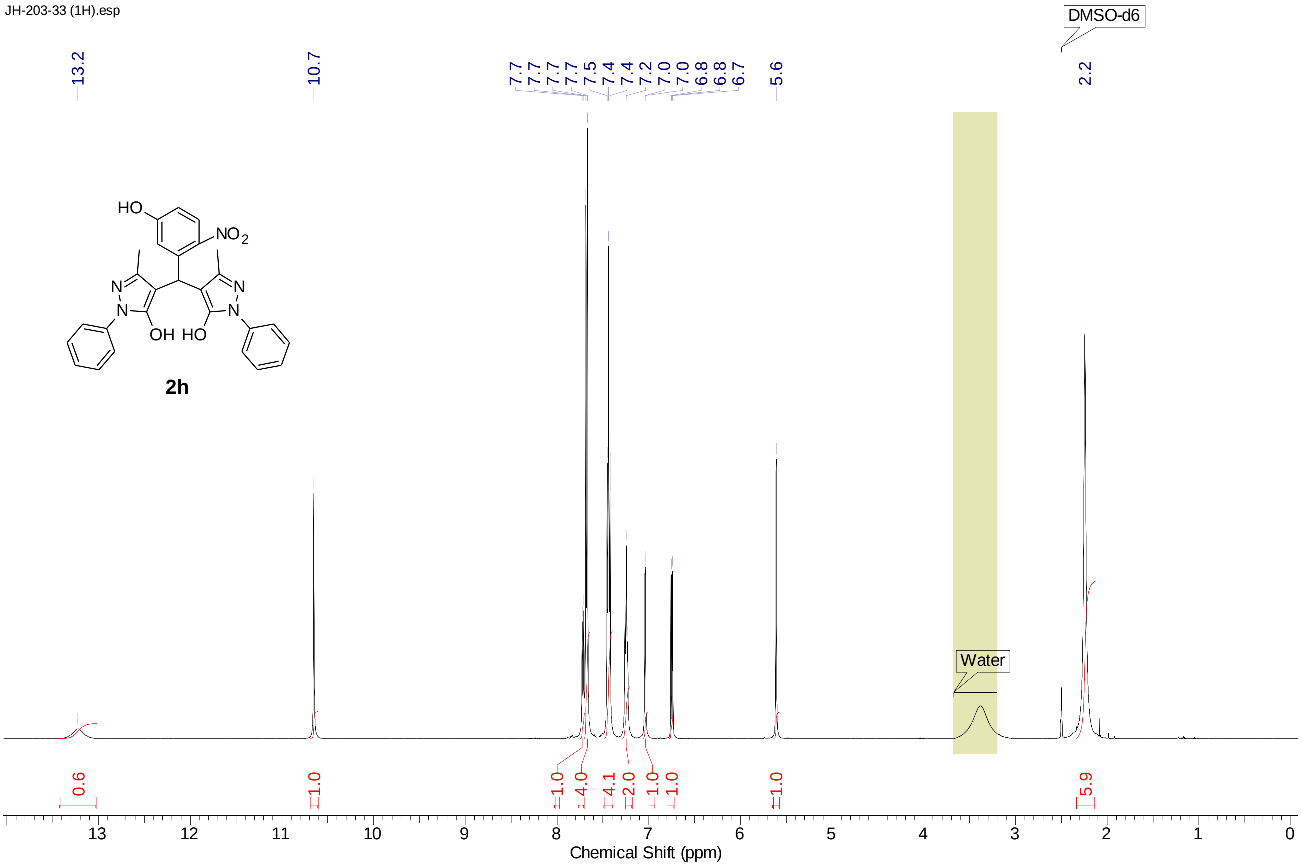

Figure S12. ^{1}H NMR spectrum of compound **2h**

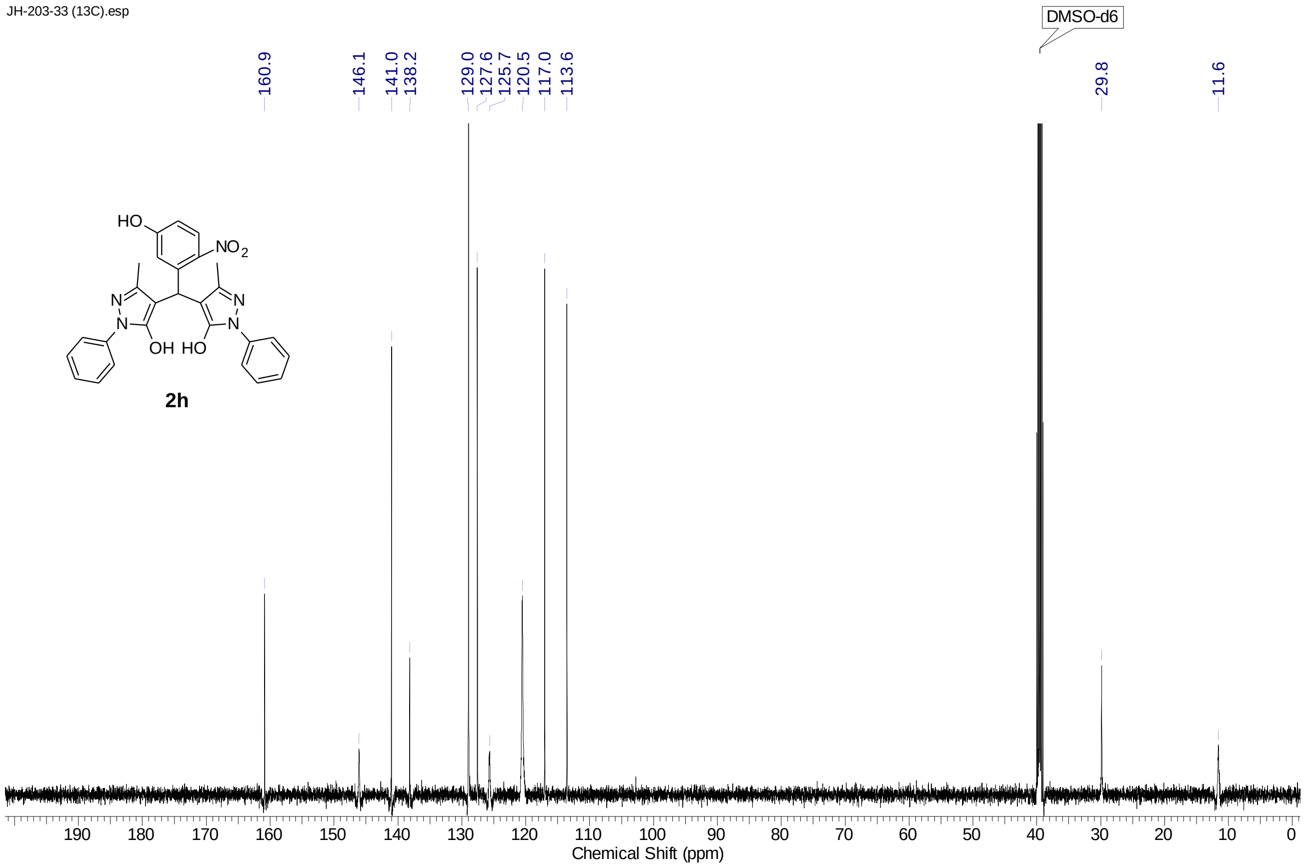

Figure S13. ^{13}C NMR spectrum of compound **2h**

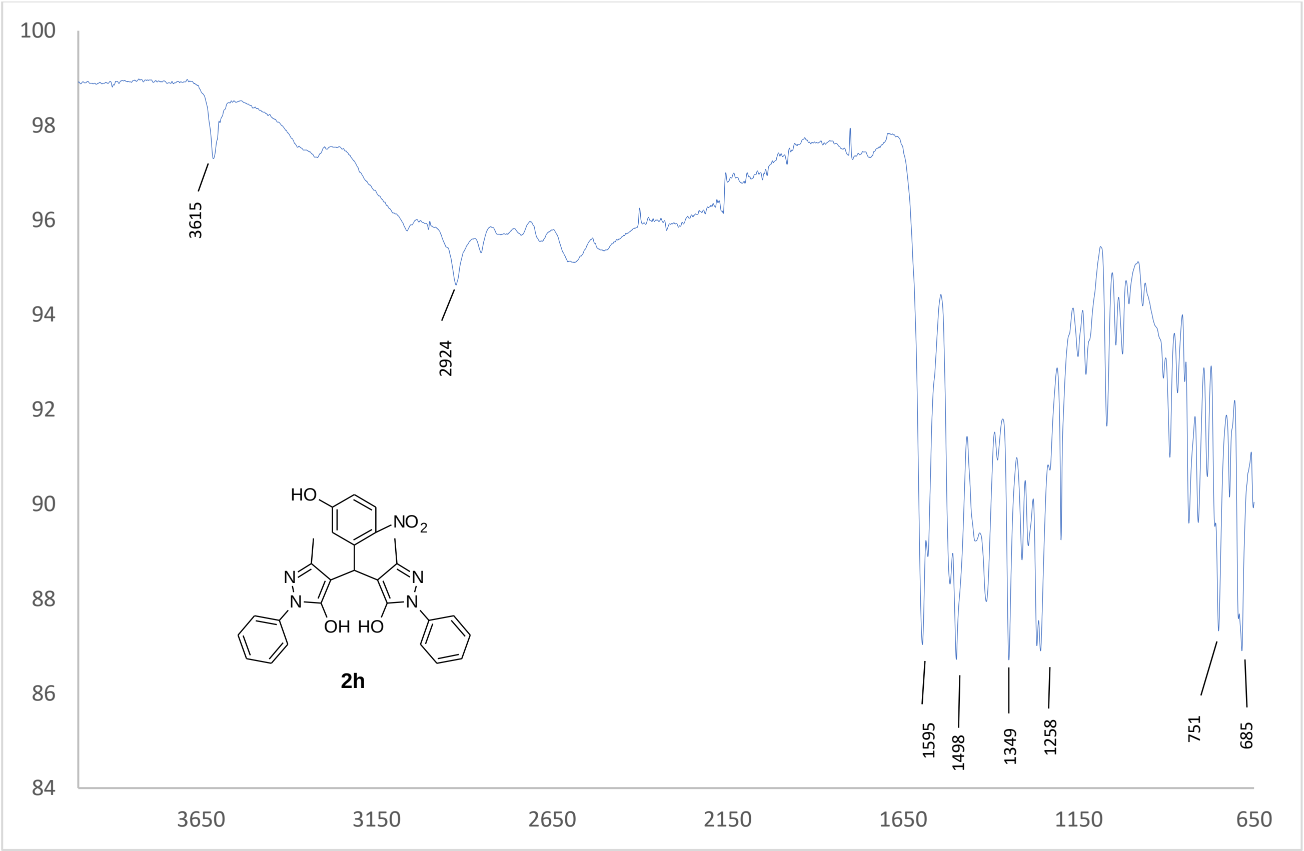

Figure S14. FTIR spectrum of compound **2h**

Monoisotopic Mass, Even Electron Ions
111 formula(e) evaluated with 4 results within limits (up to 19 closest results for each mass)
Elements Used:
C: 0-100 H: 0-200 N: 5-5 O: 0-30
24-Jun-2016
jhm-24jun16-203-33 402 (7.435) Cn (Cen,7, 50.00, Ar); Sm (SG, 3x5.00); Sb (12,5.00)

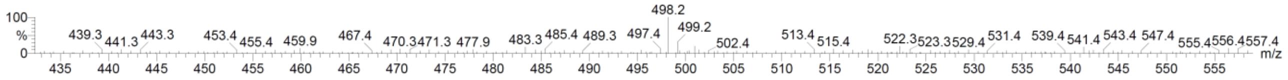

Minimum:				-2.0		
Maximum:		10.0	10.0	1000.0		
Mass	Calc. Mass	mDa	PPM	DBE	i-FIT	Formula
498.1772	498.1777	-0.5	-1.0	18.5	0.8	C27 H24 N5 O5
	498.1719	5.3	10.6	27.5	40.7	C34 H20 N5
	498.1836	-6.4	-12.8	9.5	33.4	C20 H28 N5 O10
	498.1684	8.8	17.7	5.5	92.6	C16 H28 N5 O13

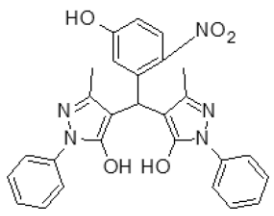

Molecular Formula = $C_{27}H_{23}N_5O_5$
[M+H]+ = 498.177195 Da

Figure S15. HRMS spectrum of compound **2h**

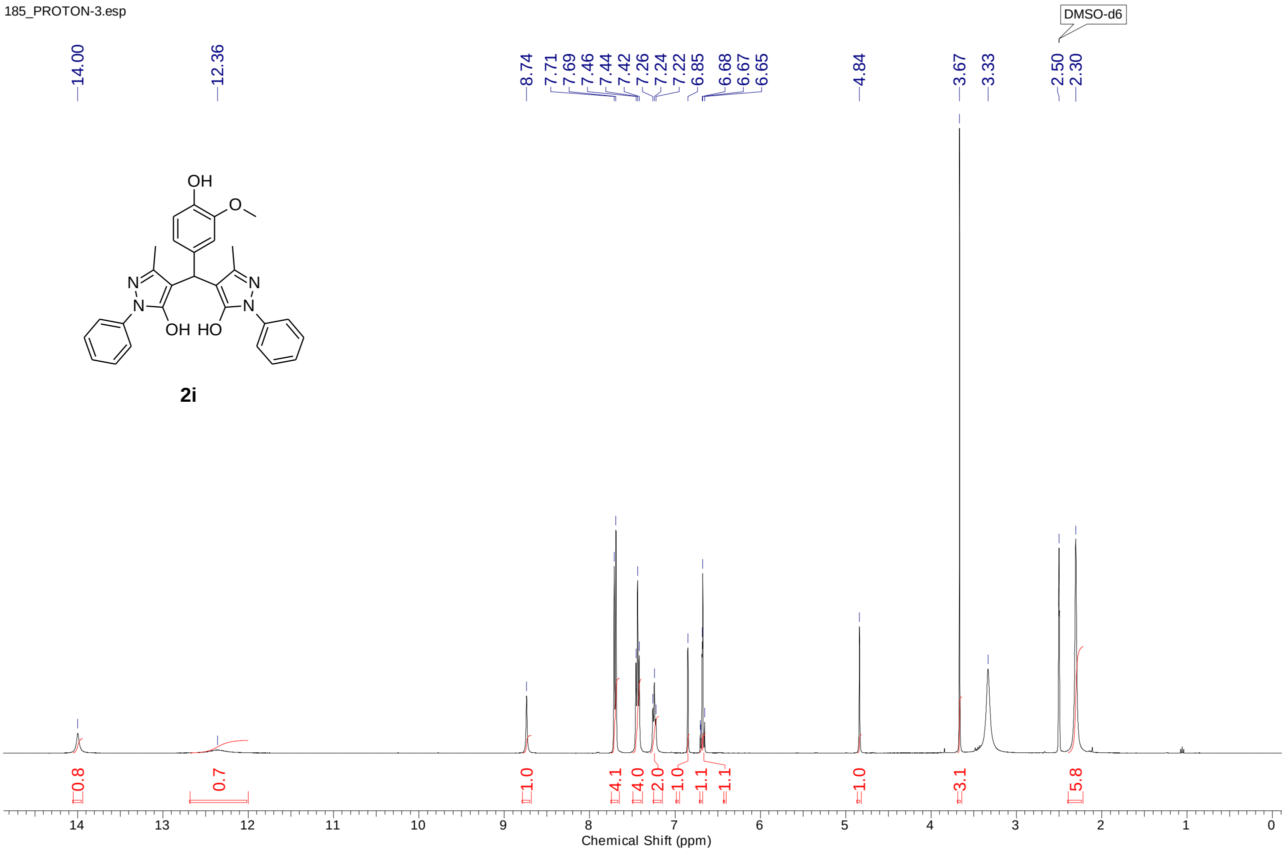

Figure S16. ^{1}H NMR spectrum of compound **2i**.

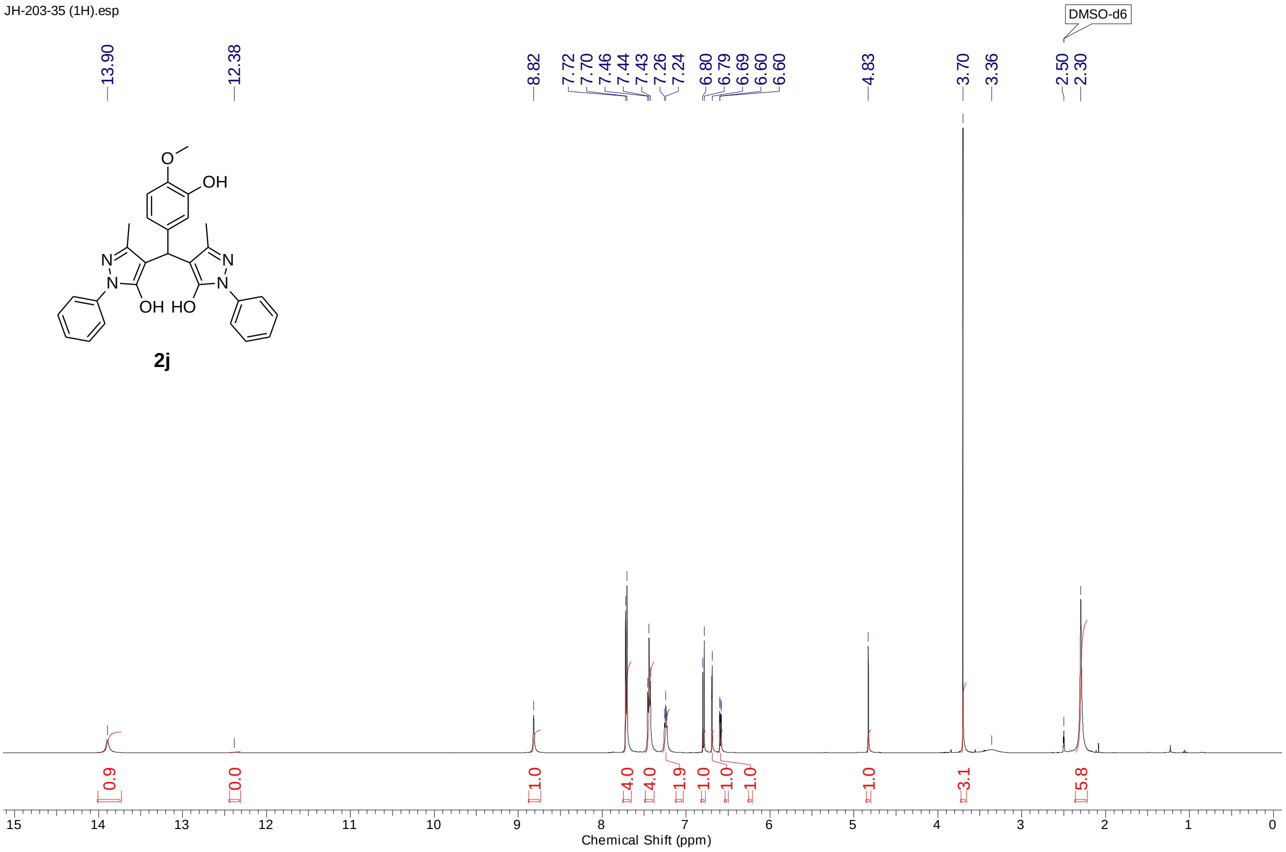

Figure S17. ^{1}H NMR spectrum of compound **2j**.

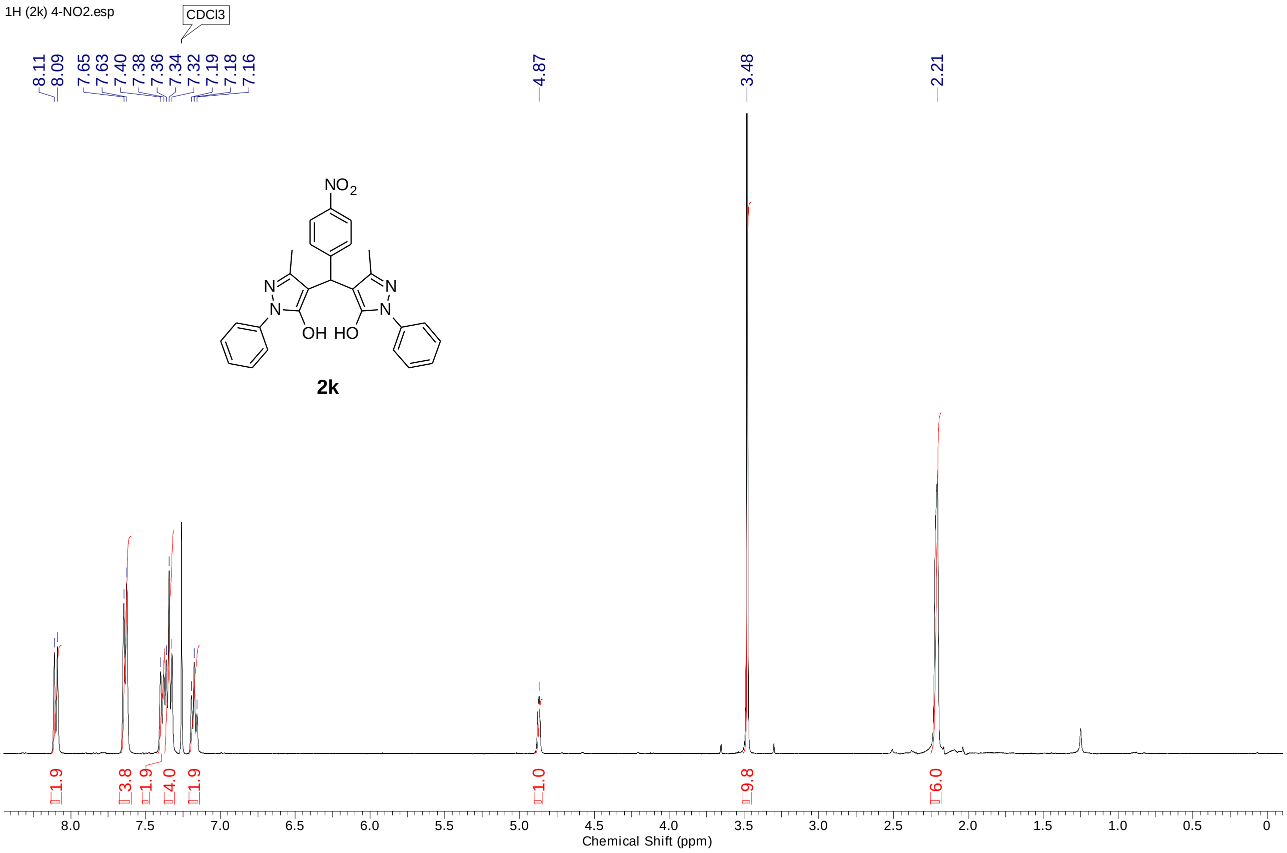

Figure S18. ^{1}H NMR spectrum of compound **2k**.

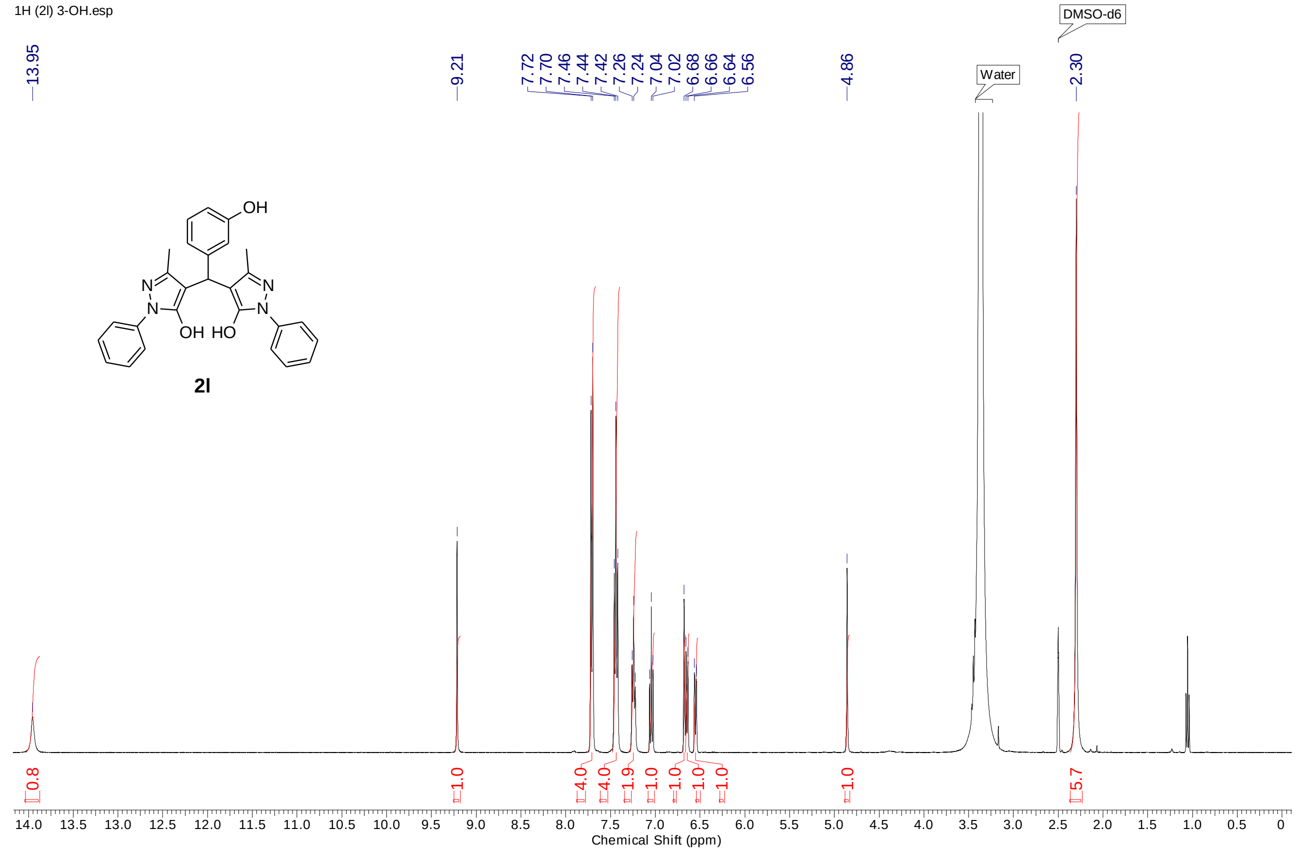

Figure S19. ^{1}H NMR spectrum of compound **2l**.

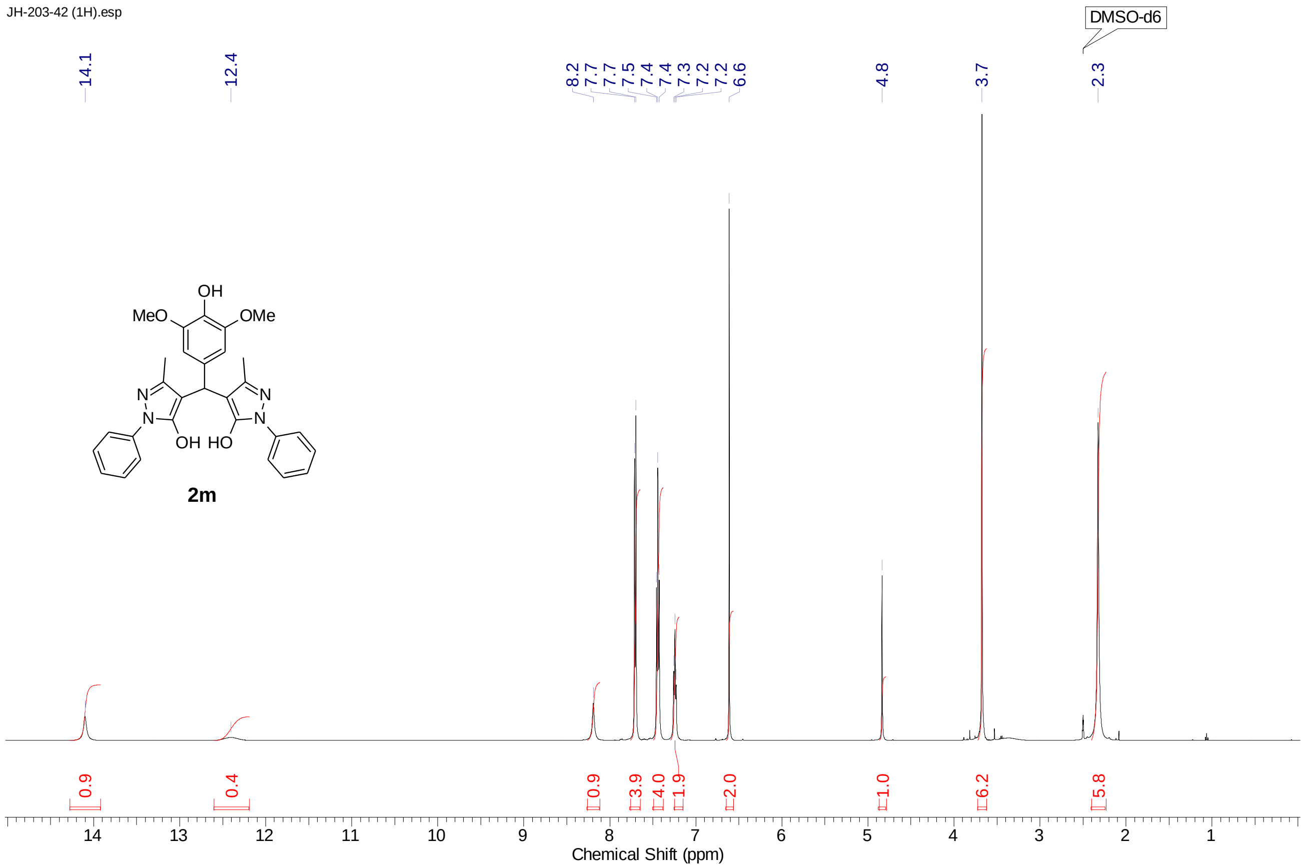

Figure S20. ^{1}H NMR spectrum of compound **2m**

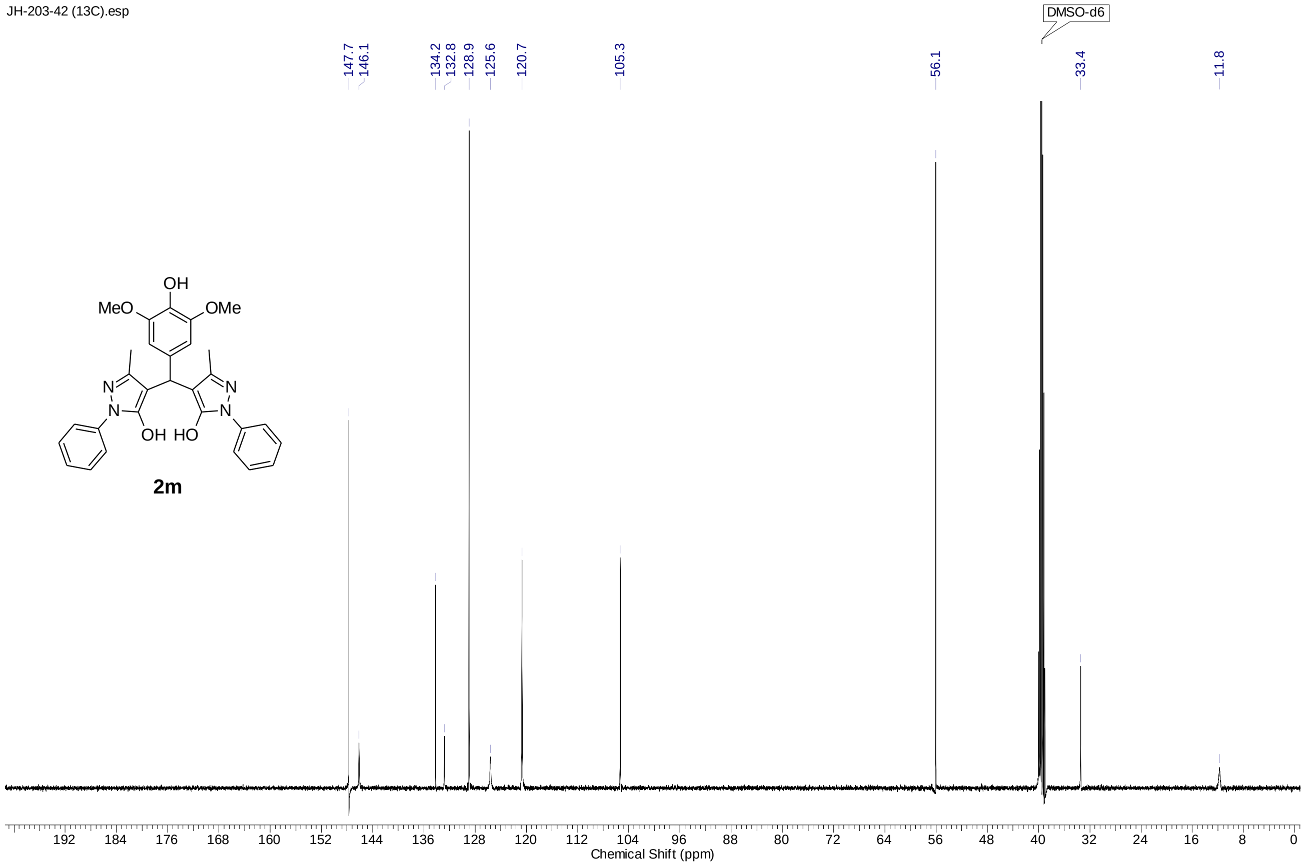

Figure S21. ^{13}C NMR spectrum of compound **2m**

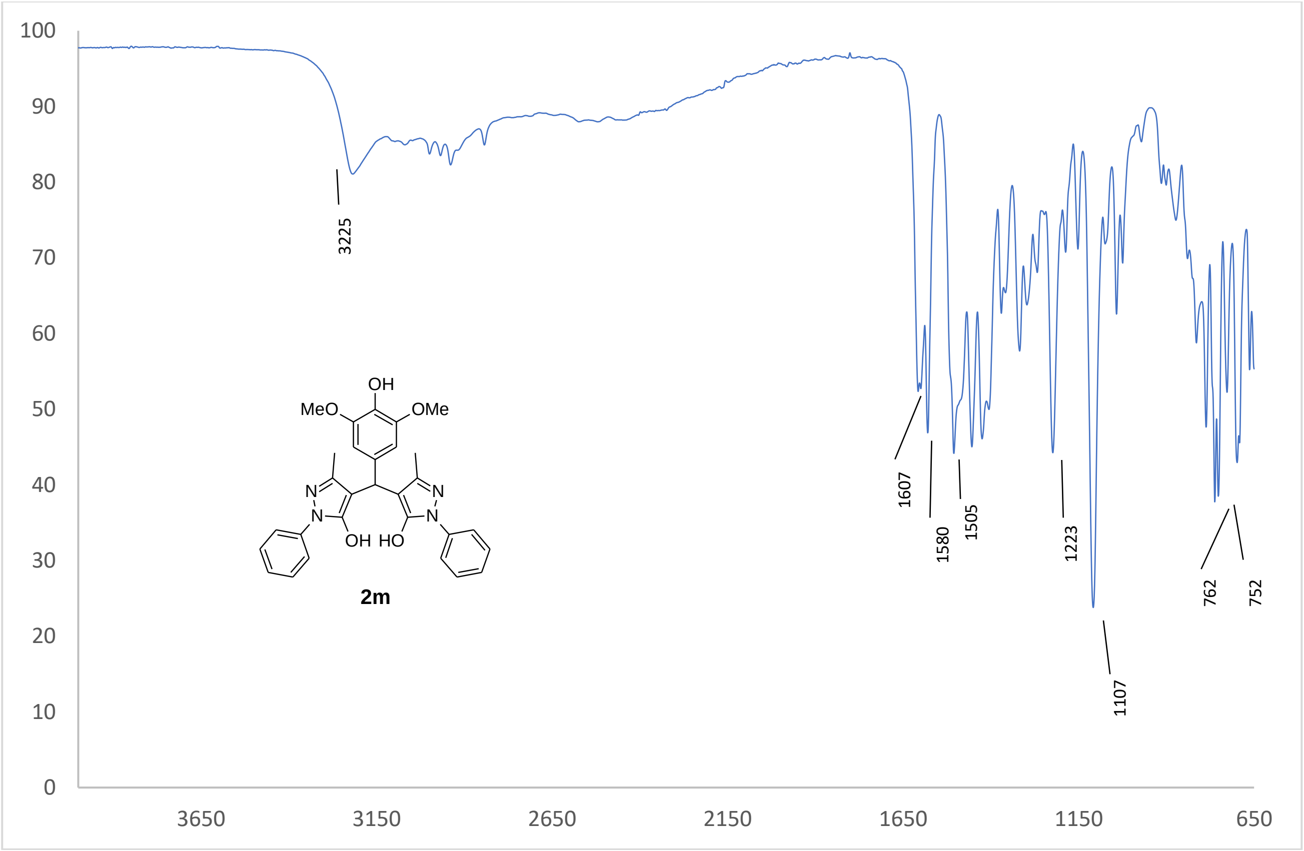

Figure S22. FTIR spectrum of compound **2m**

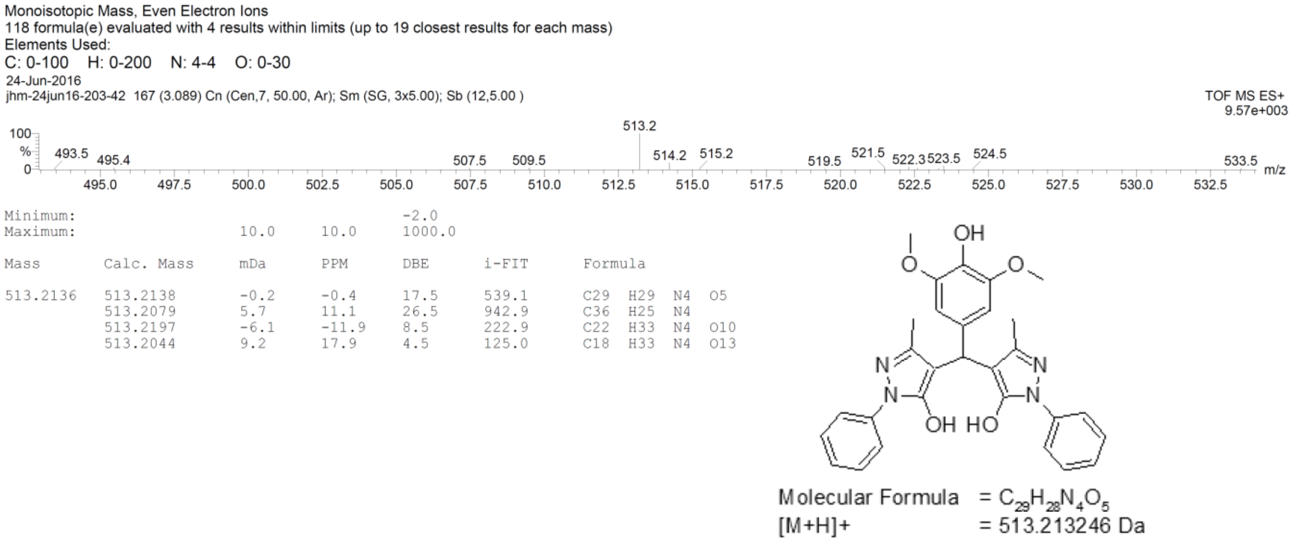

Figure S23. HRMS spectrum of compound **2m**

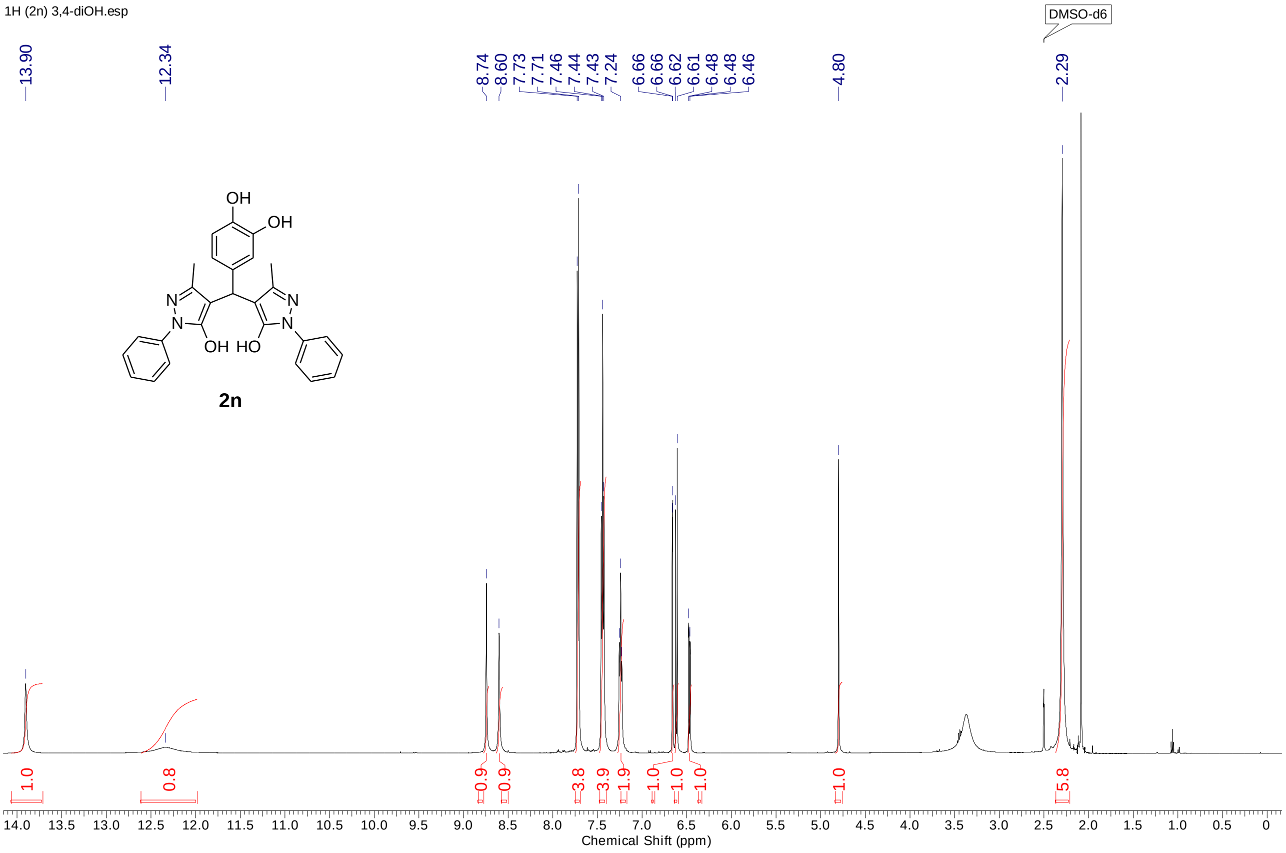

Figure S24. ^{1}H NMR spectrum of compound **2n**.

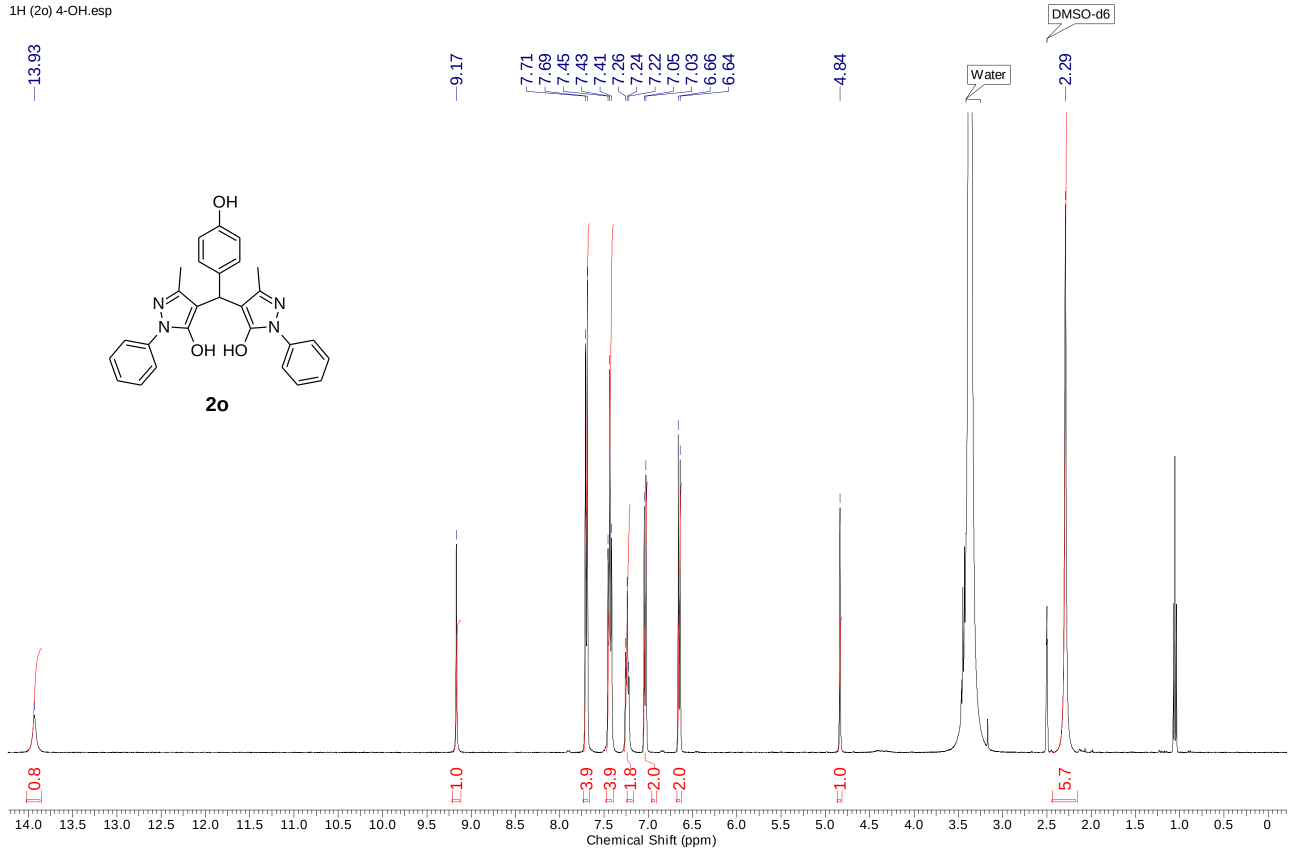

Figure S25. ¹H NMR spectrum of compound **2o**.

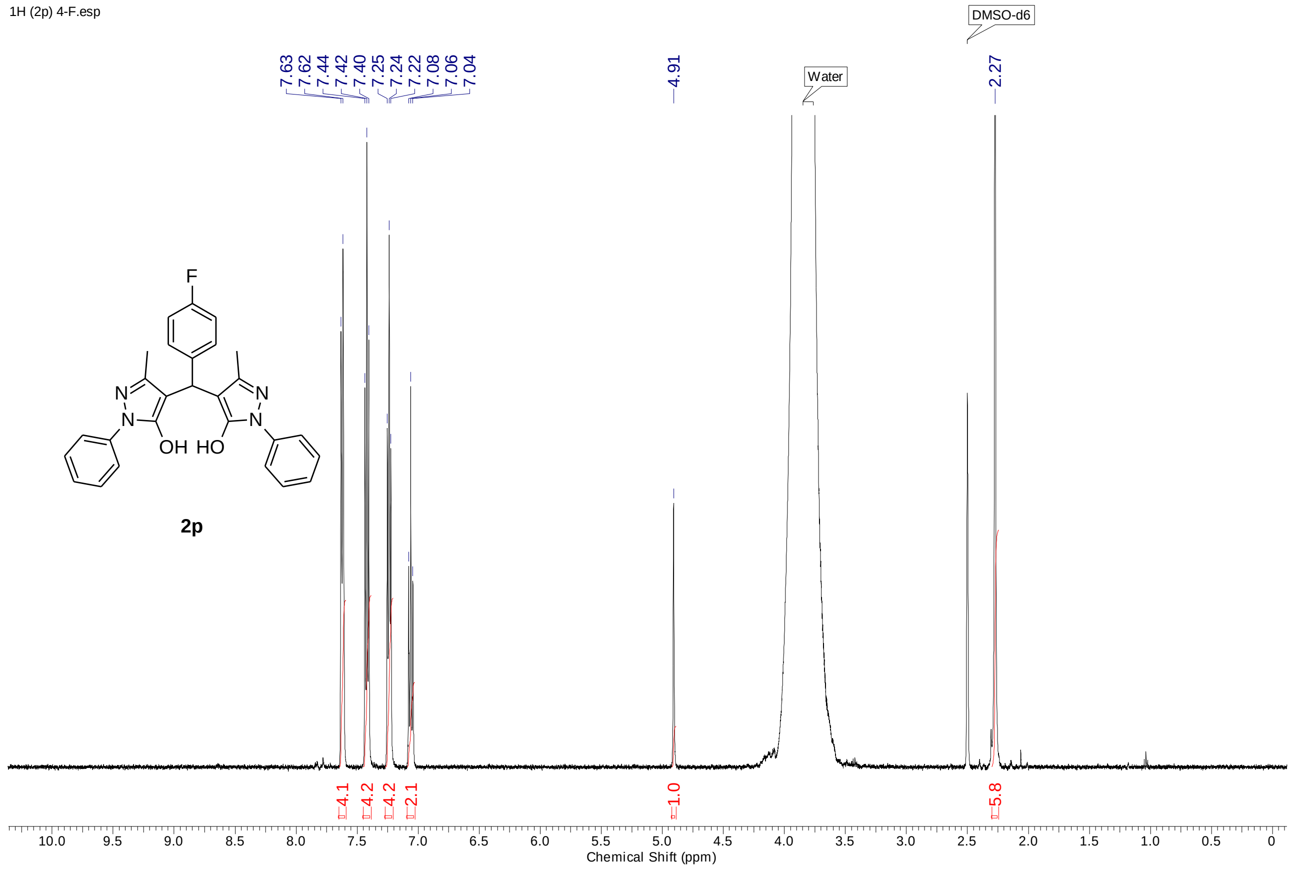

Figure S26. ¹H NMR spectrum of compound **2p**.

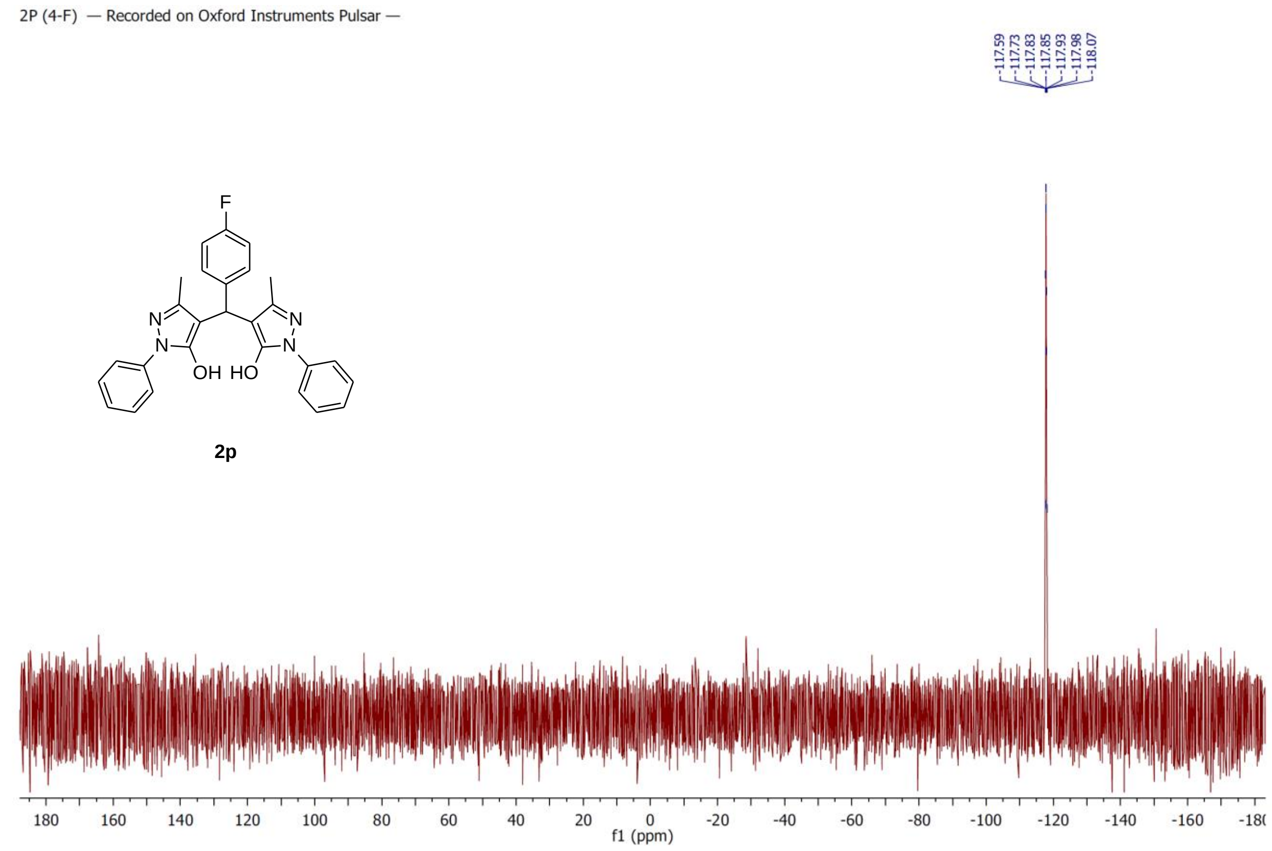

Figure S27. ^{19}F NMR spectrum of compound **2p**.

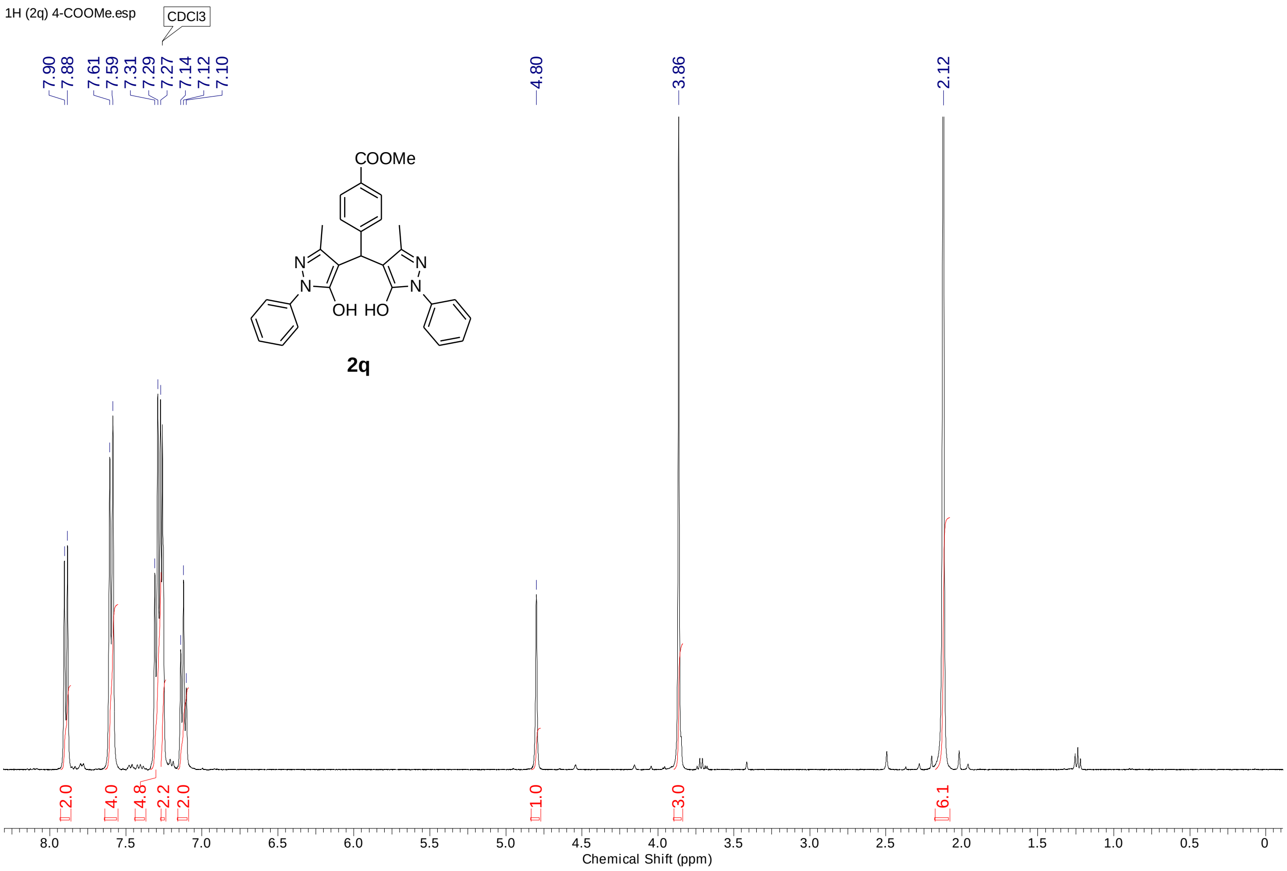

Figure S28. ^{1}H NMR spectrum of compound **2q**.

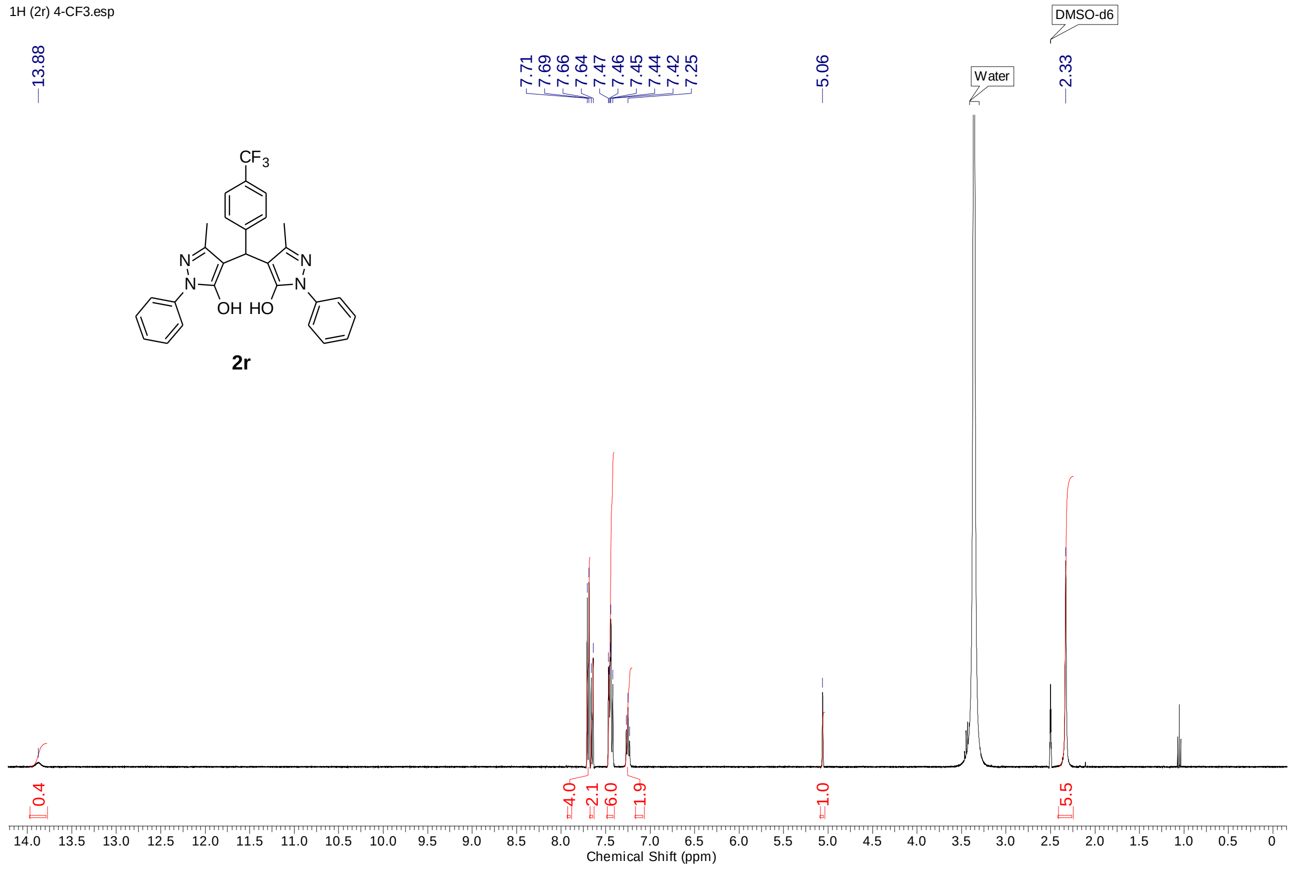

Figure S29. ^{1}H NMR spectrum of compound **2r**.

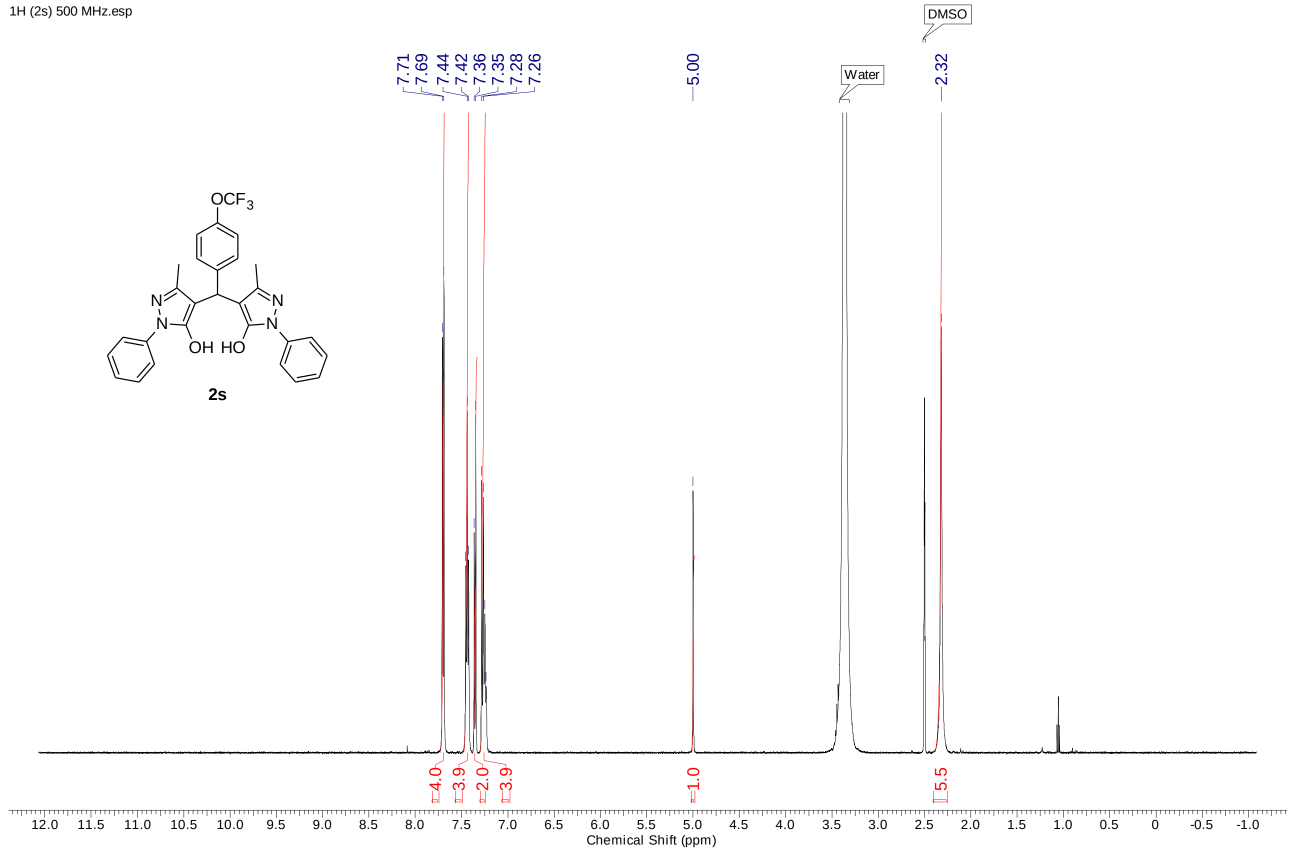

Figure S30. ^{1}H NMR spectrum of compound **2s**.

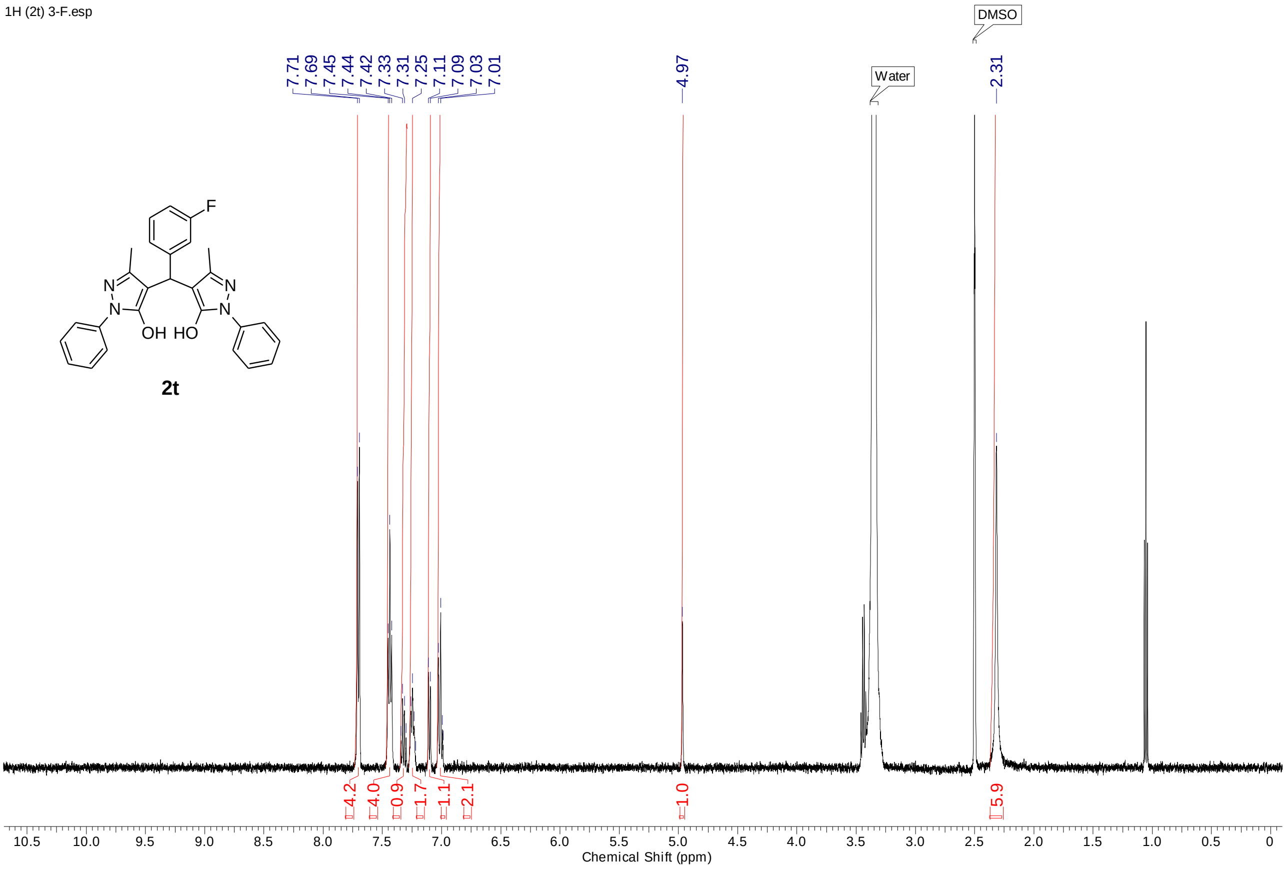

Figure S31. ^{1}H NMR spectrum of compound **2t**.

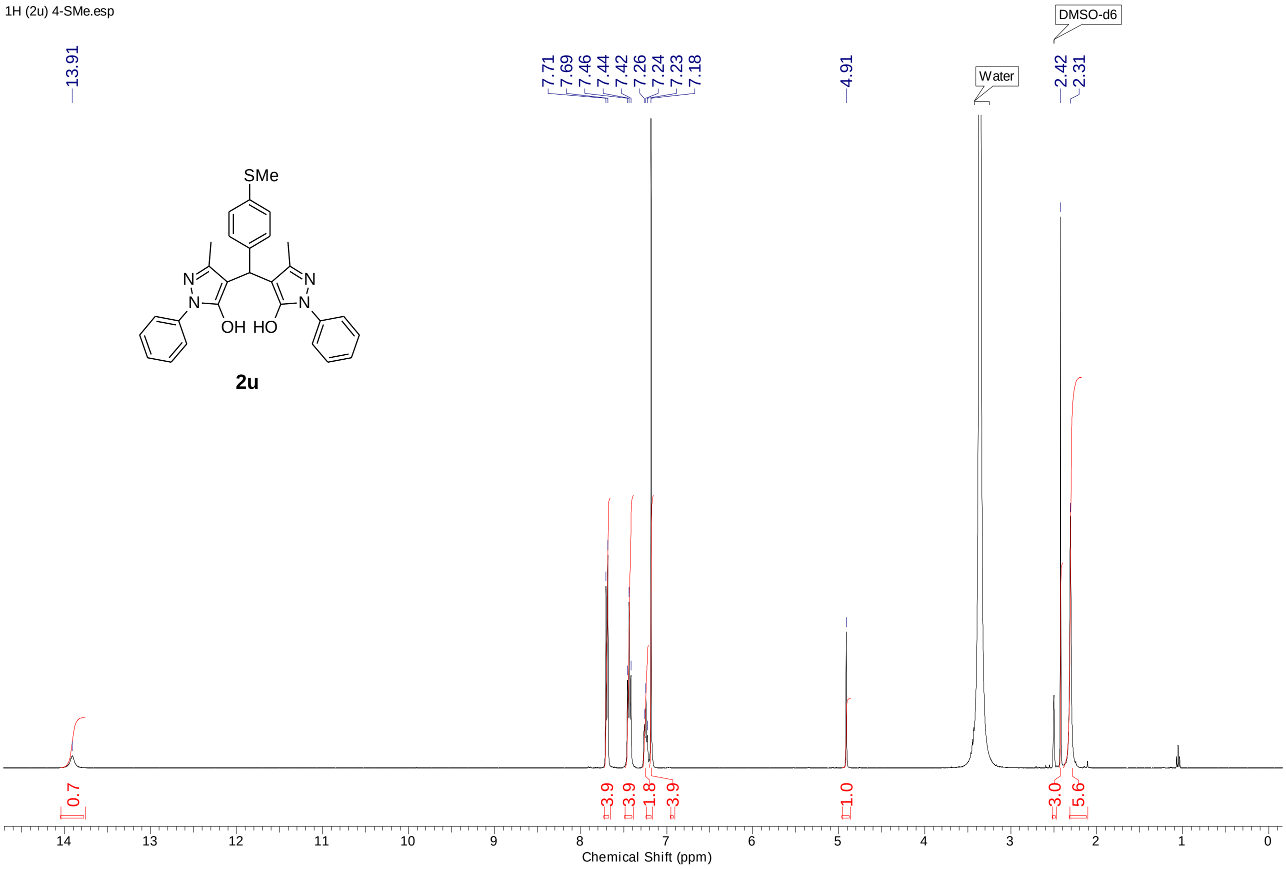

Figure S32. ^{1}H NMR spectrum of compound **2u**.

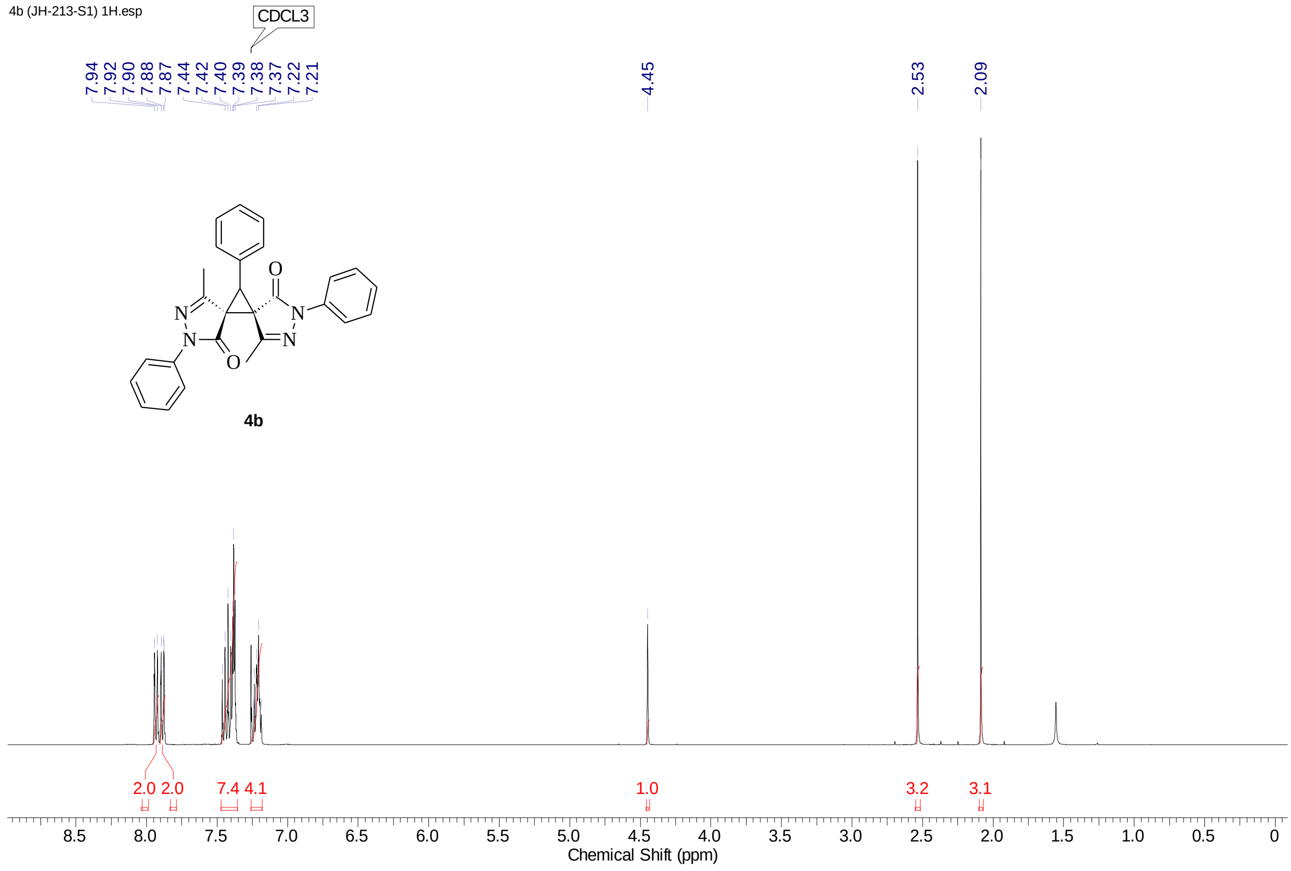

Figure S33. ^{1}H NMR spectrum of compound **4b**

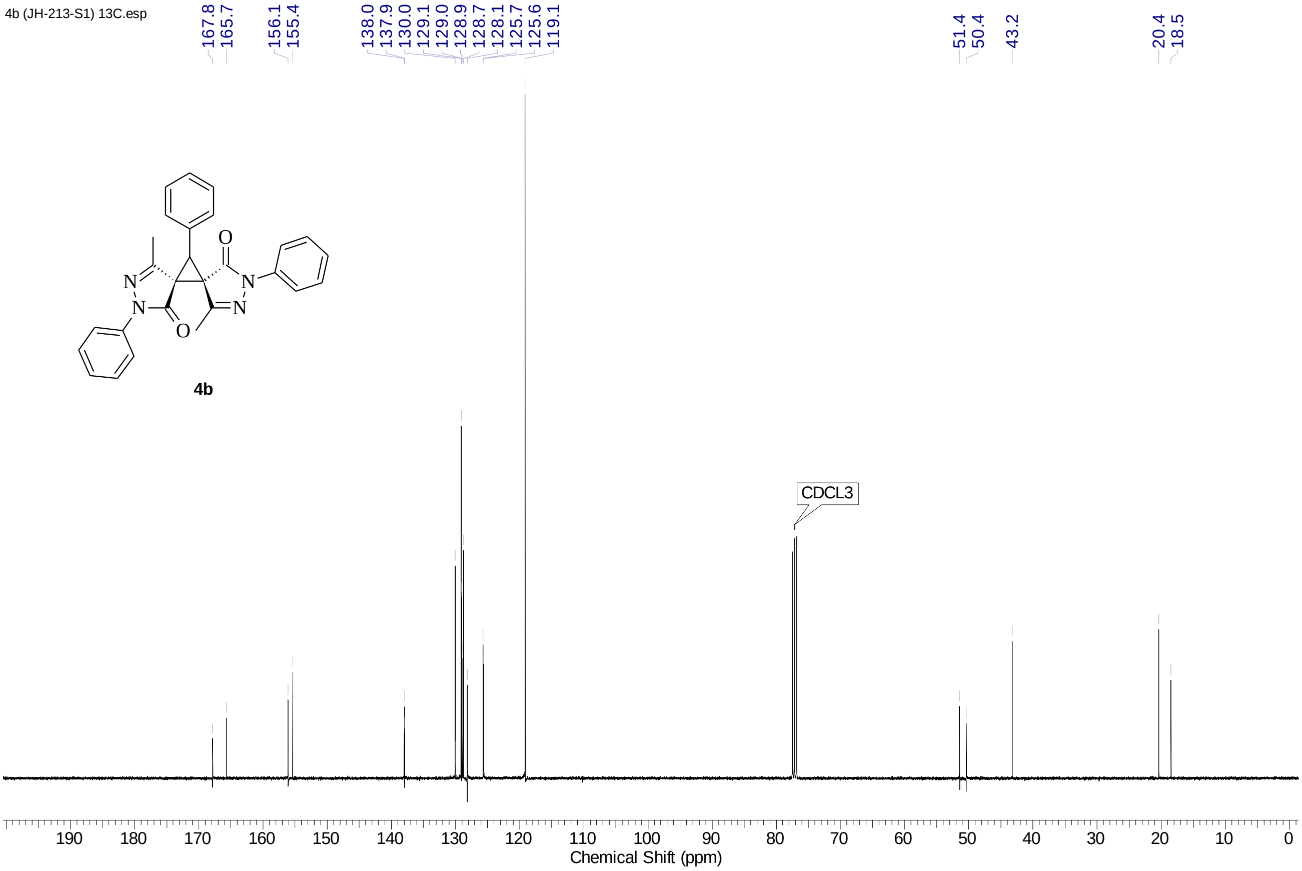

Figure S34. ^{13}C NMR spectrum of compound **4b**

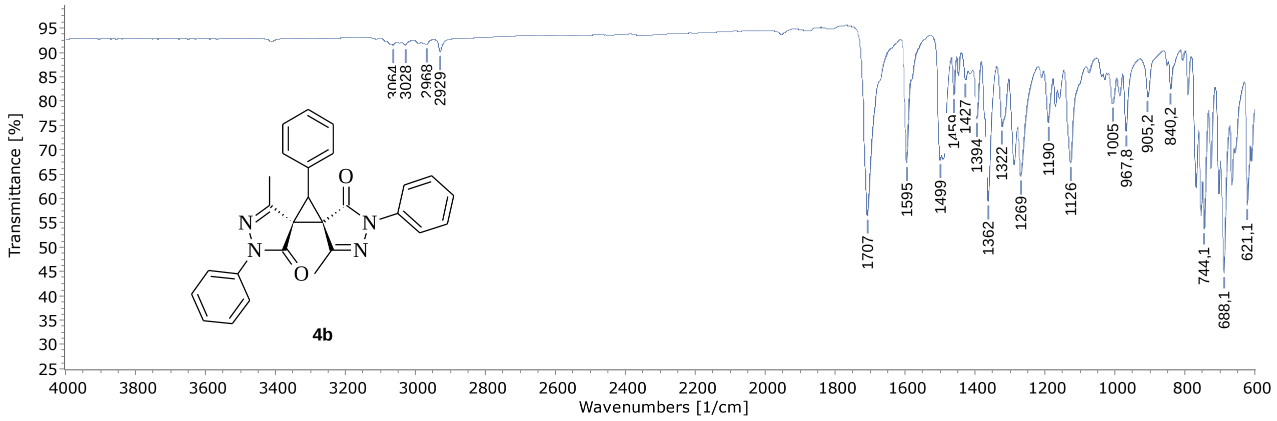

Figure S35. FTIR spectrum of compound **4b**

Acquisition Parameter

Ion Source Type	ESI	Ion Polarity	Positive	Alternating Ion Polarity	off
Mass Range Mode	UltraScan	Scan Begin	70 m/z	Scan End	450 m/z
Accumulation Time	200000 µs	RF Level	63 %	Trap Drive	54.1
SPS Target Mass	400 m/z	Averages	5 Spectra	n/a	n/a

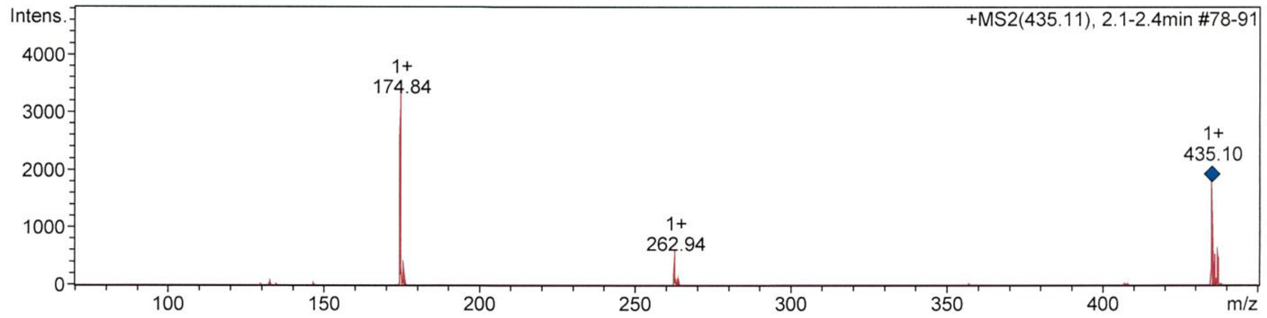

Component	Molecular Mass	Molecule		Absolute Abundance	Relative Abundance
A	174.84	174.93	[M + H]+	3230	100.00
B	435.11	435.30	[M + H]+	1784	55.22
C	262.94	263.10	[M + H]+	521	16.14

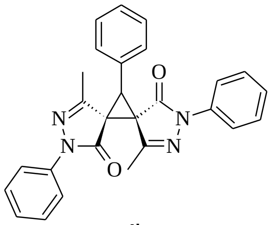

4b

Figure S36. ESI-MS spectrum of compound **4b**

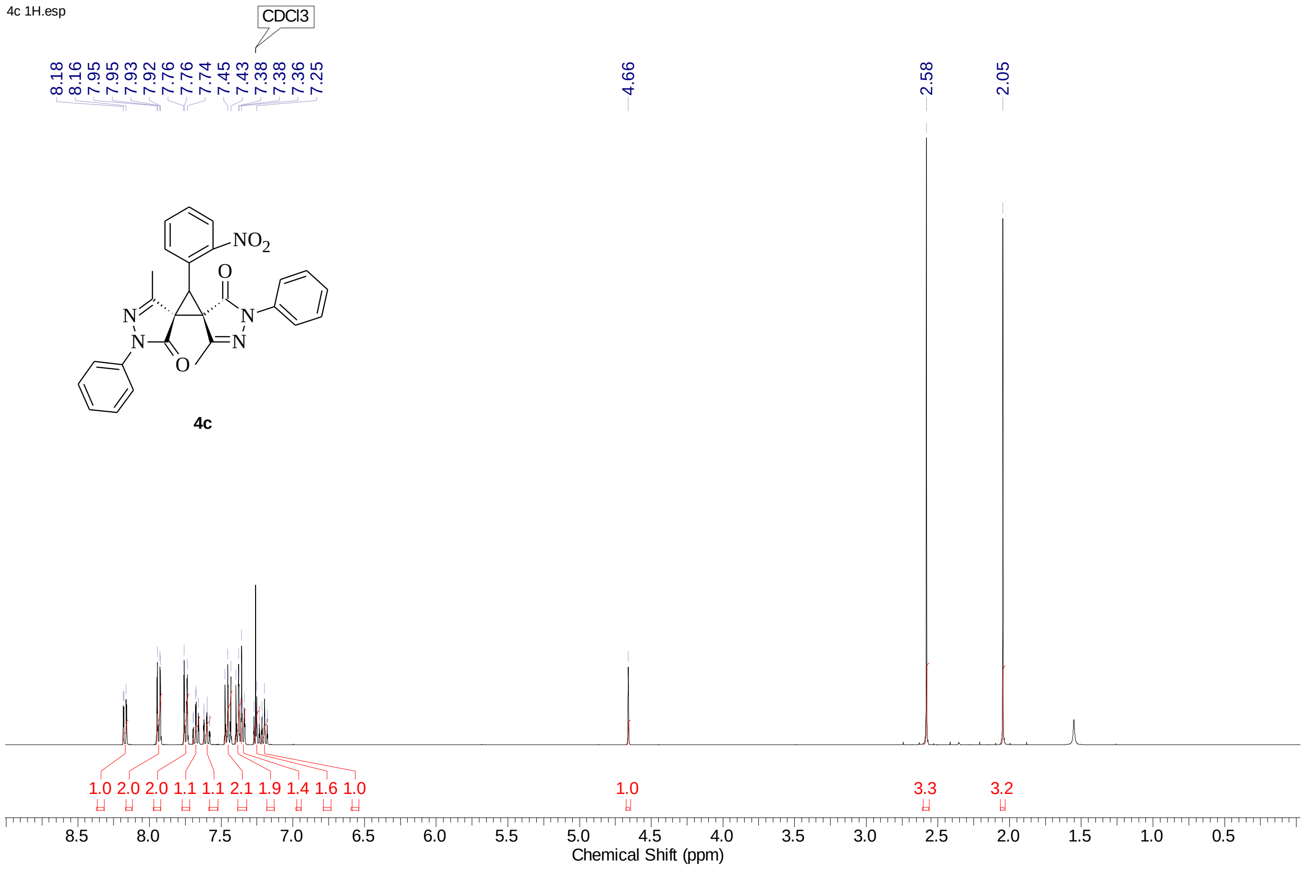

Figure S37. ^{1}H NMR spectrum of compound **4c**

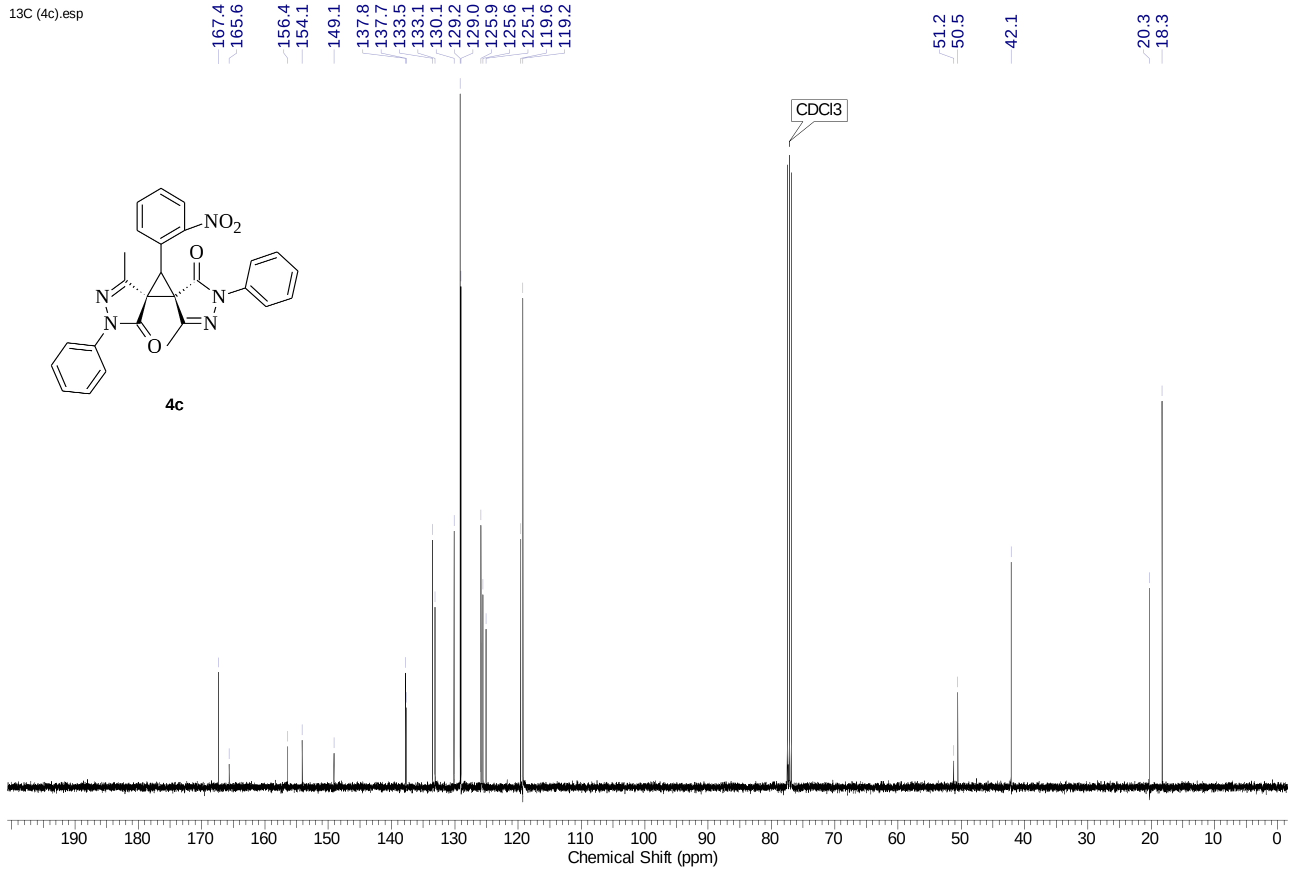

Figure S38. ^{13}C NMR spectrum of compound **4c**

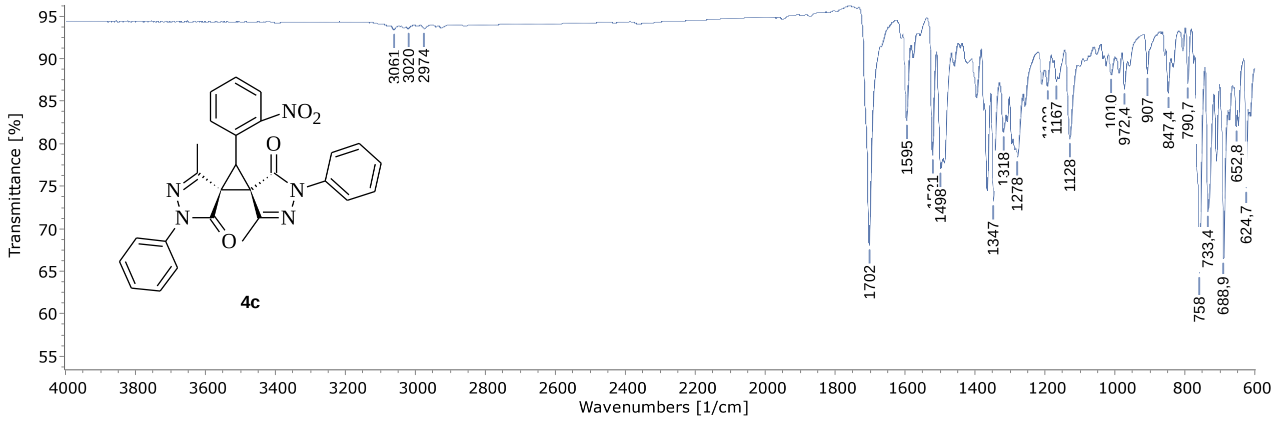

Figure S39. FTIR spectrum of compound **4c**

Acquisition Parameter

Ion Source Type	ESI	Ion Polarity	Positive	Alternating Ion Polarity	off
Mass Range Mode	UltraScan	Scan Begin	70 m/z	Scan End	600 m/z
Accumulation Time	200000 µs	RF Level	63 %	Trap Drive	54.1
SPS Target Mass	400 m/z	Averages	5 Spectra	n/a	n/a

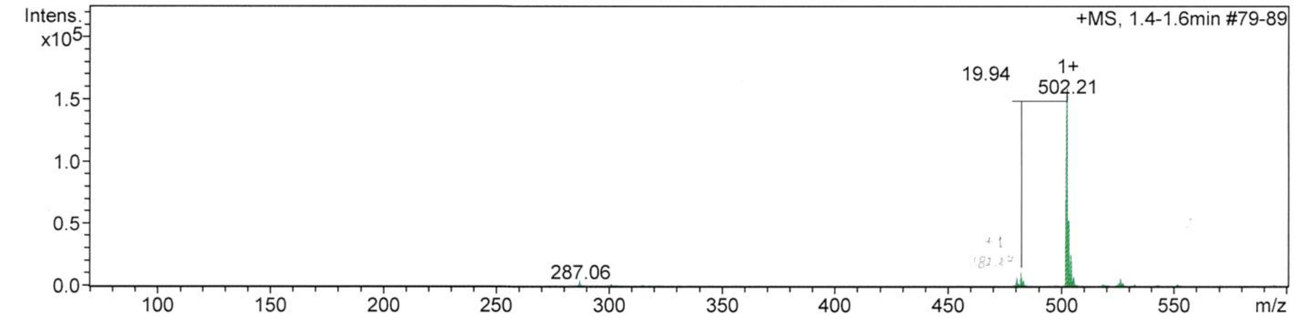

Component	Molecular Mass	Molecule		Absolute Abundance	Relative Abundance
A	502.18	502.66	[M + H]+	150245	100.00
B	482.27	482.67	[M + H]+	8078	5.38

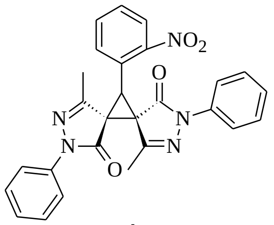

4c

Figure S40. ESI-MS spectrum of compound **4c**

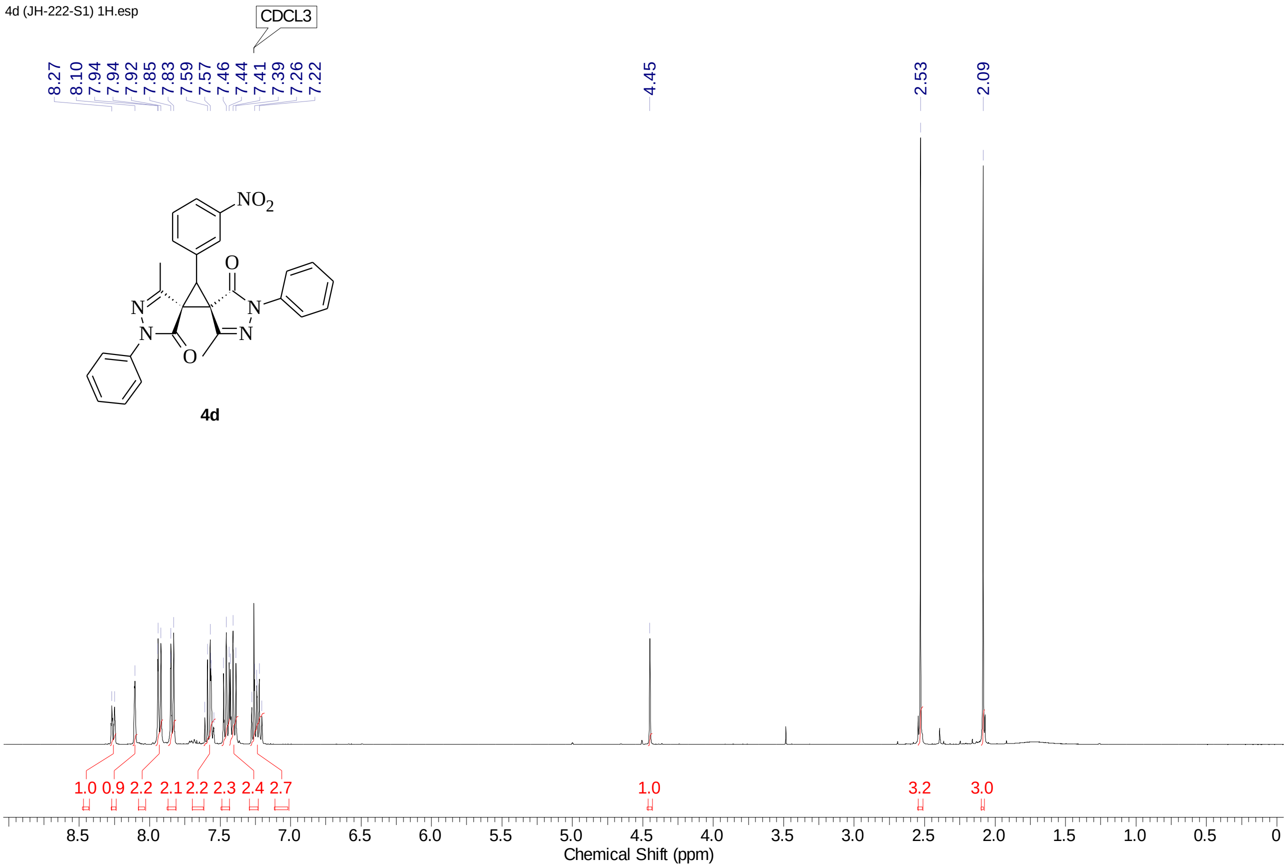

Figure S41. ^{1}H NMR spectrum of compound **4d**

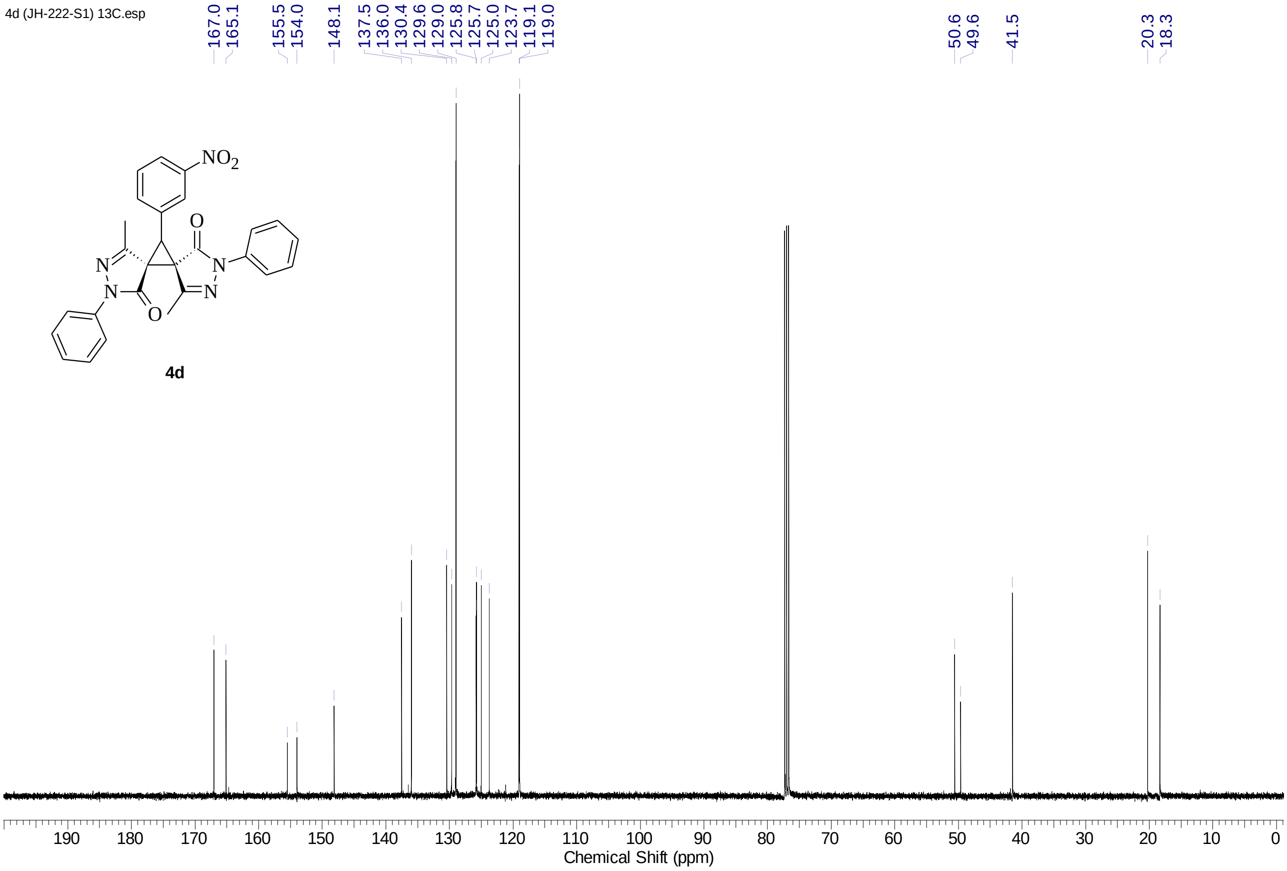

Figure S42. ^{13}C NMR spectrum of compound **4d**

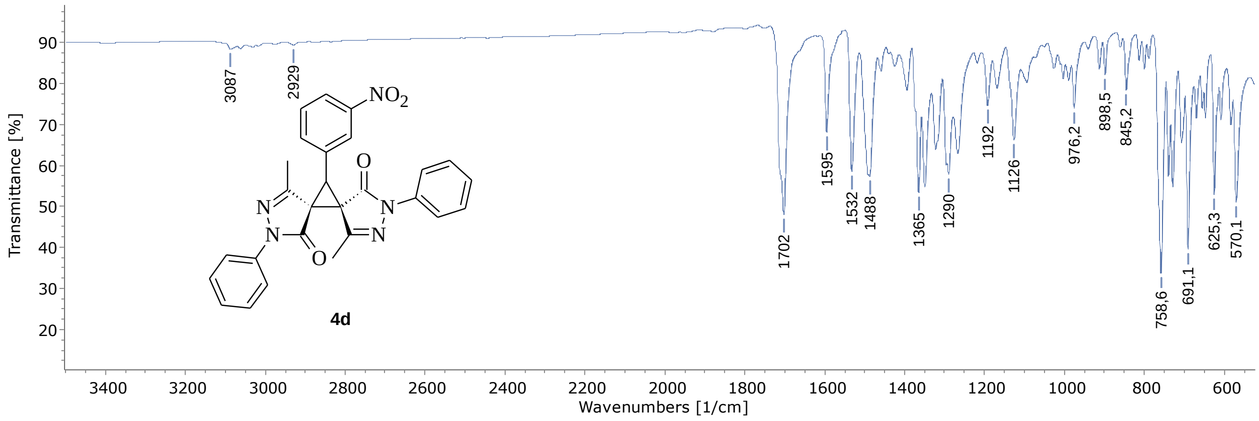

Figure S43. FTIR spectrum of compound **4d**

Acquisition Parameter

Ion Source Type	ESI	Ion Polarity	Positive	Alternating Ion Polarity	off
Mass Range Mode	XtremeScan	Scan Begin	70 m/z	Scan End	400 m/z
Accumulation Time	20000 µs	RF Level	63 %	Trap Drive	54.1
SPS Target Mass	400 m/z	Averages	3 Spectra	n/a	n/a

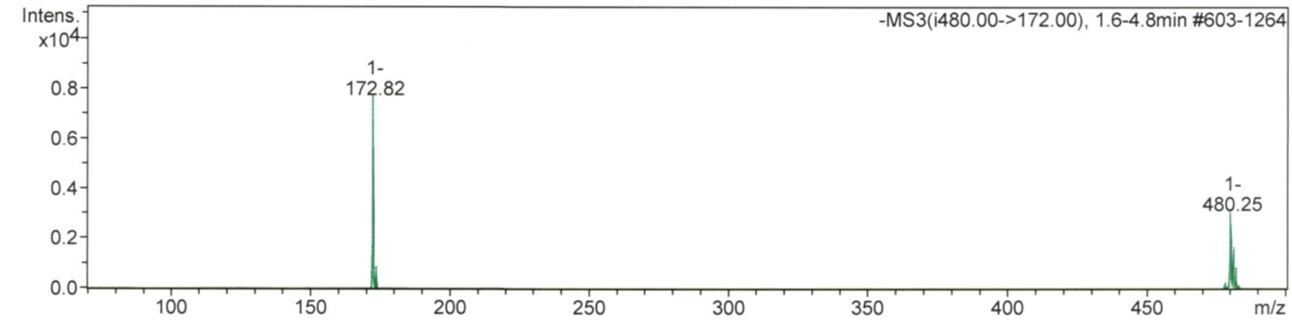

Component	Molecular Mass	Molecule		Absolute Abundance	Relative Abundance
A	172.82	172.90	[M - H]-	7518	100.00
B	480.23	480.77	[M - H]-	2933	39.01

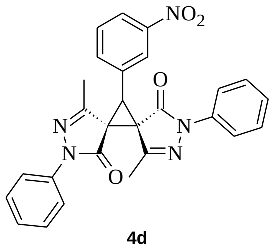

4d

Figure S44. ESI-MS spectrum of compound **4d**

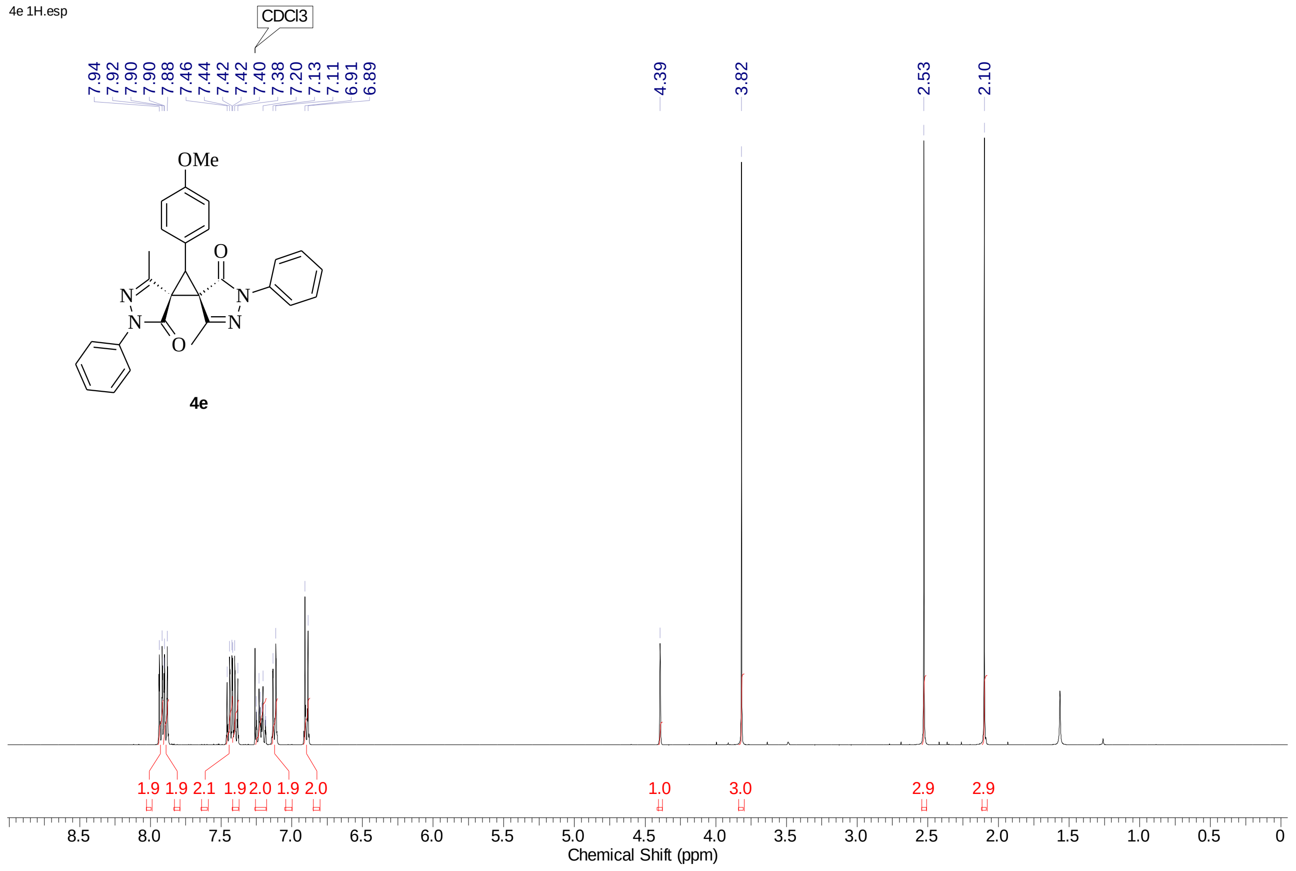

Figure S45. ^{1}H NMR spectrum of compound **4e**

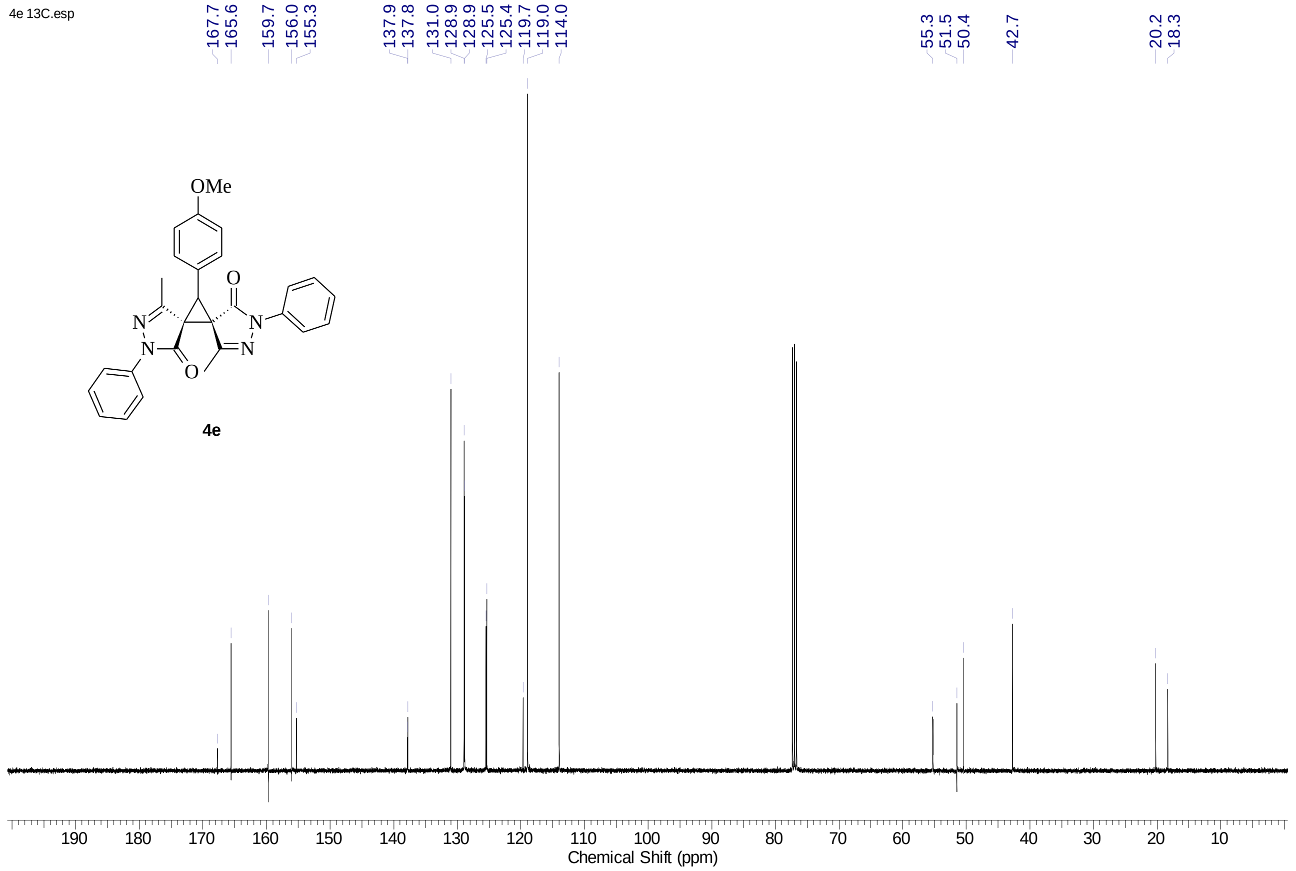

Figure S46. ^{13}C NMR spectrum of compound **4e**

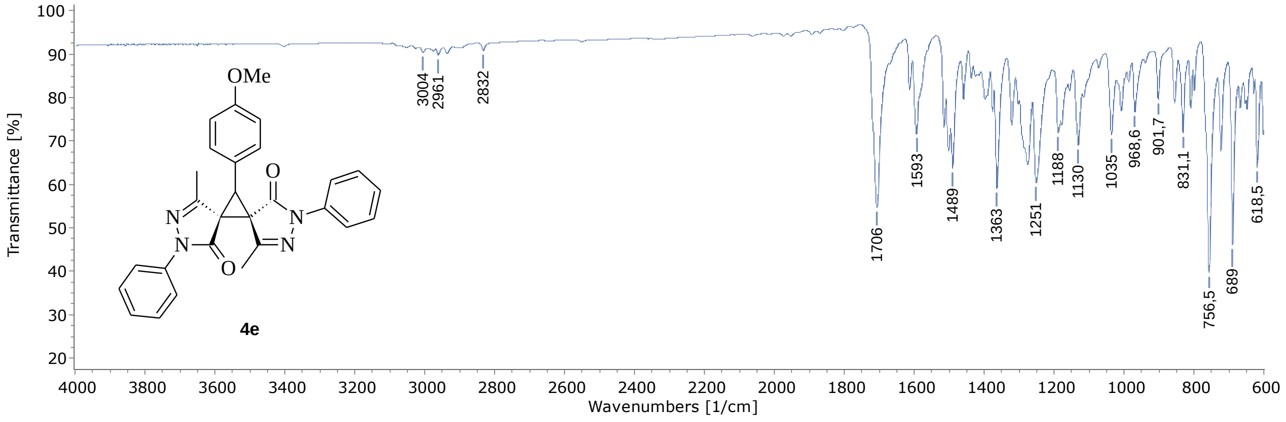

Figure S47. FTIR spectrum of compound **4e**

Acquisition Parameter

Ion Source Type	ESI	Ion Polarity	Positive	Alternating Ion Polarity	off
Mass Range Mode	UltraScan	Scan Begin	70 m/z	Scan End	500 m/z
Accumulation Time	199196 µs	RF Level	63 %	Trap Drive	54.1
SPS Target Mass	400 m/z	Averages	5 Spectra	n/a	n/a

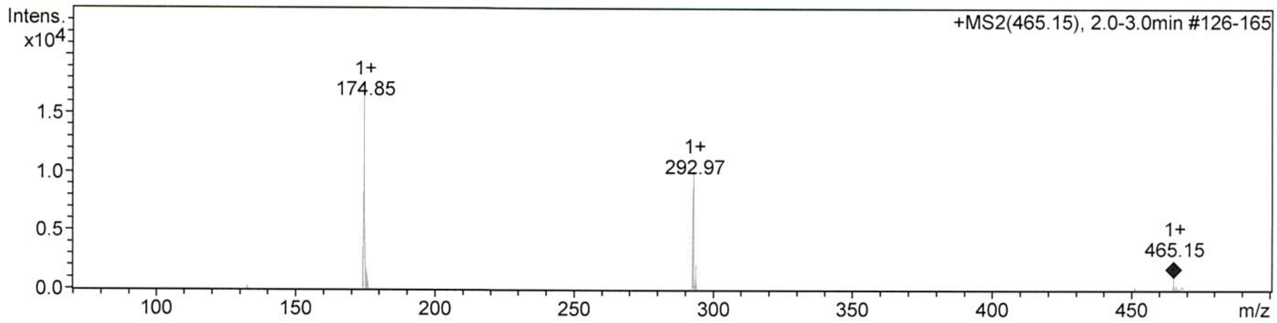

Component	Molecular Mass	Molecule	Absolute Abundance	Relative Abundance
A	174.85	174.92 [M + H]+	16050	100.00
B	292.97	293.12 [M + H]+	9561	59.57
C	465.15	465.53 [M + H]+	1069	6.66

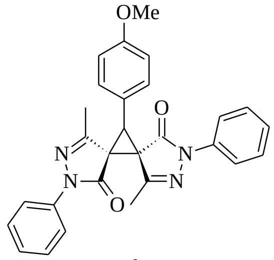

4e

Figure S48. ESI-MS spectrum of compound **4e**

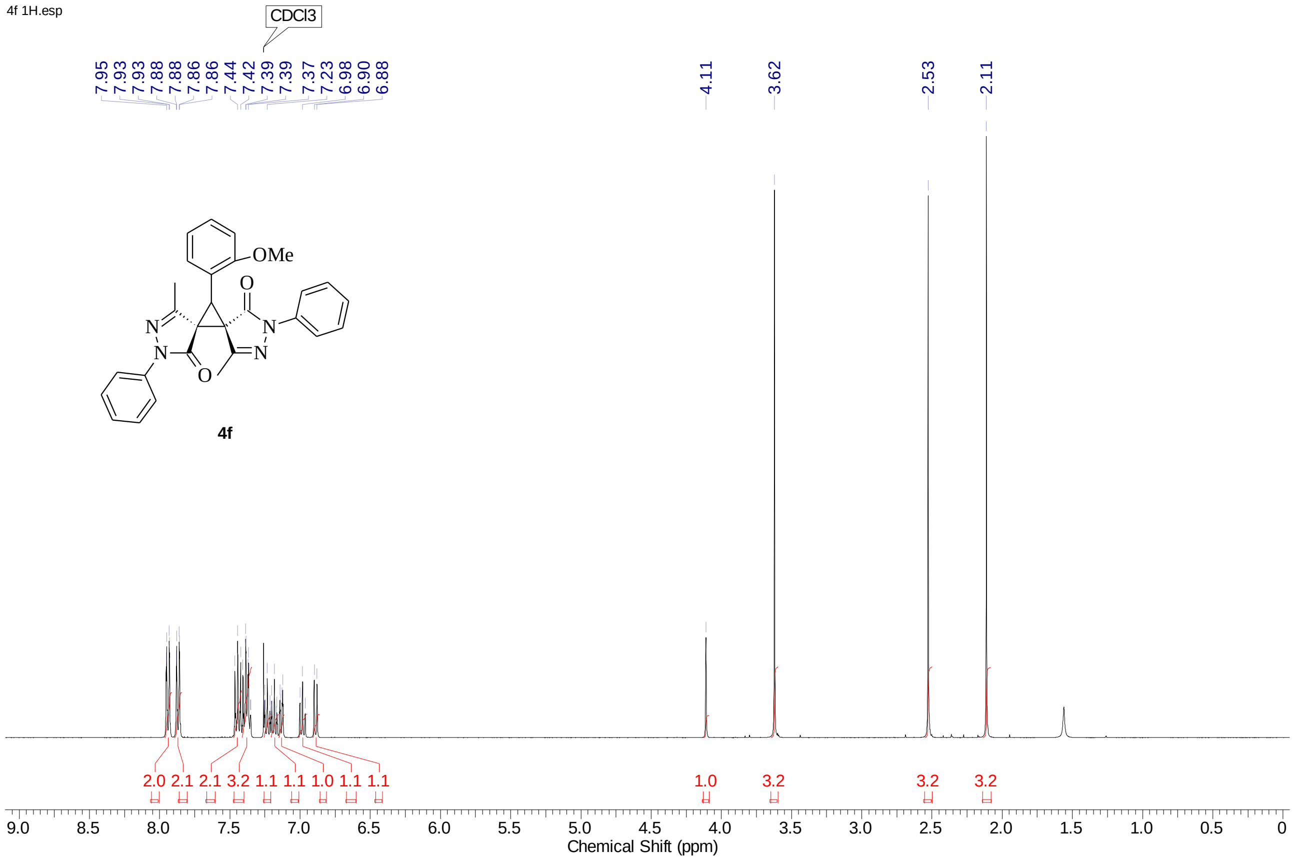

Figure S49. ^{1}H NMR spectrum of compound **4f**

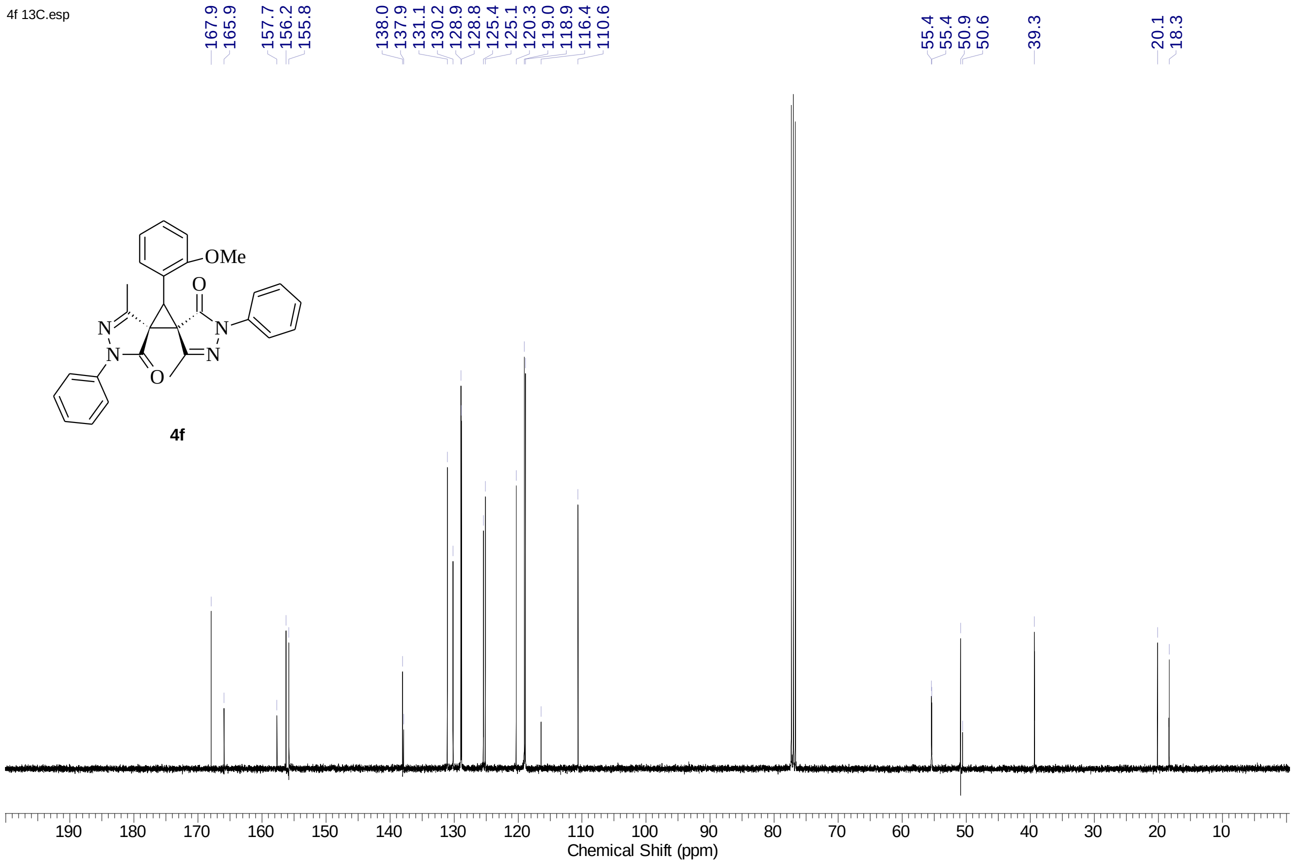

Figure S50. ^{13}C NMR spectrum of compound **4f**

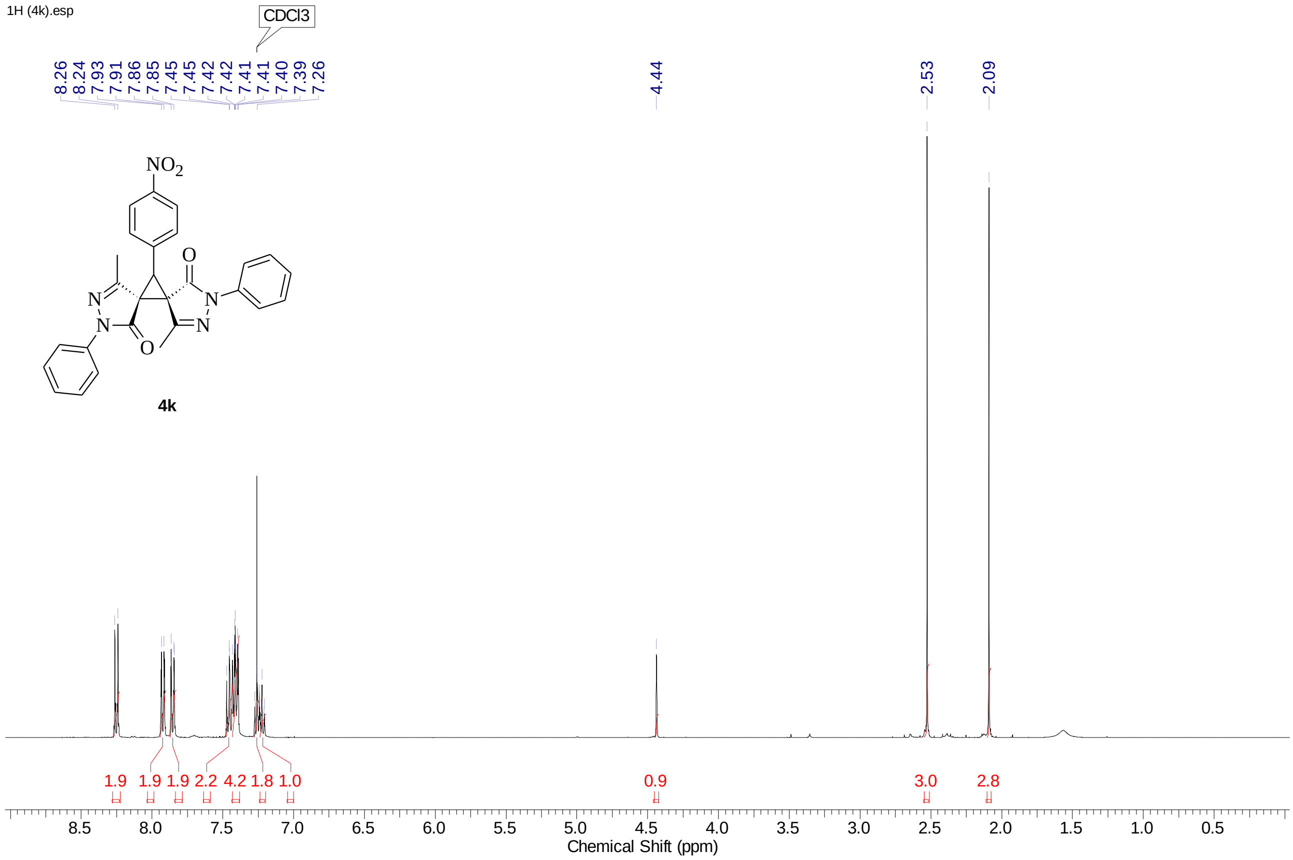

Figure S51. ^{1}H NMR spectrum of compound **4k**

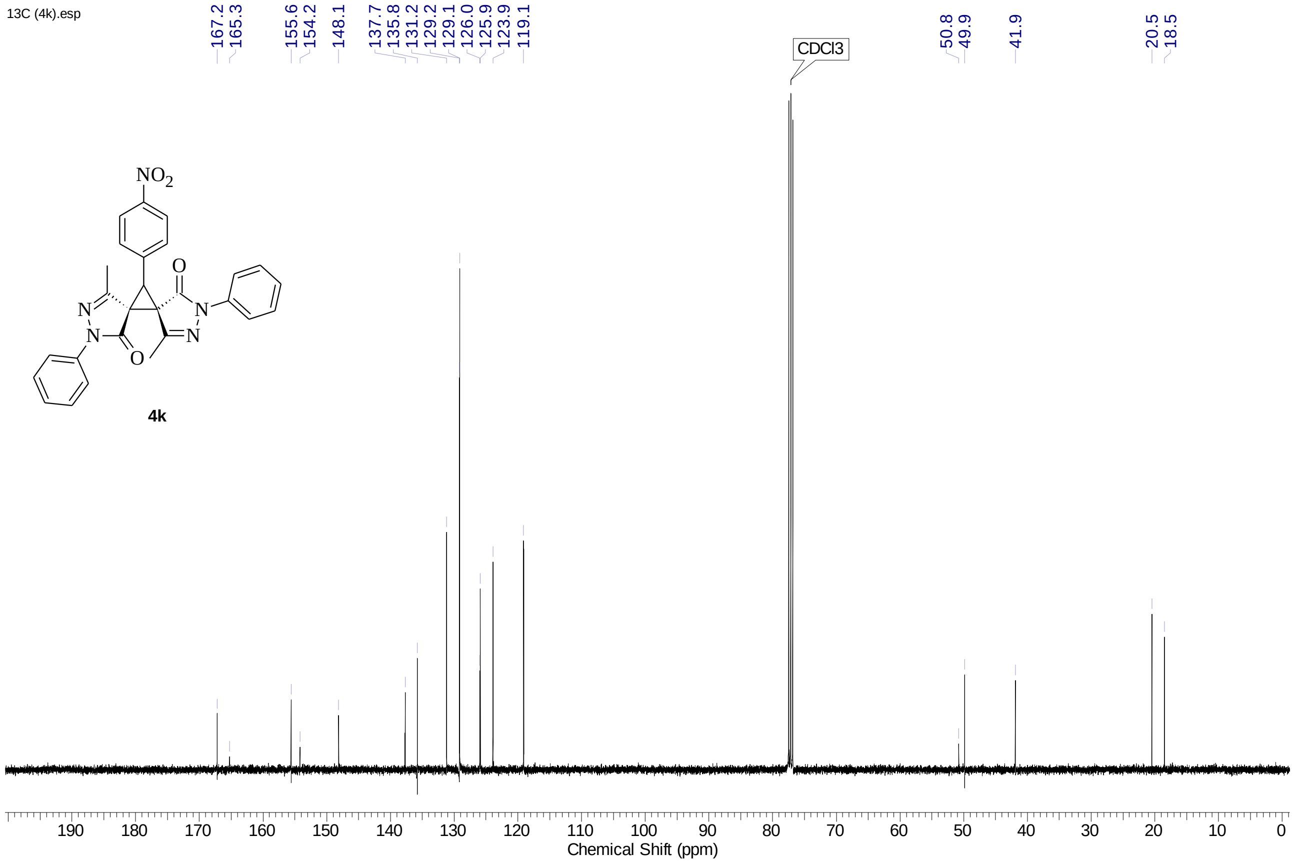

Figure S52. ^{13}C NMR spectrum of compound **4k**

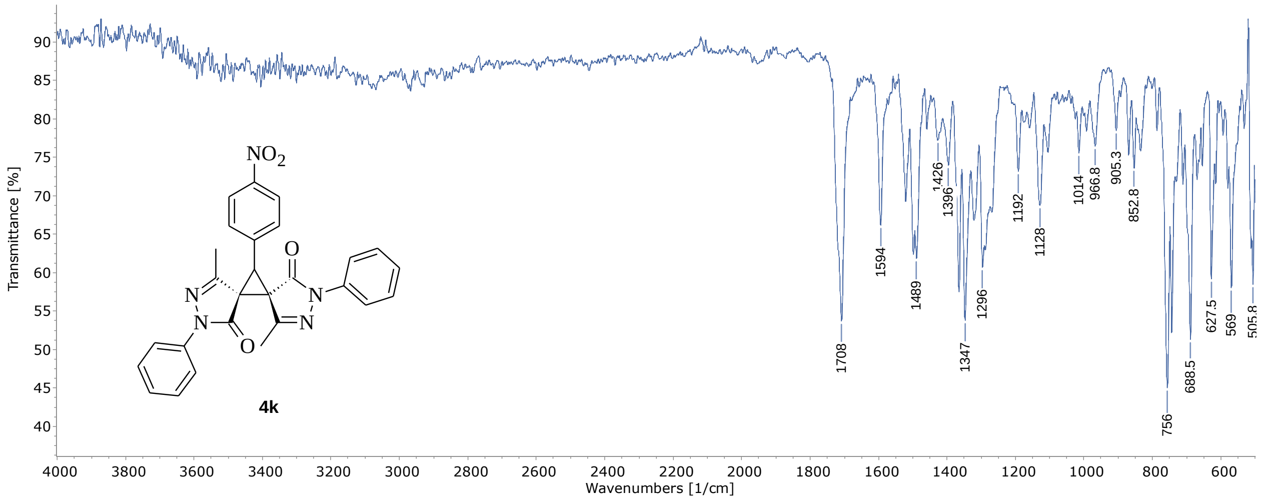

Figure S53. FTIR spectrum of compound **4k**

Acquisition Parameter

Ion Source Type	ESI	Ion Polarity	Positive	Alternating Ion Polarity	off
Mass Range Mode	UltraScan	Scan Begin	70 m/z	Scan End	600 m/z
Accumulation Time	200000 µs	RF Level	63 %	Trap Drive	54.1
SPS Target Mass	400 m/z	Averages	5 Spectra	n/a	n/a

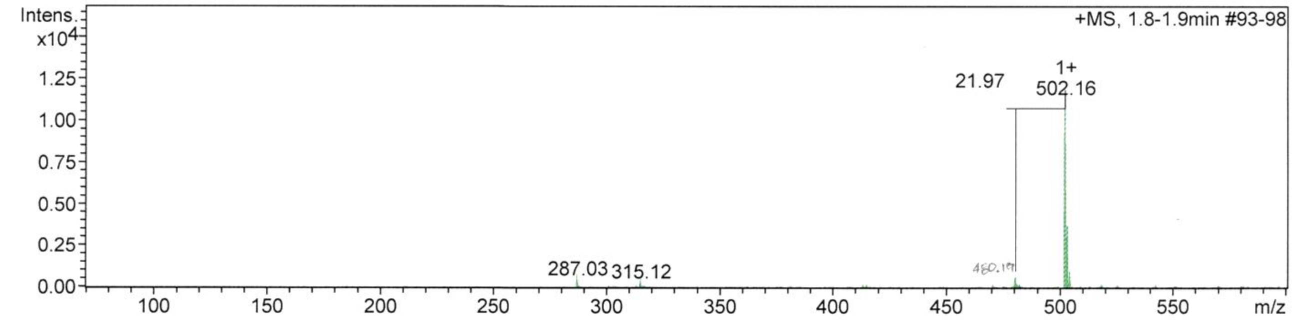

Component	Molecular Mass	Molecule		Absolute Abundance	Relative Abundance
A	502.16	502.49	[M + H]+	11262	100.00

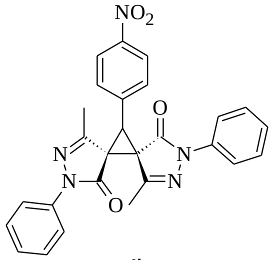

4k

Figure S54. ESI-MS spectrum of compound **4k**

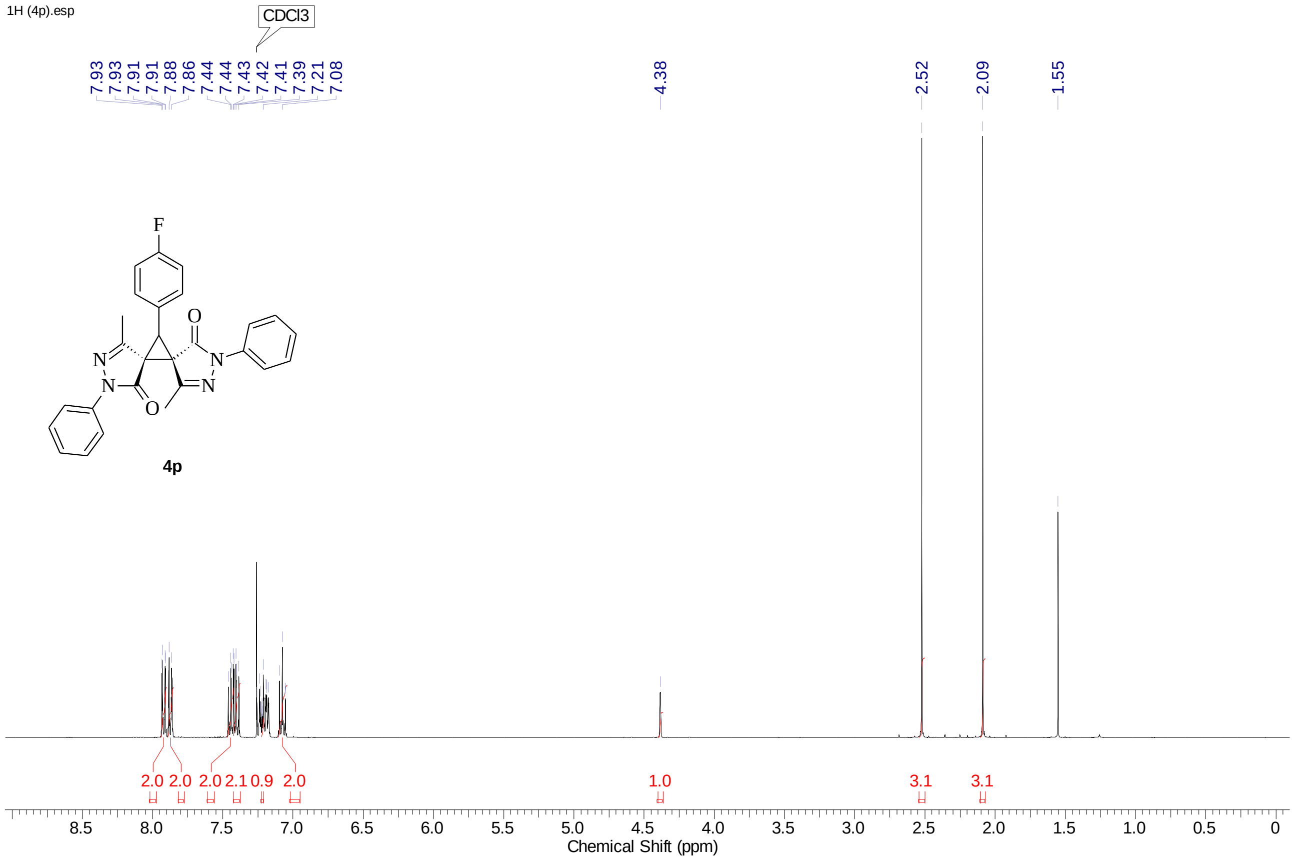

Figure S55. ^{1}H NMR spectrum of compound **4p**

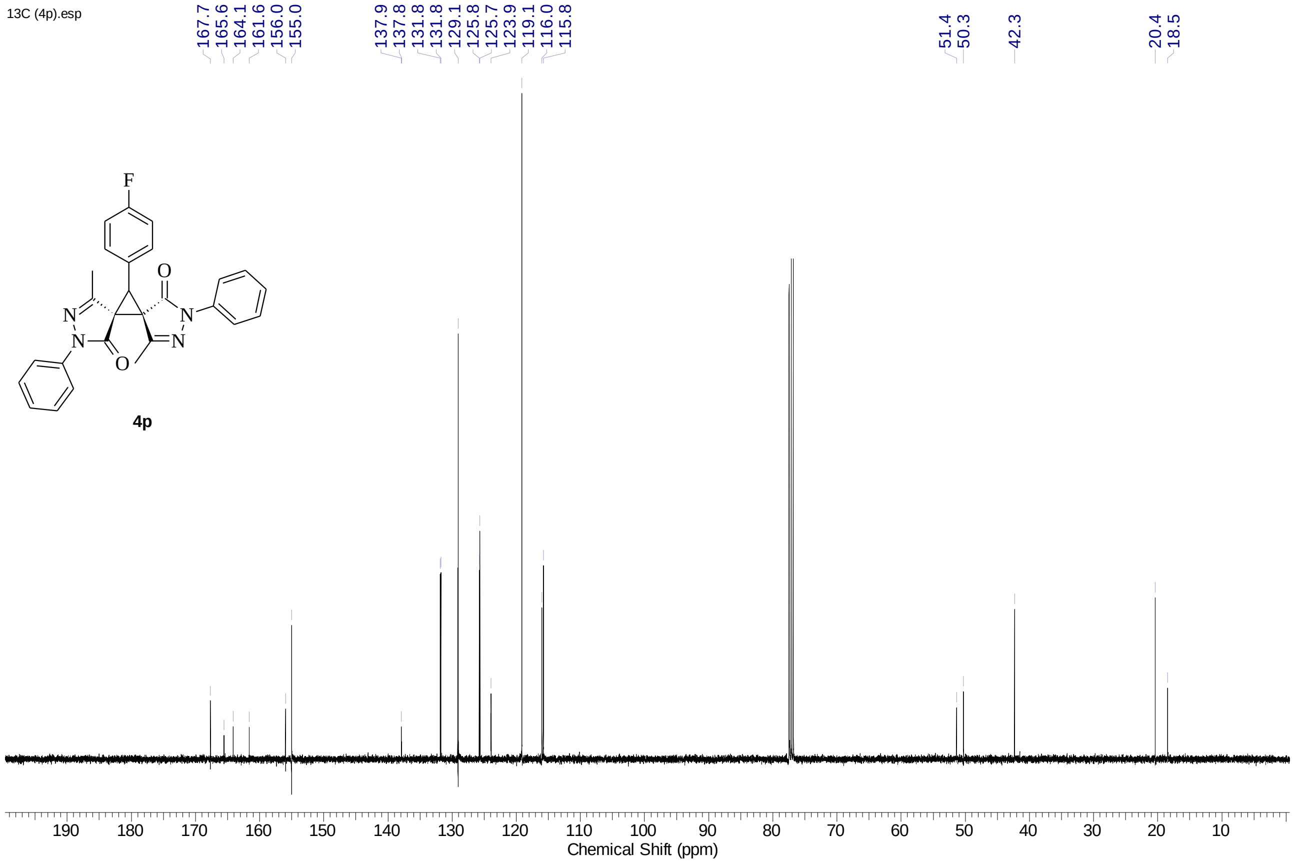

Figure S56. ^{13}C NMR spectrum of compound **4p**

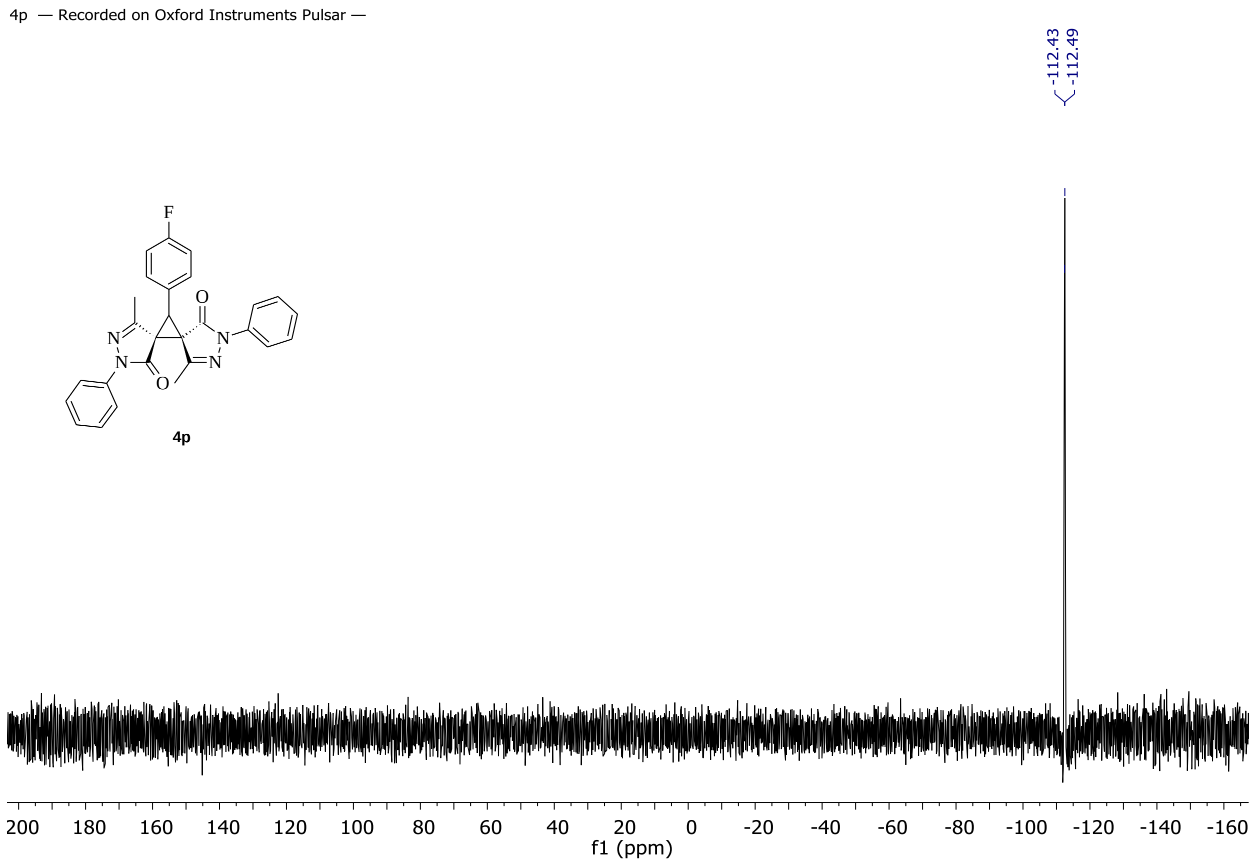

Figure S57. ^{19}F NMR spectrum of compound **4p**

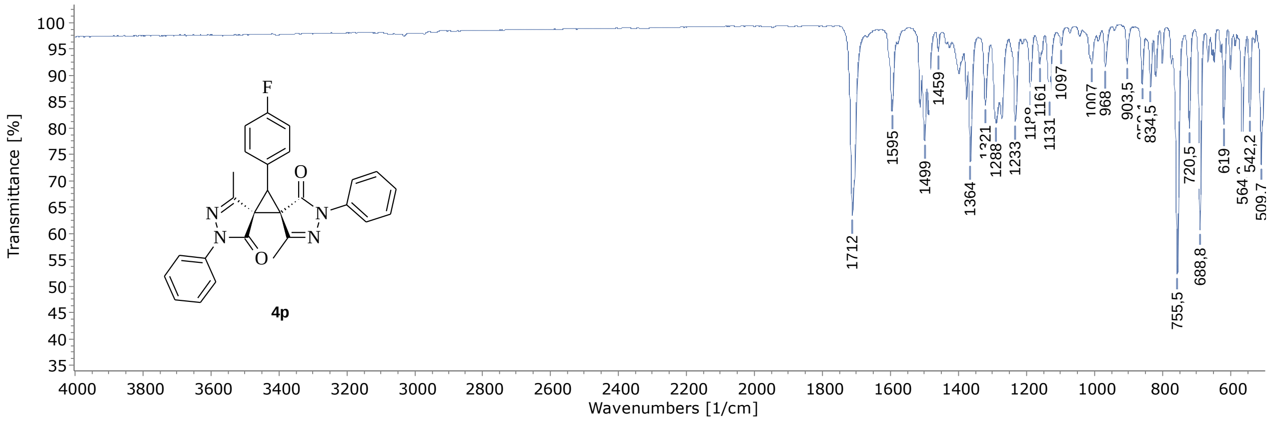

Figure S58. FTIR spectrum of compound **4p**

Acquisition Parameter

Ion Source Type	ESI	Ion Polarity	Positive	Alternating Ion Polarity	off
Mass Range Mode	UltraScan	Scan Begin	70 m/z	Scan End	600 m/z
Accumulation Time	9580 µs	RF Level	63 %	Trap Drive	54.1
SPS Target Mass	400 m/z	Averages	5 Spectra	n/a	n/a

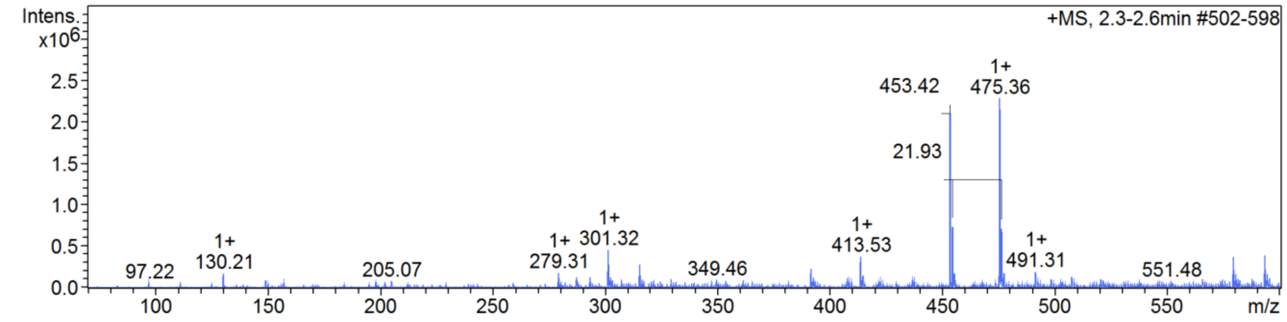

Component	Molecular Mass	Molecule		Absolute Abundance	Relative Abundance
A	475.35	475.78	[M + H]+	2274889	100.00
B	453.40	453.81	[M + H]+	2093773	92.04
C	301.33	302.01	[M + H]+	455324	20.02

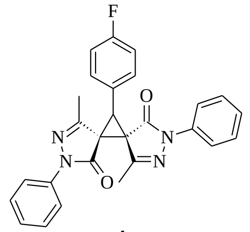

4p

Figure S59. ESI-MS spectrum of compound **4p**

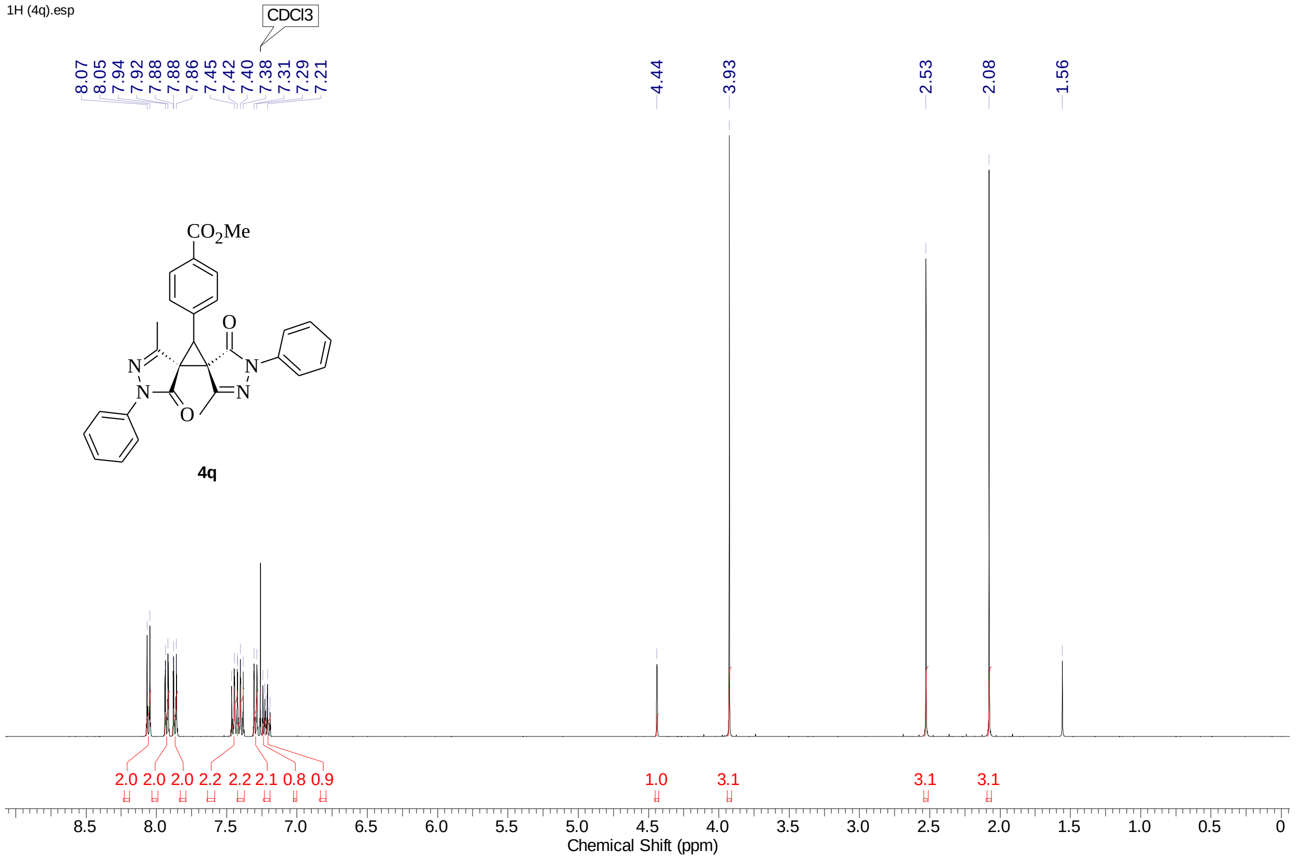

Figure S60. ^{1}H NMR spectrum of compound **4q**

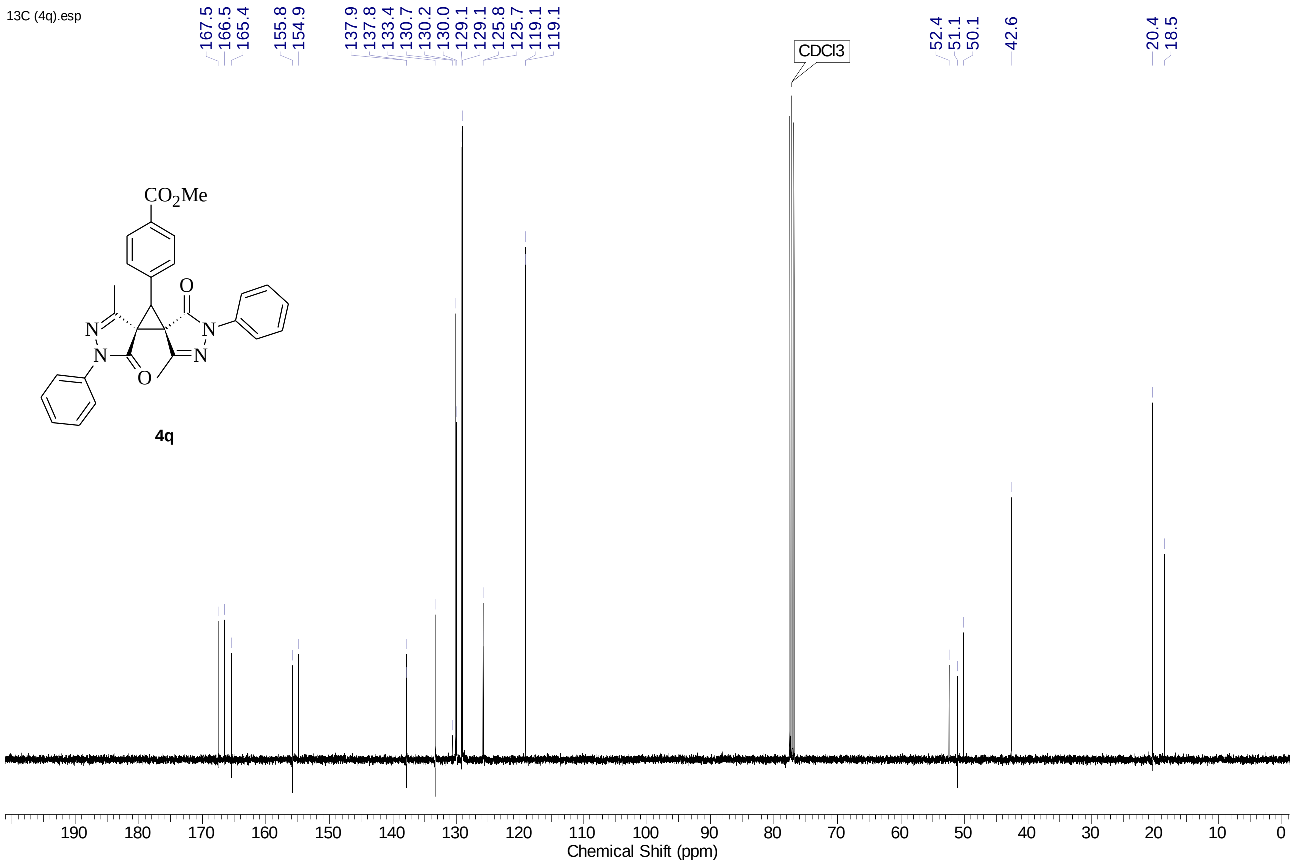

Figure S61. ^{13}C NMR spectrum of compound **4q**

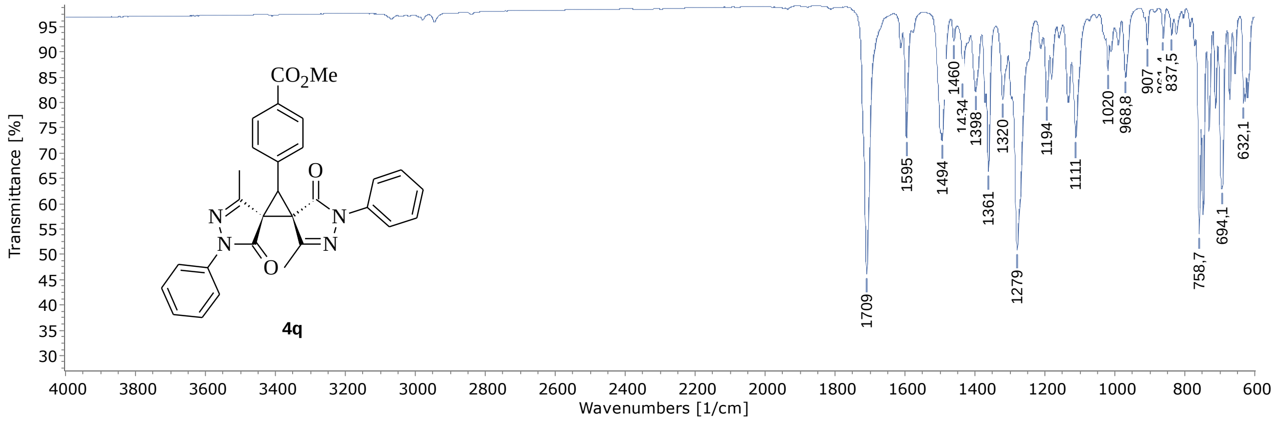

Figure S62. FTIR spectrum of compound **4q**

Acquisition Parameter

Ion Source Type	ESI	Ion Polarity	Positive	Alternating Ion Polarity	off
Mass Range Mode	UltraScan	Scan Begin	70 m/z	Scan End	600 m/z
Accumulation Time	6959 µs	RF Level	63 %	Trap Drive	54.1
SPS Target Mass	400 m/z	Averages	5 Spectra	n/a	n/a

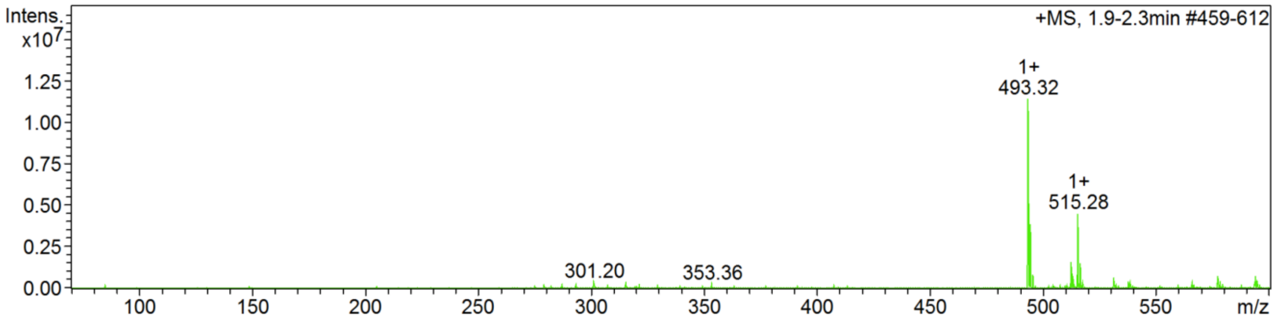

Component	Molecular Mass	Molecule		Absolute Abundance	Relative Abundance
A	493.30	493.68	[M + H]+	11388026	100.00
B	515.27	515.66	[M + H]+	4469086	39.24
C	1023.56	1024.28	[M + H]+	1620959	14.23
D	593.71	594.37	[M + H]+	771212	6.77
E	577.26	578.08	[M + H]+	758822	6.66

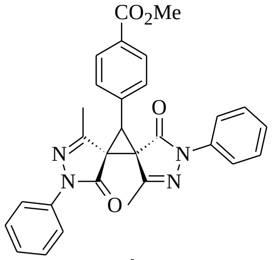

4q

Figure S63. ESI-MS spectrum of compound **4q**

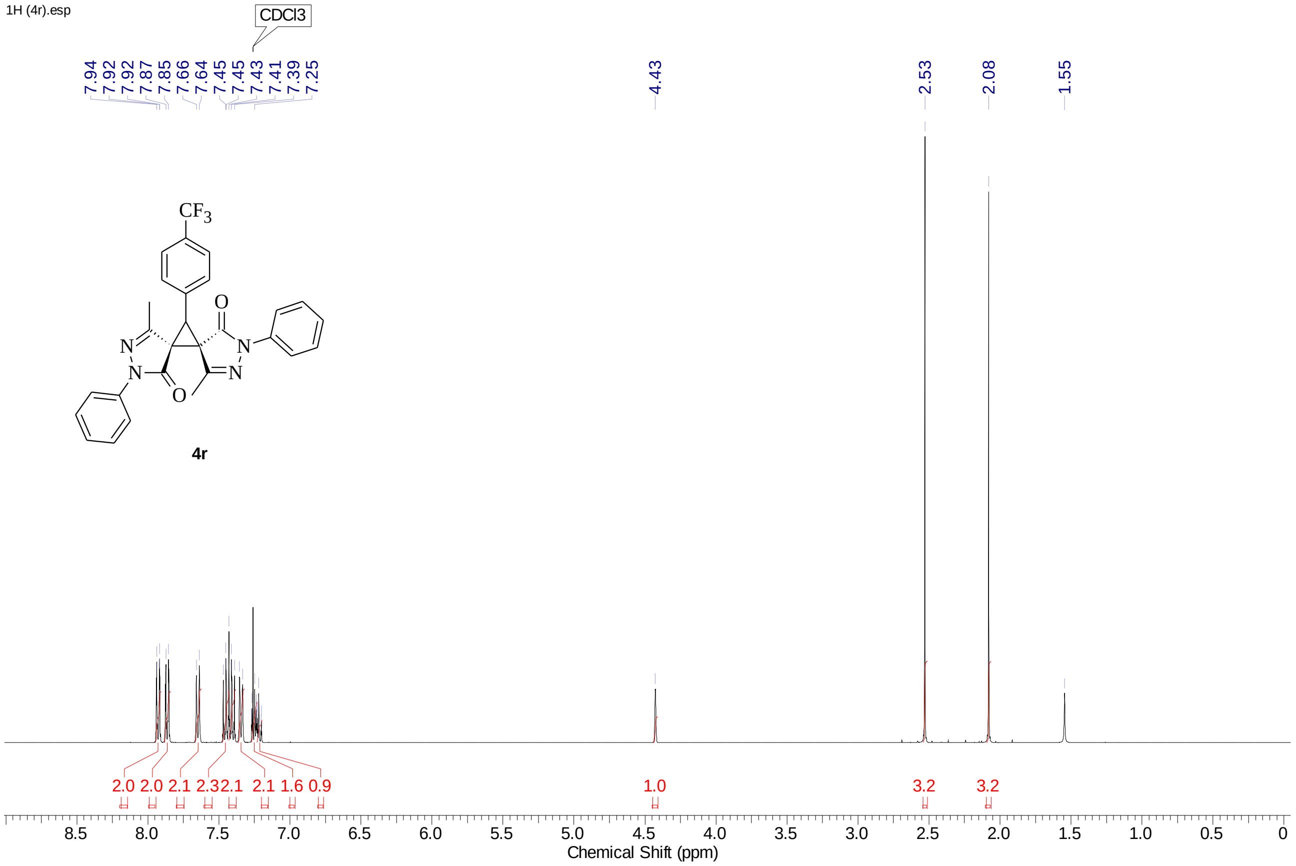

Figure S64. ^{1}H NMR spectrum of compound **4r**

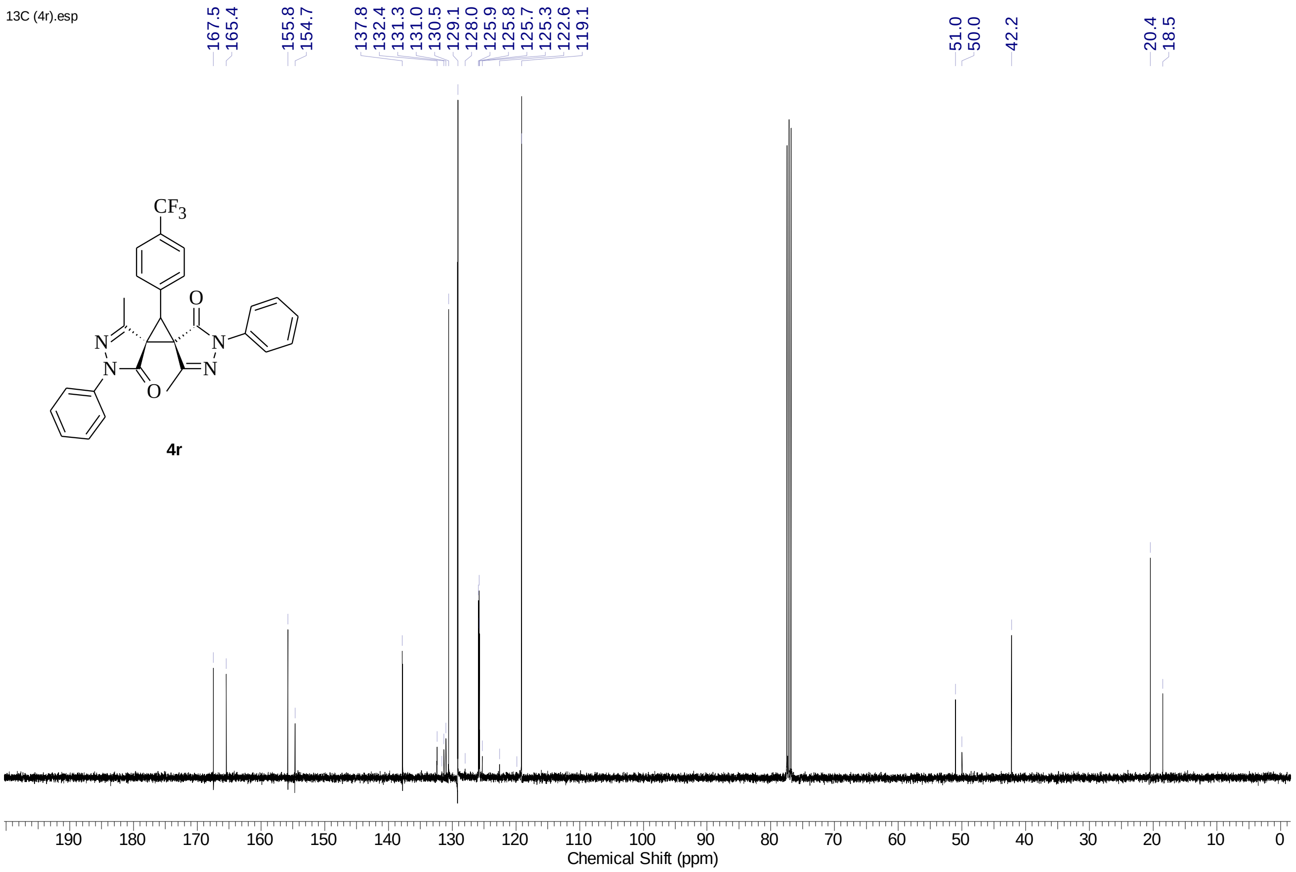

Figure S65. ^{13}C NMR spectrum of compound **4r**

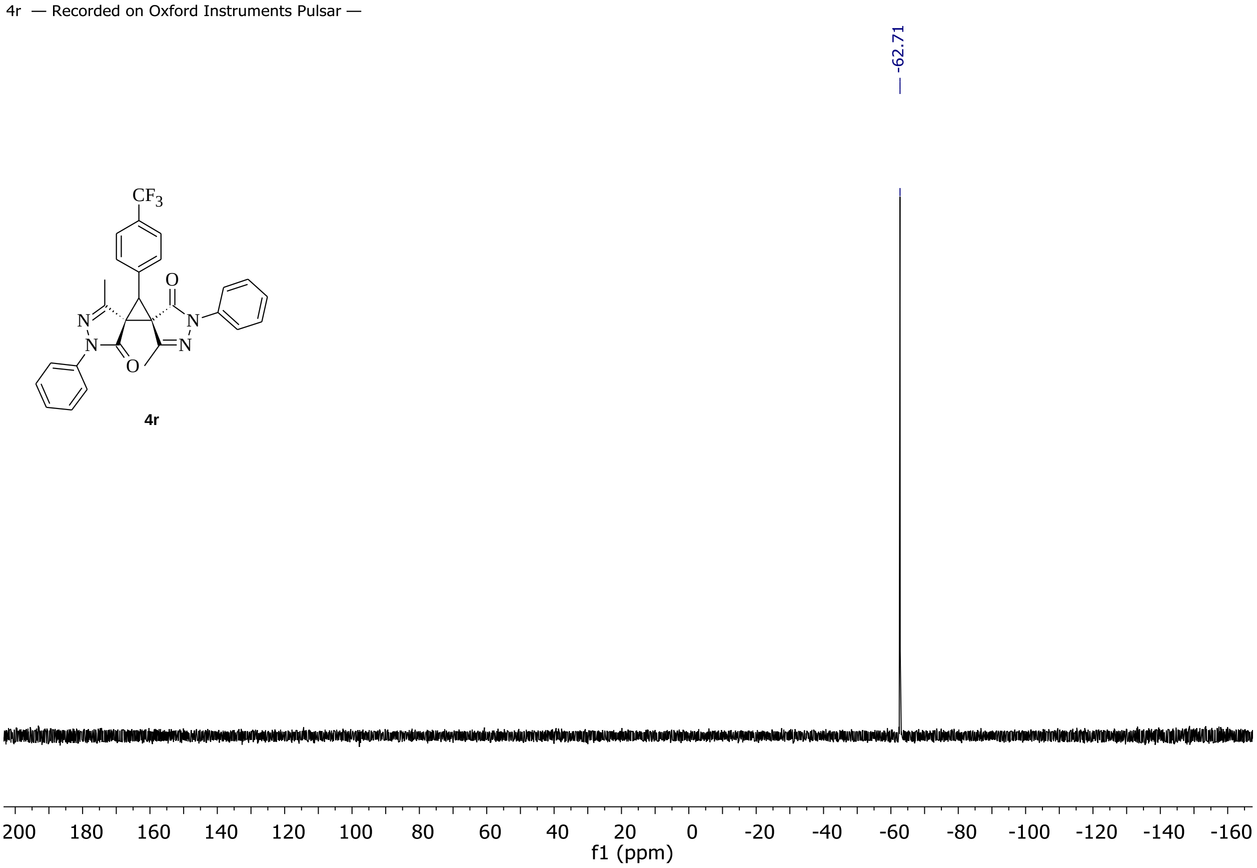

Figure S66. ^{19}F NMR spectrum of compound **4r**

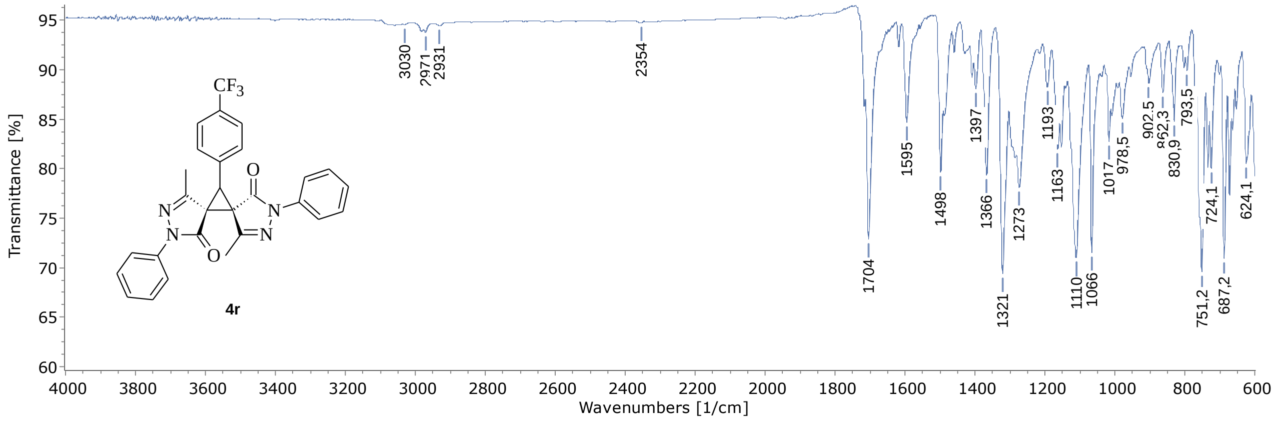

Figure S67. FTIR spectrum of compound **4r**

Acquisition Parameter

Ion Source Type	ESI	Ion Polarity	Positive	Alternating Ion Polarity	off
Mass Range Mode	UltraScan	Scan Begin	70 m/z	Scan End	600 m/z
Accumulation Time	13368 µs	RF Level	63 %	Trap Drive	54.1
SPS Target Mass	400 m/z	Averages	5 Spectra	n/a	n/a

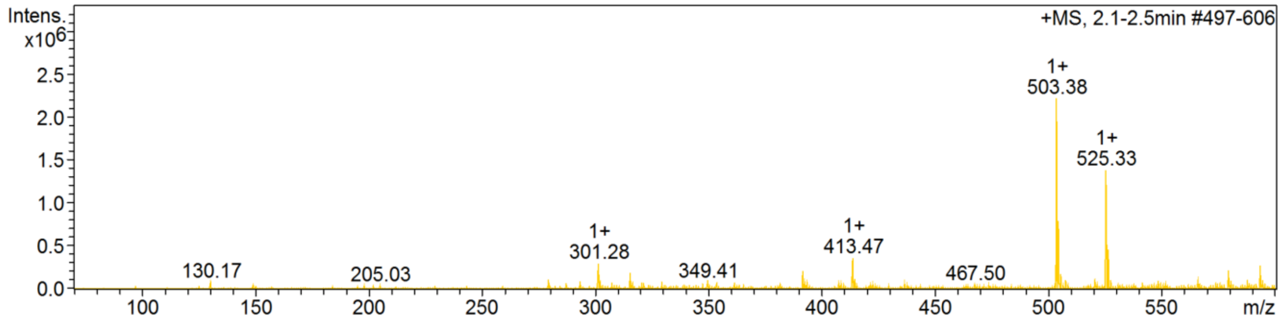

Component	Molecular Mass	Molecule		Absolute Abundance	Relative Abundance
A	503.36	503.75	[M + H]+	2206733	100.00
B	525.32	525.72	[M + H]+	1378402	62.46
C	413.47	413.97	[M + H]+	356961	16.18
D	301.28	301.90	[M + H]+	294262	13.33

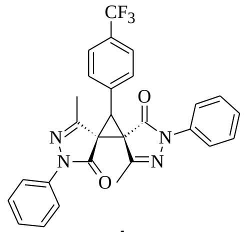

4r

Figure S68. ESI-MS spectrum of compound **4r**

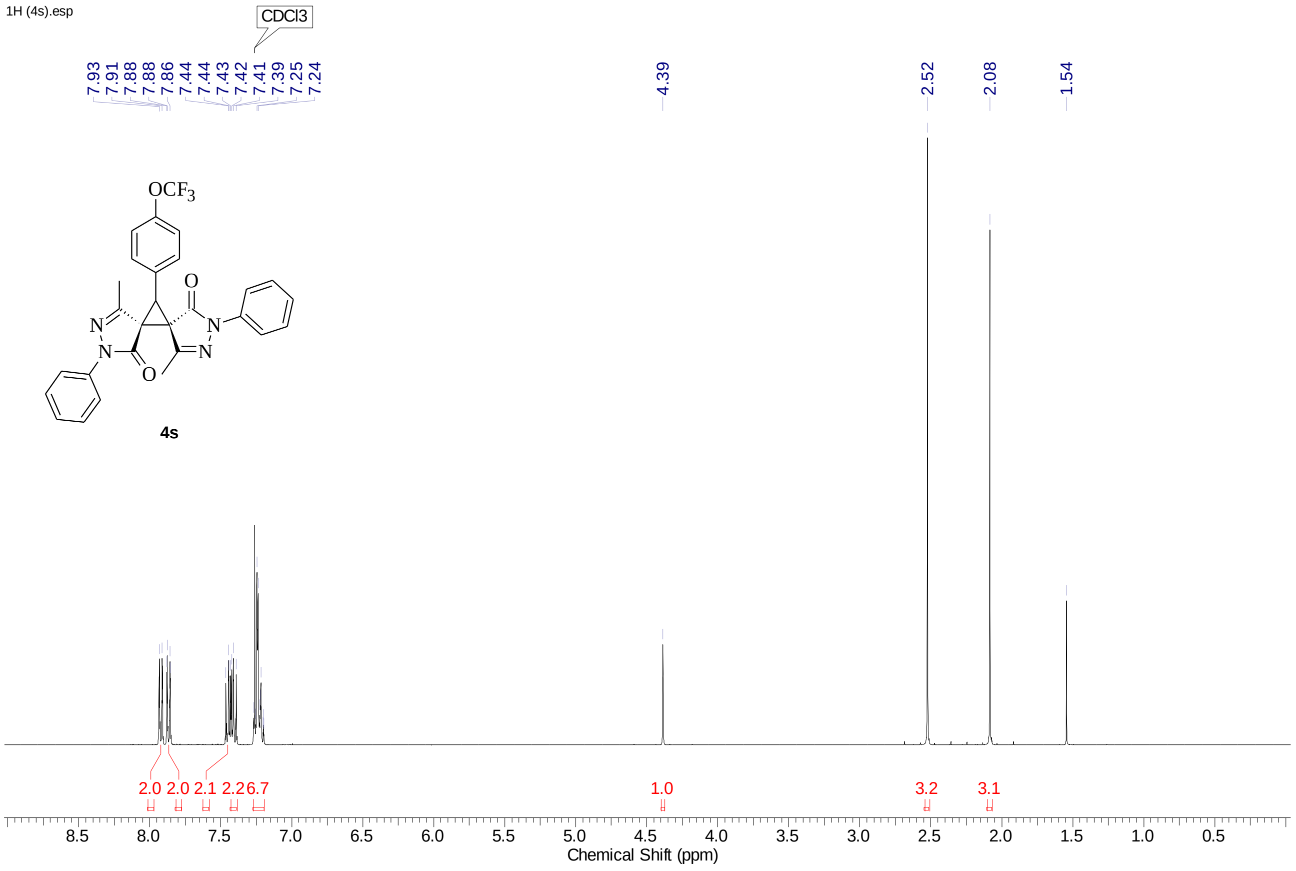

Figure S69. ^{1}H NMR spectrum of compound **4s**

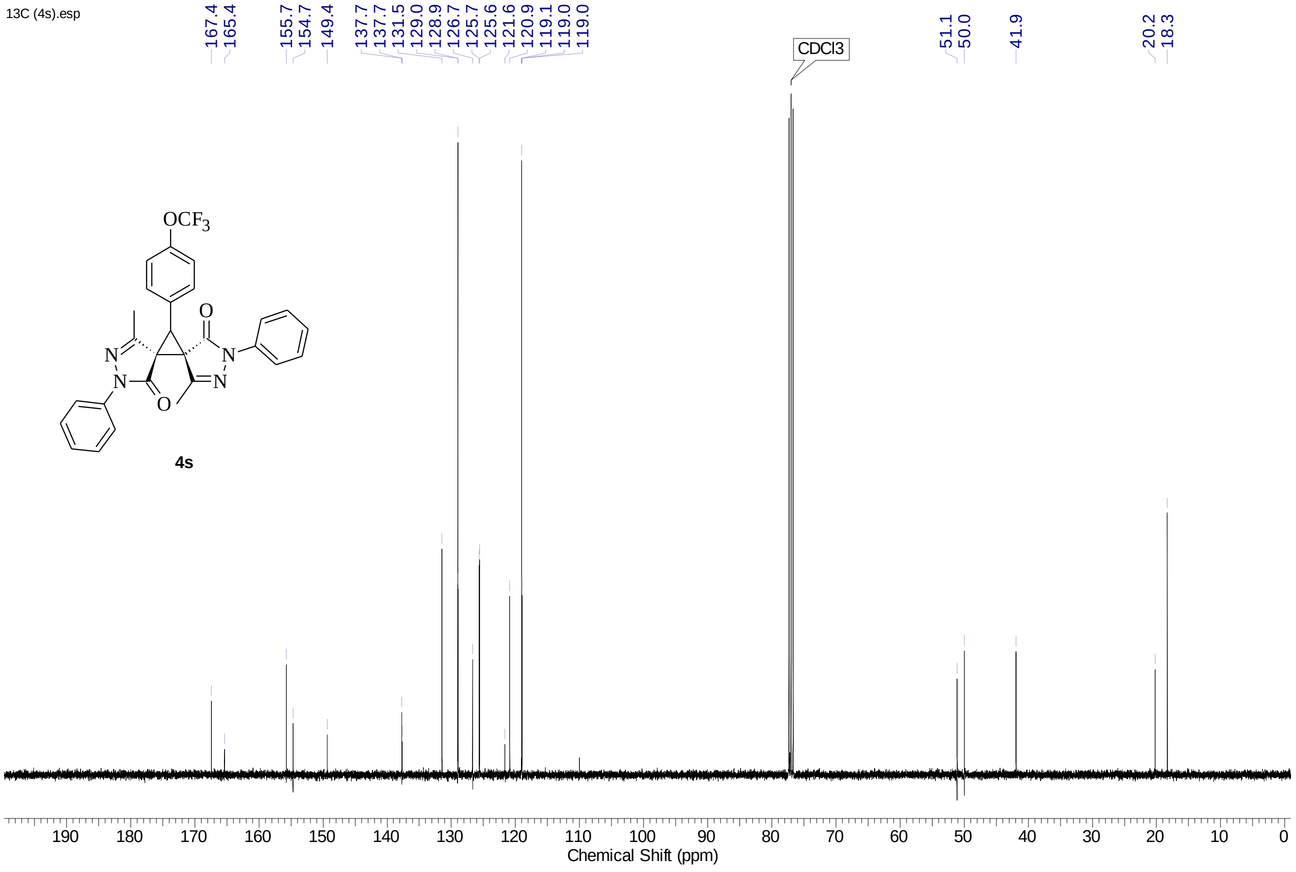

Figure S70. ^{13}C NMR spectrum of compound **4s**

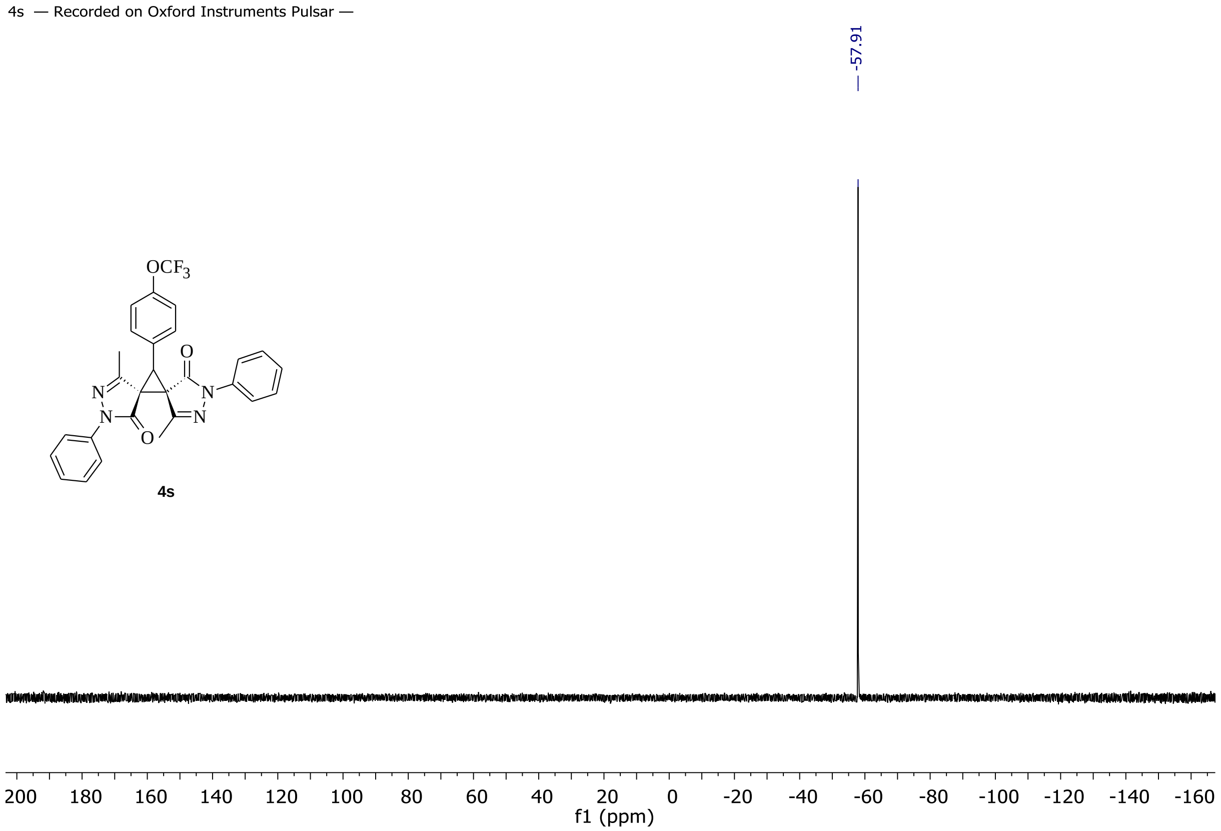

Figure S71. ^{19}F NMR spectrum of compound **4s**

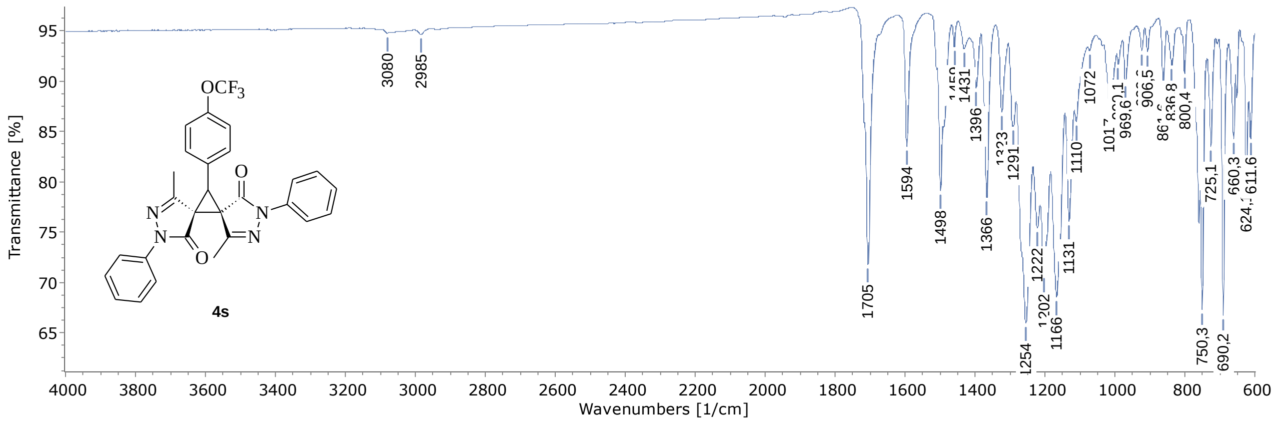

Figure S72. FTIR spectrum of compound **4s**

Acquisition Parameter

Ion Source Type	ESI	Ion Polarity	Positive	Alternating Ion Polarity	off
Mass Range Mode	UltraScan	Scan Begin	70 m/z	Scan End	600 m/z
Accumulation Time	9765 µs	RF Level	63 %	Trap Drive	54.1
SPS Target Mass	400 m/z	Averages	5 Spectra	n/a	n/a

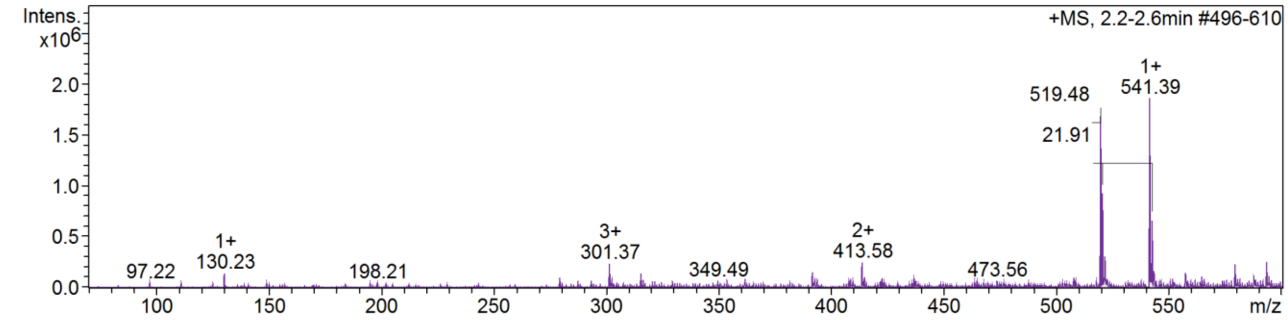

Component	Molecular Mass	Molecule		Absolute Abundance	Relative Abundance
A	541.38	541.86	[M + H]+	1850876	100.00
B	519.46	520.10	[M + H]+	1678022	90.66

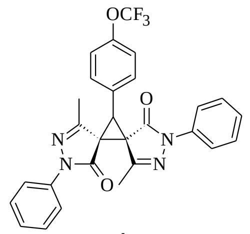

4s

Figure S73. ESI-MS spectrum of compound **4s**

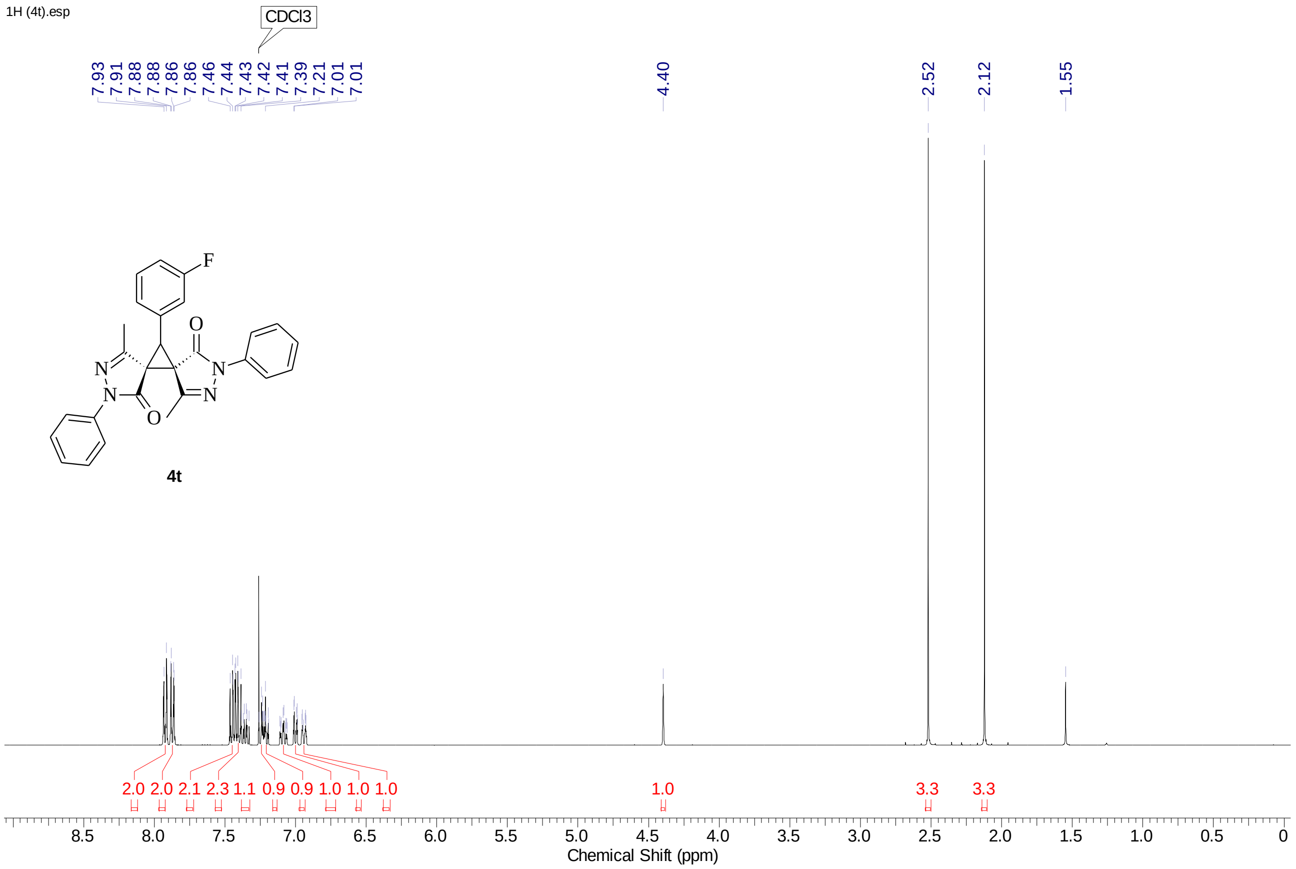

Figure S74. ^{1}H NMR spectrum of compound **4t**

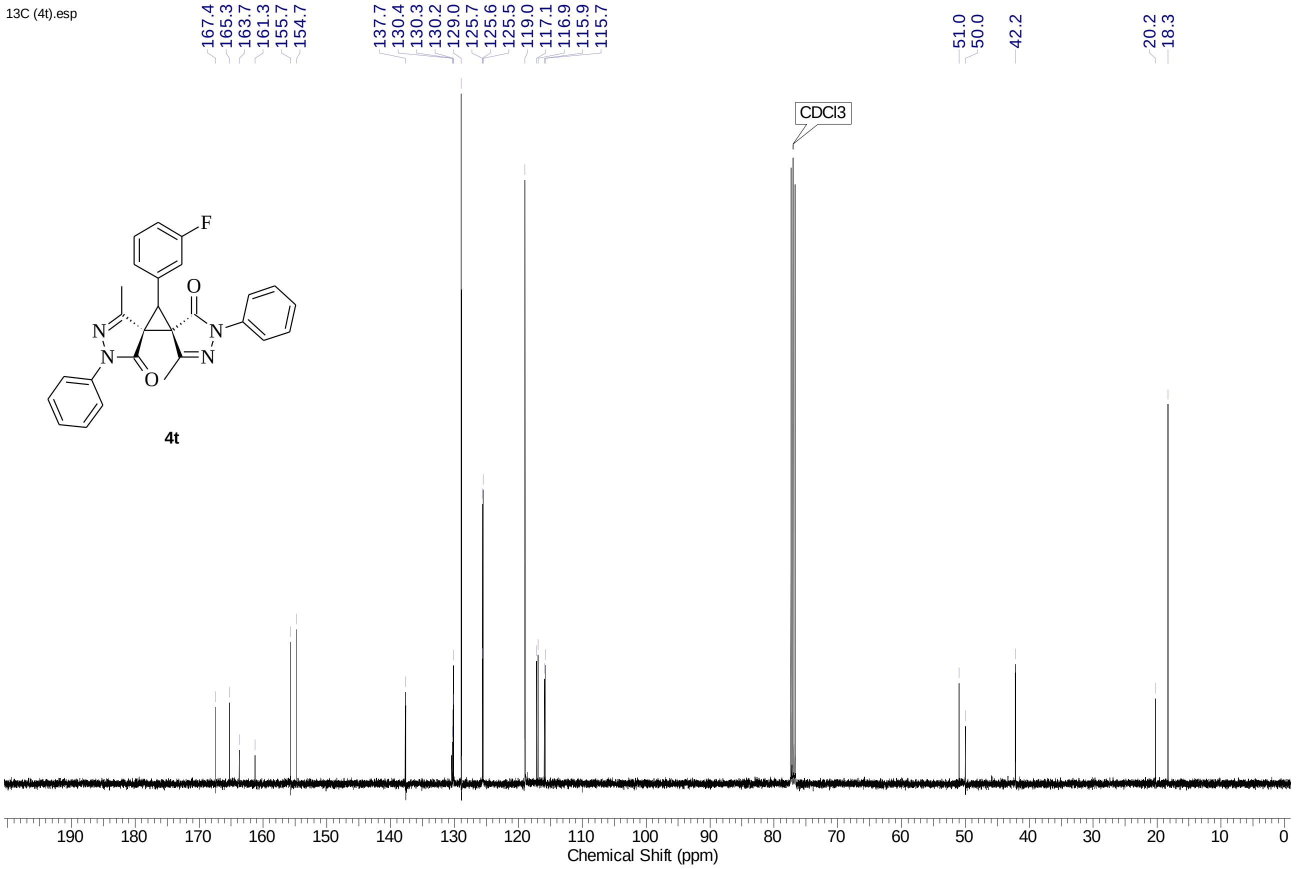

Figure S75. ^{13}C NMR spectrum of compound **4t**

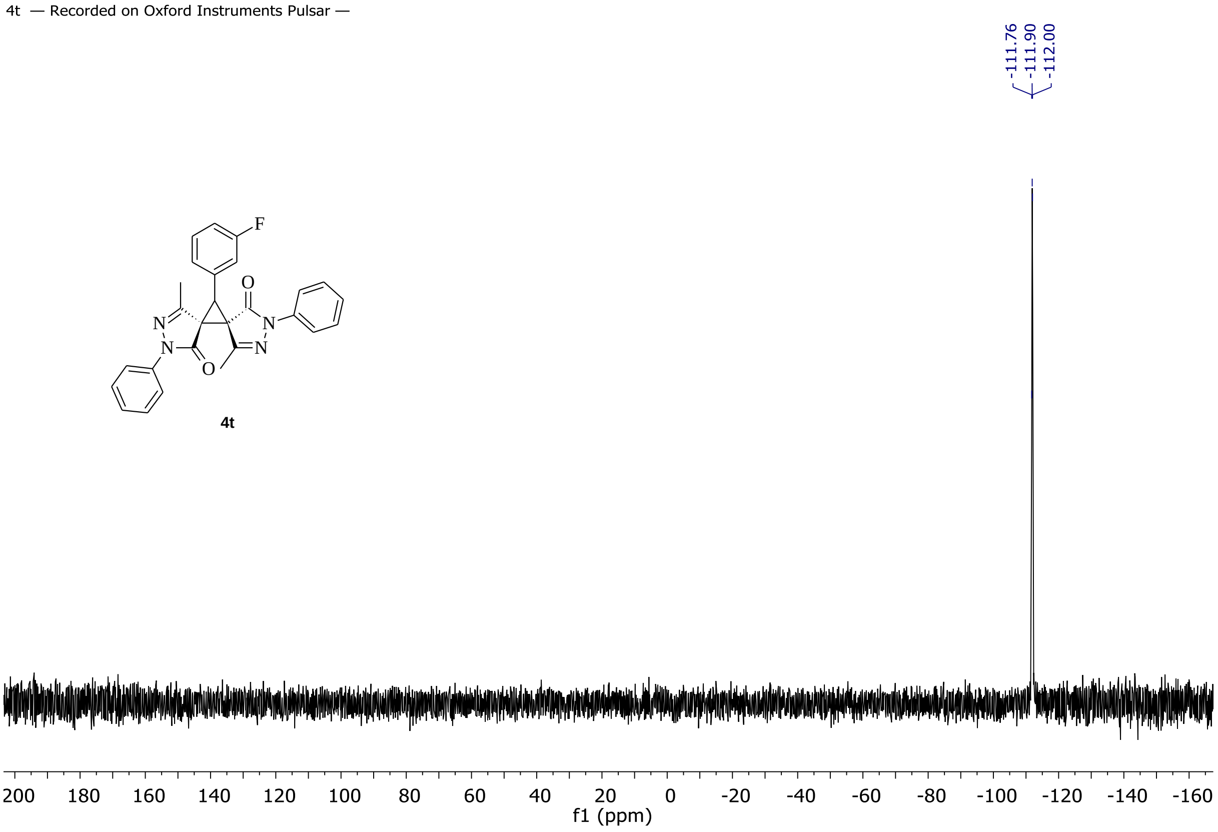

Figure S76. ^{19}F NMR spectrum of compound **4t**

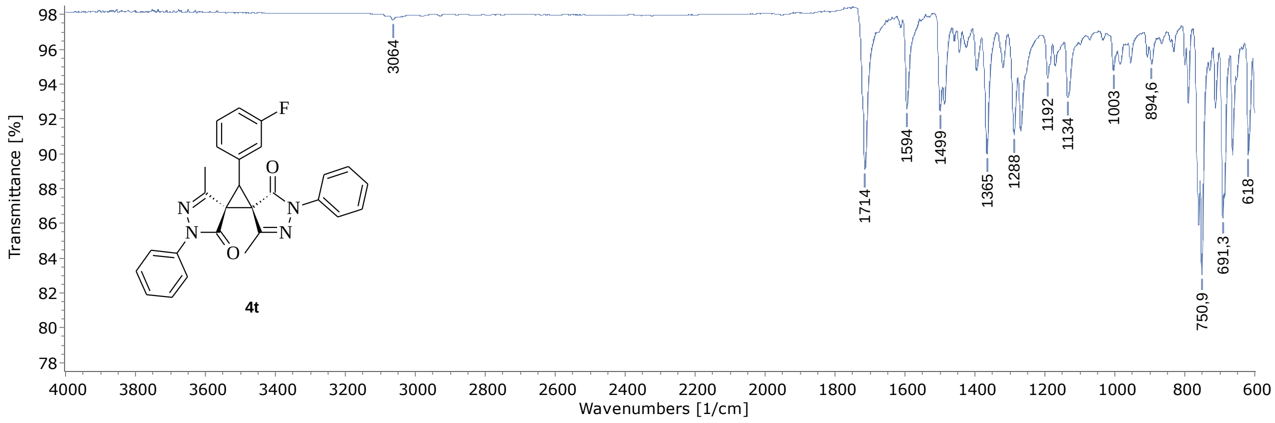

Figure S77. FTIR spectrum of compound **4t**

Acquisition Parameter

Ion Source Type	ESI	Ion Polarity	Positive	Alternating Ion Polarity	off		
Mass Range Mode	UltraScan	Scan Begin	70 m/z	Scan End	600 m/z		
Accumulation Time	200000 µs	RF Level	63 %	Trap Drive	54.1		
SPS Target Mass	400 m/z	Averages	5 Spectra	n/a	n/a		

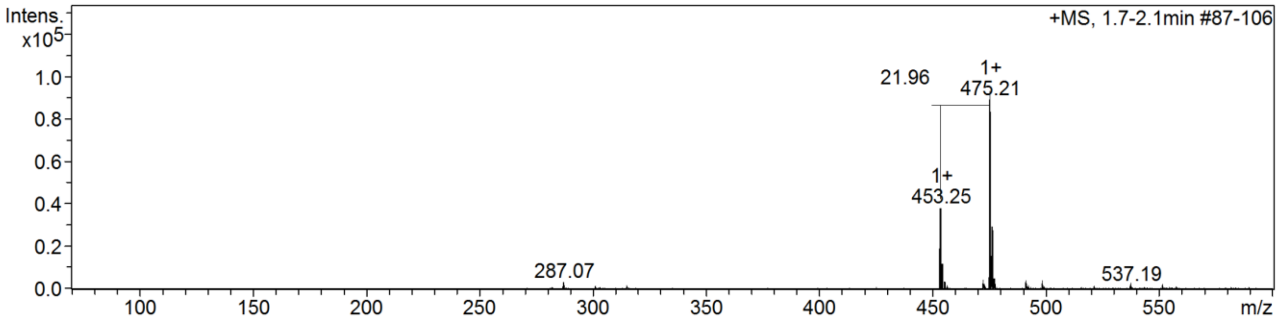

Component	Molecular Mass	Molecule	Absolute Abundance	Relative Abundance
A	475.19	475.53 [M + H]+	88901	100.00
B	453.24	453.65 [M + H]+	37933	42.67

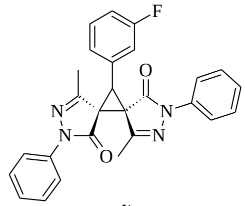

4t

Figure S78. ESI-MS spectrum of compound **4t**

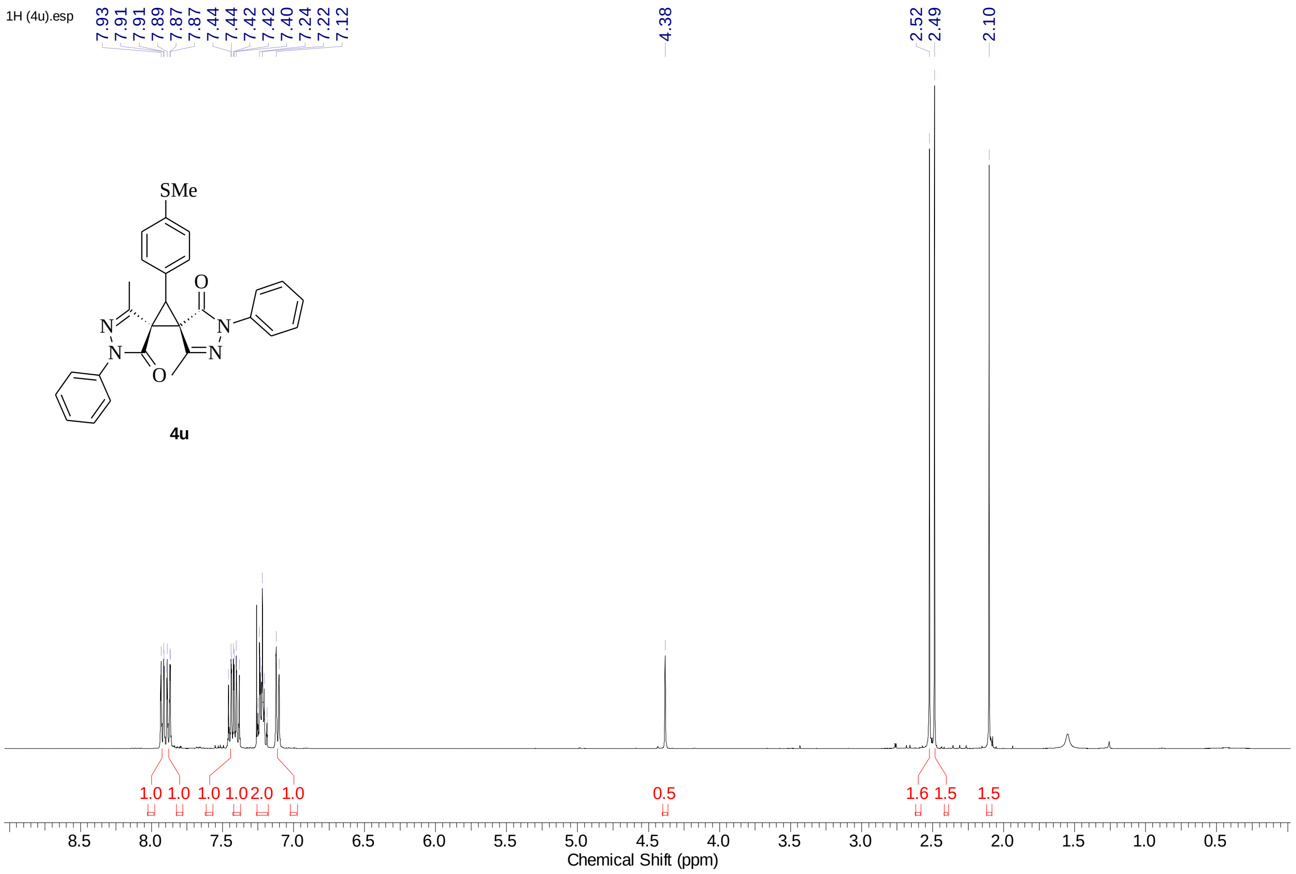

Figure S79. ¹H NMR spectrum of compound **4u**

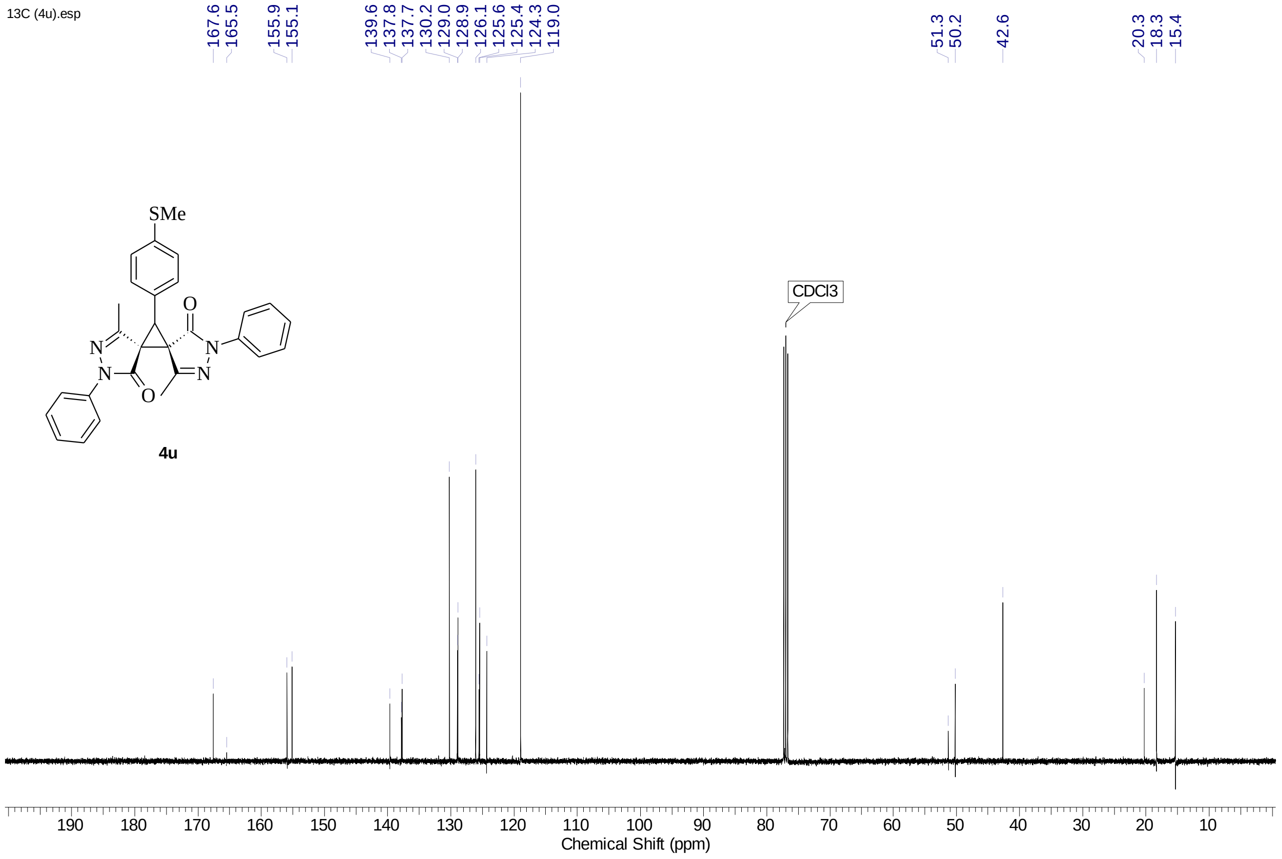

Figure S80. ¹³C NMR spectrum of compound **4u**

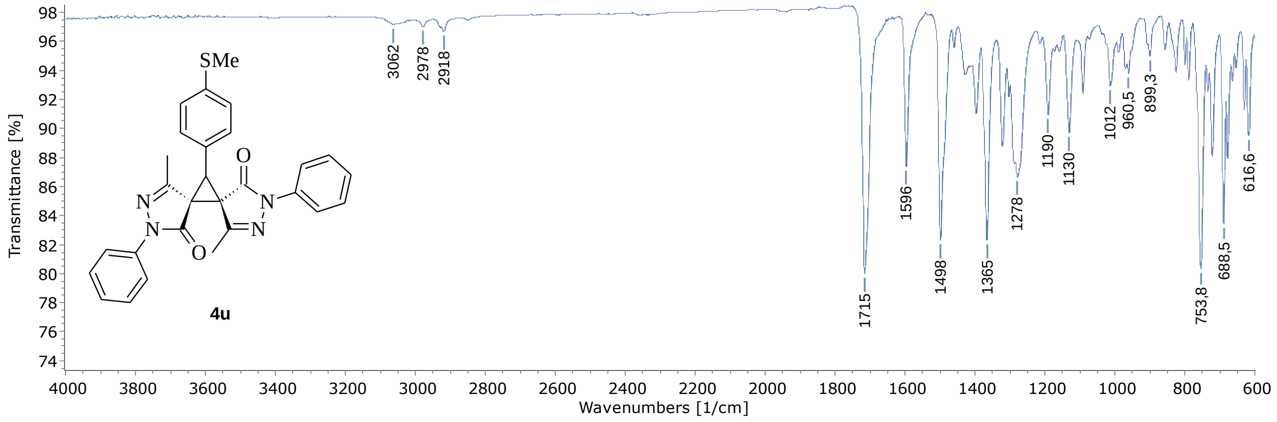

Figure S81. FTIR spectrum of compound **4u**

Acquisition Parameter

Ion Source Type	ESI	Ion Polarity	Positive	Alternating Ion Polarity	off
Mass Range Mode	UltraScan	Scan Begin	70 m/z	Scan End	600 m/z
Accumulation Time	200000 µs	RF Level	63 %	Trap Drive	54.1
SPS Target Mass	400 m/z	Averages	5 Spectra	n/a	n/a

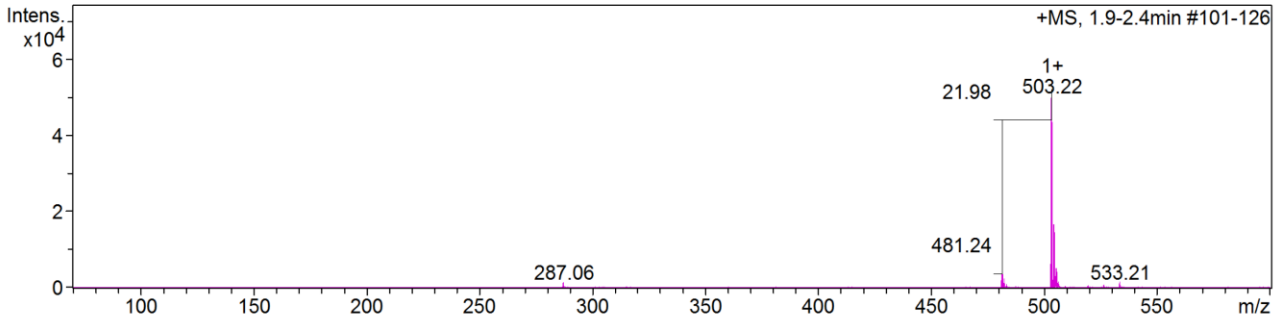

Component	Molecular Mass	Molecule		Absolute Abundance	Relative Abundance
A	503.20	503.63	[M + H]+	49718	100.00
B	481.23	481.71	[M + H]+	3790	7.62

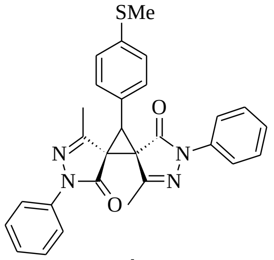

4u

Figure S82. ESI-MS spectrum of compound **4u**

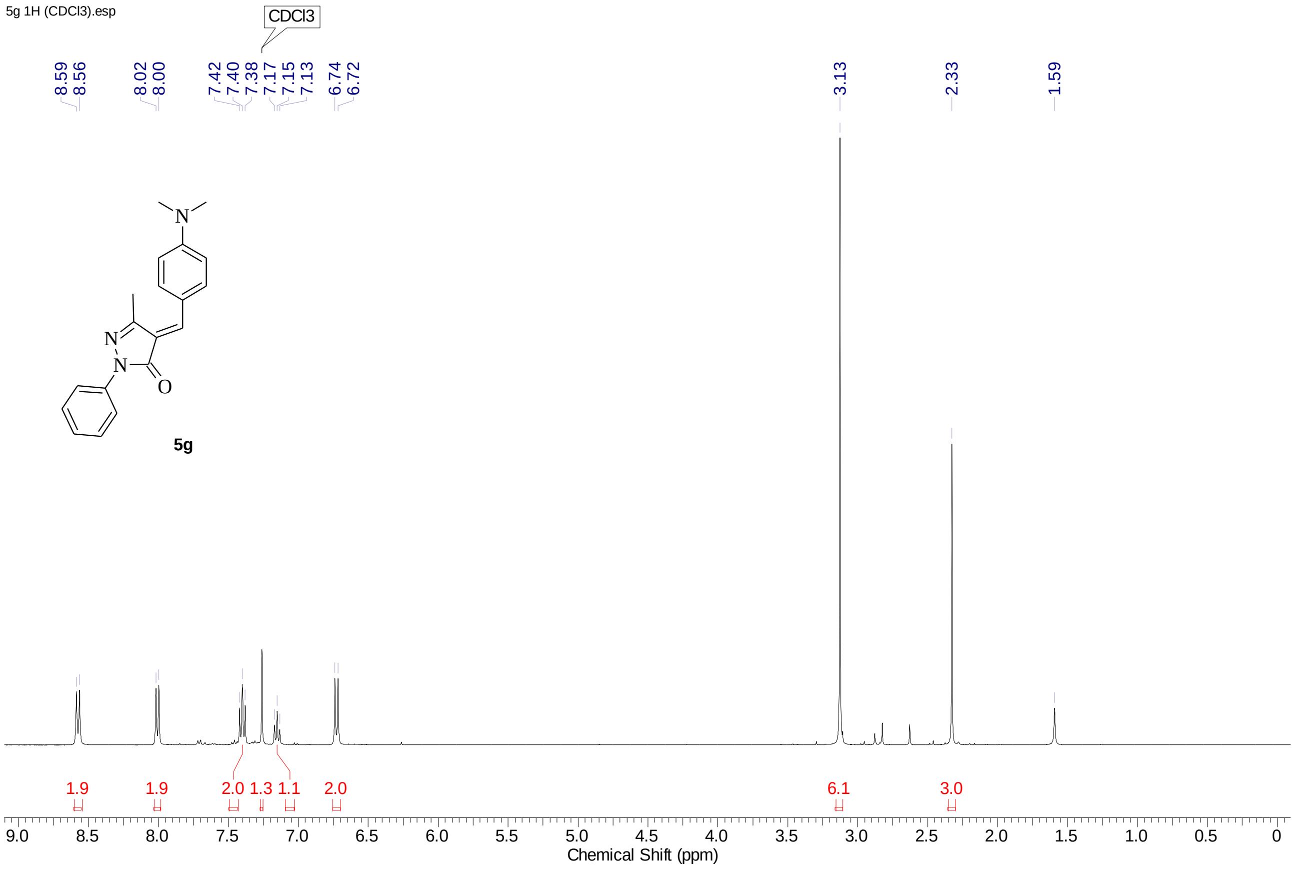

Figure S83. ^{1}H NMR spectrum of compound **5g**

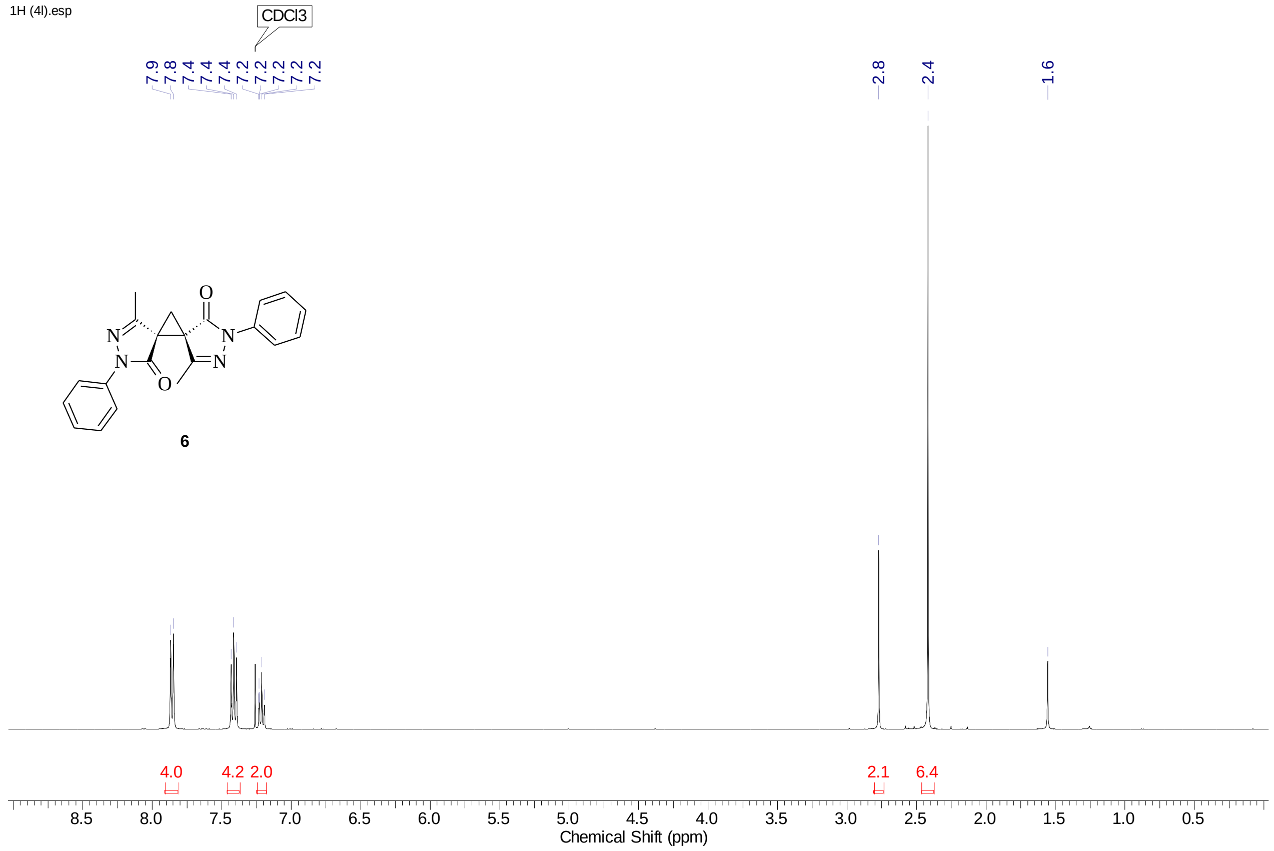

Figure S84. ^{1}H NMR spectrum of compound **6**

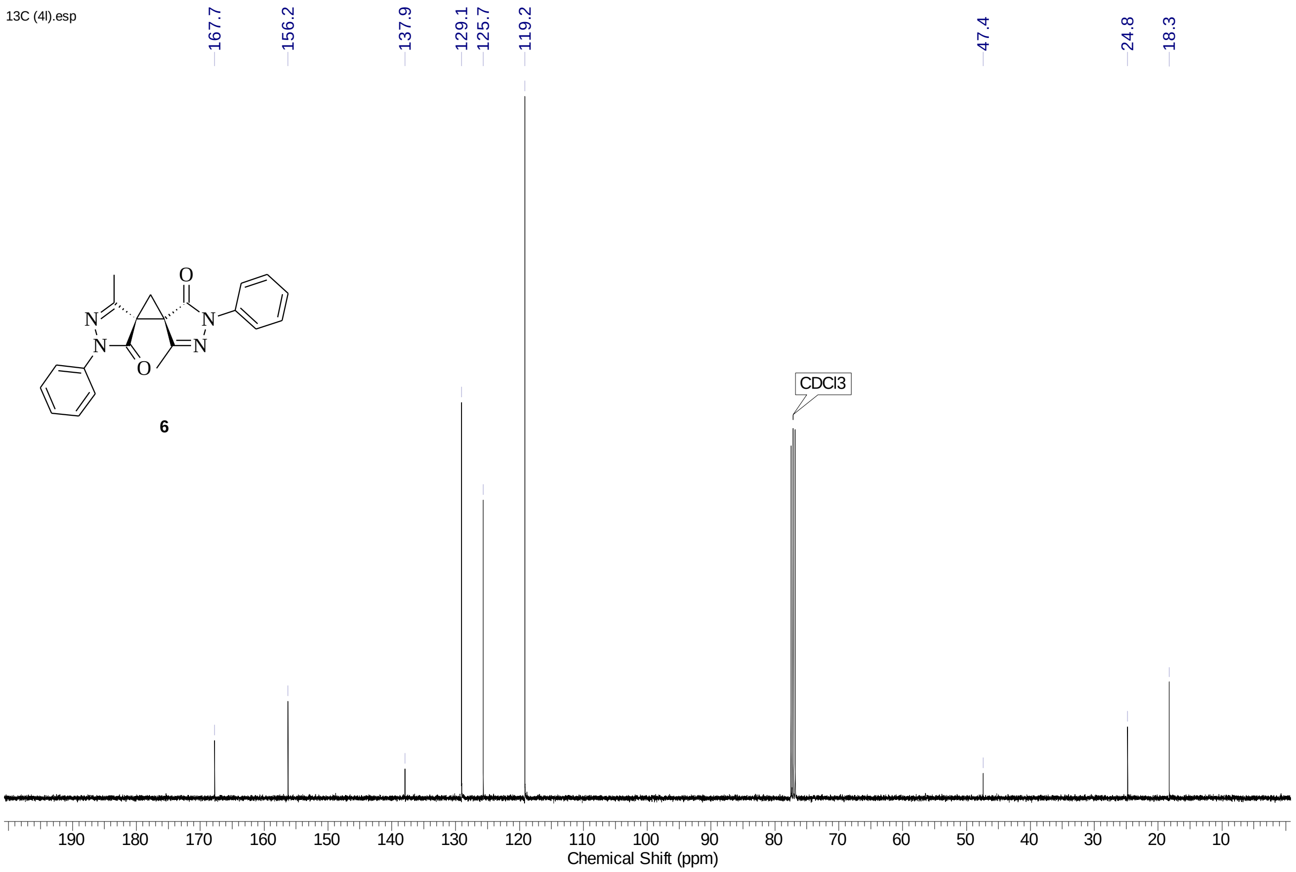

Figure S85. ^{13}C NMR spectrum of compound **6**

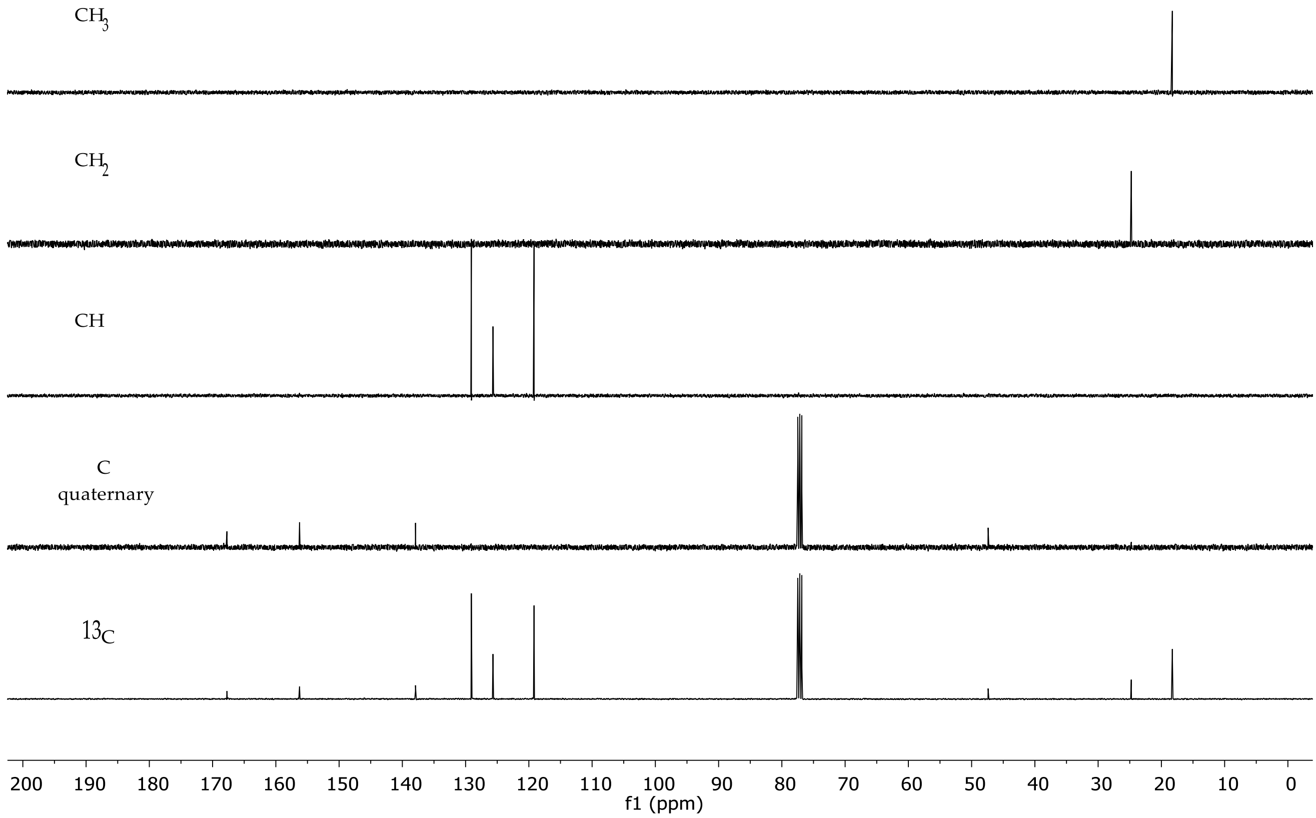

Figure S86. DEPT spectrum of compound **6**

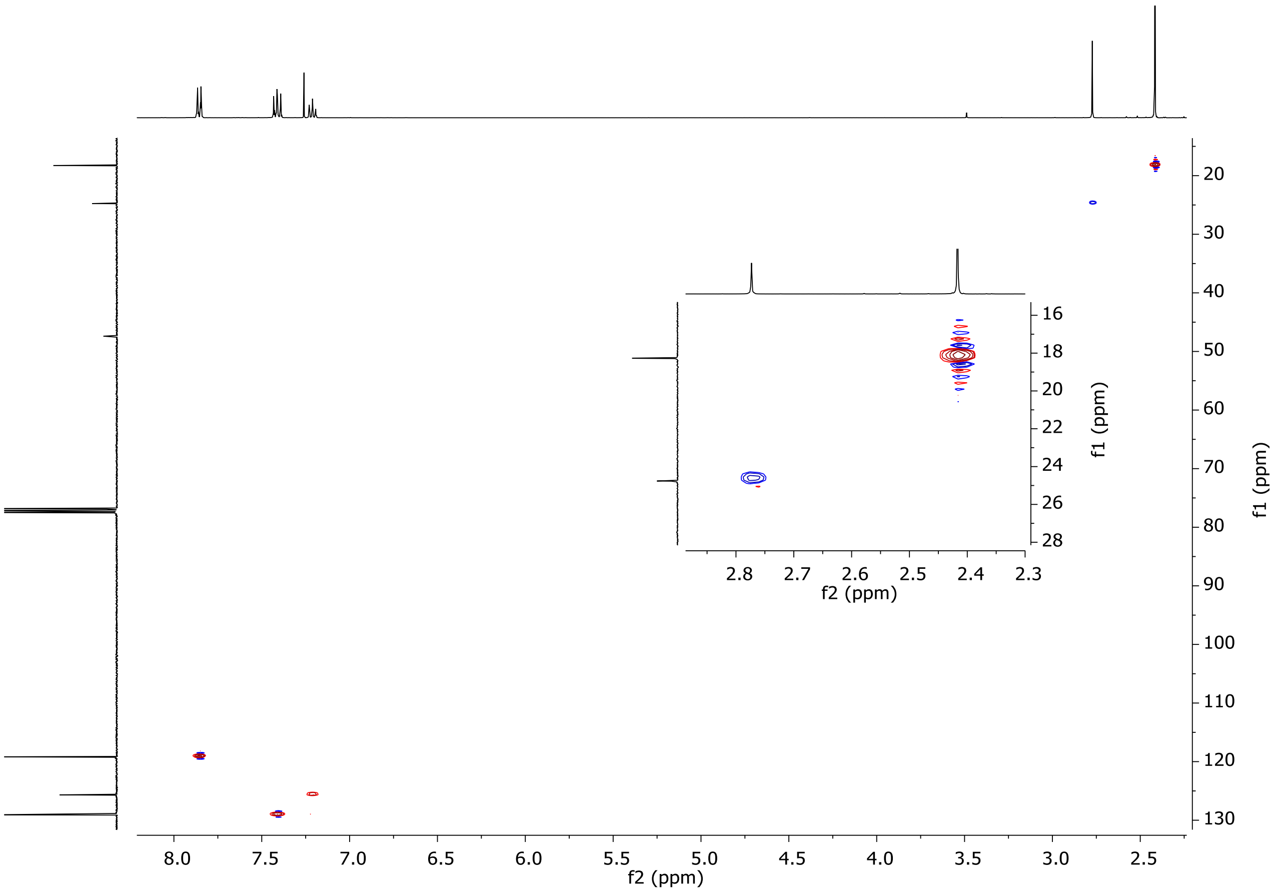

Figure S87. HSQC spectrum of compound **6**

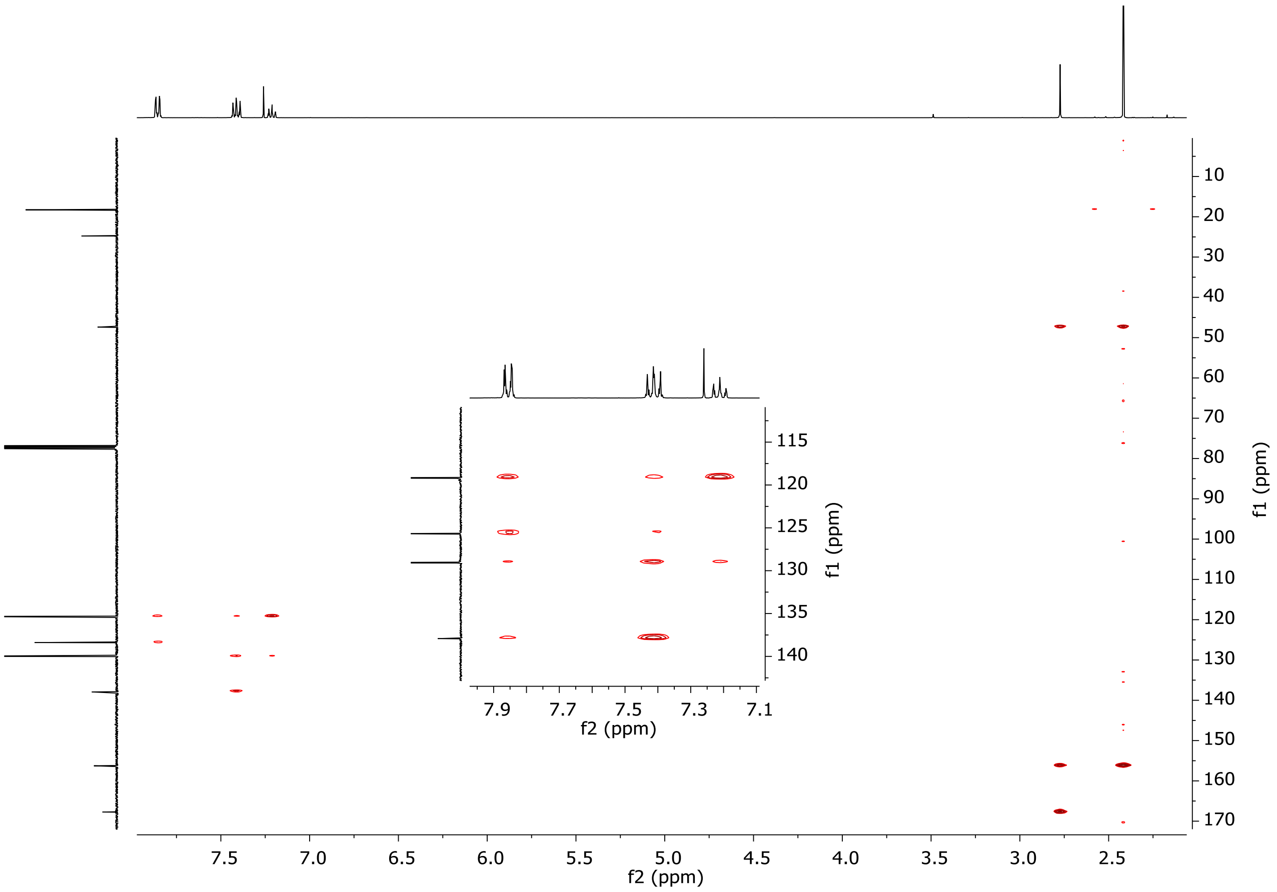

Figure S88. HMBC spectrum of compound **6**

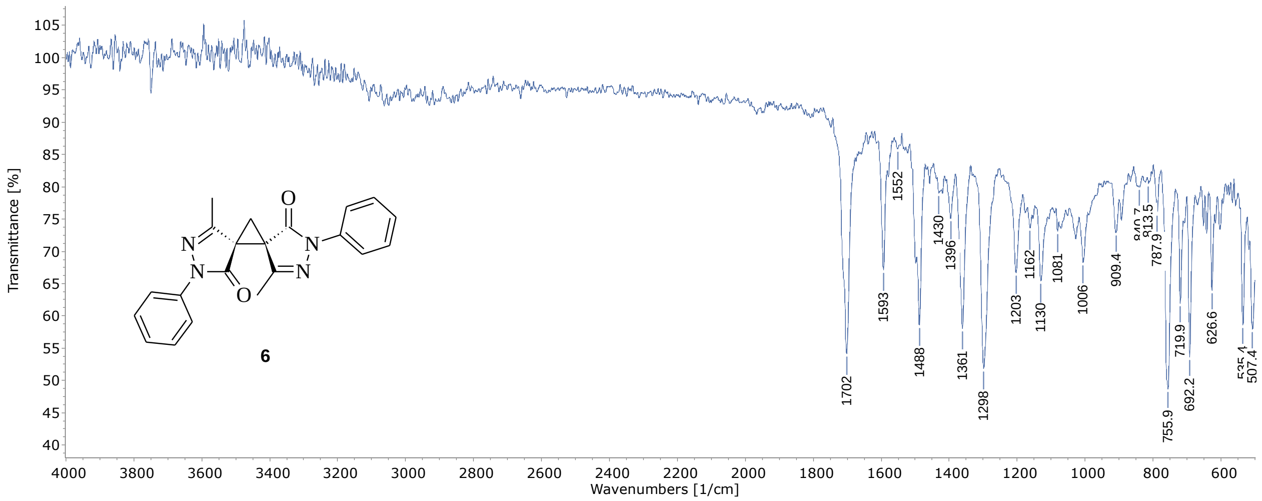

Figure S89. FTIR spectrum of compound **6**

Acquisition Parameter

Ion Source Type	ESI	Ion Polarity	Positive	Alternating Ion Polarity	off
Mass Range Mode	UltraScan	Scan Begin	70 m/z	Scan End	600 m/z
Accumulation Time	200000 µs	RF Level	63 %	Trap Drive	54.1
SPS Target Mass	400 m/z	Averages	5 Spectra	n/a	n/a

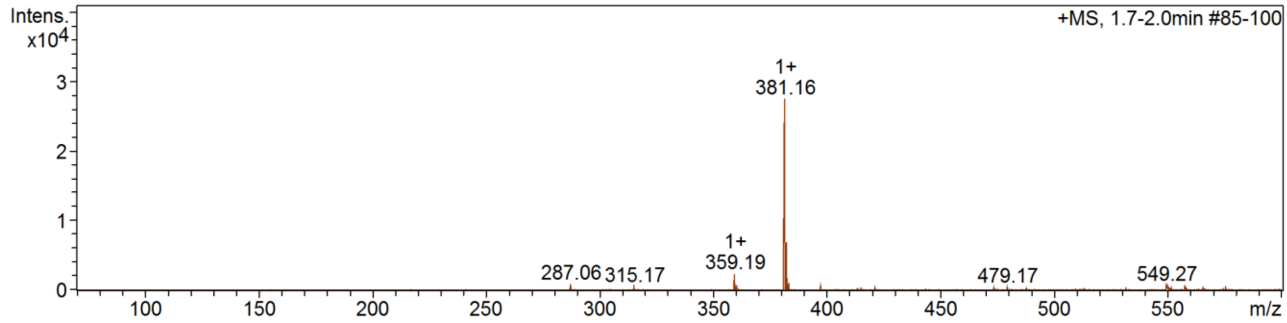

Component	Molecular Mass	Molecule		Absolute Abundance	Relative Abundance
A	381.15	381.42	[M + H]+	27417	100.00
B	359.19	359.55	[M + H]+	2350	8.57

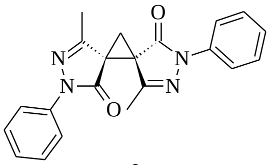

6

Figure S90. ESI-MS spectrum of compound **6**

Table S1. Percentage (%) of inhibition of five human cancer cell lines, and immortalized cell CHO K-1 exposed 48 h to 100 μM of each bis(spiropyrazolone)cyclopropanes **4.**

	RKO	PC-3	A-549	MCF-7	HeLa	CHO-K1
**4b**	21.74 ± 2.37	17.56 ± 2.83	NA	7.87 ± 2.92	NA	5.76 ± 3.08
**4c**	37.29 ± 1.22	11.03 ± 3.50	6.10 ± 0.86	16.69 ± 5.36	24.80 ± 1.74	2.62 ± 2.20
**4d**	68.88 ± 1.72	46.08 ± 1.91	13.64 ± 0.74	39.41 ± 2.29	48.56 ± 4.91	4.40 ± 0.72
**4e**	36.77 ± 1.55	17.93 ± 3.40	7.97 ± 1.46	4.47 ± 3.48	47.84 ± 2.45	NA
**4f**	28.74 ± 0.29	28.32 ± 2.56	10.36 ± 3.28	NA	NA	2.79 ± 1.49
**4k**	67.59 ± 0.77	46.72 ± 3.78	26.71 ± 3.03	43.03 ± 3.46	47.22 ± 1.36	NA
**4p**	18.59 ± 1.37	5.25 ± 2.60	9.08 ± 1.14	13.64 ± 2.55	5.98 ± 2.19	1.95 ± 1.42
**4q**	62.87 ± 1.53	50.51 ± 2.38	13.00 ± 1.99	32.48 ± 2.33	31.72 ± 1.52	2.29 ± 2.60
**4r**	64.71 ± 0.52	54.66 ± 1.95	28.65 ± 3.71	45.75 ± 2.11	50.16 ± 0.52	4.74 ± 3.04
**4s**	70.23 ± 1.19	49.74 ± 2.11	24.42 ± 1.36	9.91 ± 3.37	43.56 ± 1.75	NA
**4t**	47.57 ± 0.95	13.81 ± 2.87	3.43 ± 2.42	31.41 ± 3.65	5.06 ± 2.43	5.08 ± 2.84
**4u**	40.96 ± 1.49	38.68 ± 3.77	3.64 ± 1.81	19.51 ± 2.96	34.73 ± 2.29	5.76 ± 3.65
**Dox**	85.63 ± 1.06	87.98 ± 1.65	9.16 ± 2.16	46.23 ± 7.77	9.18 ± 9.55	2.62 ± 1.42

Data are given as mean and standard error (SEM) of at least three independent experiments; NA: No activity; Dox: Doxorubicin (1 μM).

Table S2. Inhibitory activity of bis(spiropyrazolone)cyclopropanes **4** against S. cerevisiae (W303 background).

	S. cerevisiae
	**W303**
**4b**	1377.67 ± 303.94
**4c**	NA
**4d**	NA
**4e**	NA
**4f**	NA
**4k**	NA
**4p**	NA
**4q**	1087.00 ± 38.50
**4r**	1270.00 ± 118.82
**4s**	789.03 ± 37.29
**4t**	NA
**4u**	NA

Table S3. DPPH scavenging activity of bis(spiropyrazolone)cyclopropanes **4.**

	**DPPH scavenging activity IC_{50} μM**
**4b**	> 300
**4c**	> 300
**4d**	> 300
**4e**	> 300
**4f**	> 300
**4k**	233.8
**4p**	> 300
**4q**	> 300
**4r**	> 300
**4s**	> 300
**4t**	> 300
**4u**	> 300
**Ascorbic acid**	14.0
**Ampho B**	ND

Statistical Analyses S1. Test for statistical significance were based on one-way ANOVA test applied to leishmanicidal and cytotoxicity activities of bis(spiropyrazolone)cyclopropanes **4** (Table 3).

```
>bartlett.test(log(IC50+1)~Drug)
Bartlett test of homogeneity of variances
data:  log(IC50 + 1) by Drug
Bartlett's K-squared = 18.182, df = 12, p-value = 0.1103

> ic50m=lm(log(IC50+1)~Drug)
> anova(ic50m)
Analysis of Variance Table
Response: log(IC50 + 1)
           Df   Sum Sq   Mean Sq   F value   Pr(>F)
Drug       12   3.01683 0.251403   46.477   6.327e-12 ***
Residuals  20   0.10818 0.005409
---
Signif. codes:  0 '***' 0.001 '**' 0.01 '*' 0.05 '.' 0.1 ' ' 1

Study: ic50m ~ "Drug"
HSD Test for log(IC50 + 1)
Mean Square Error:  0.005409134
```

Drug, means

	log.IC50...1.	std	r	Min	Max
4b	0.1463159	0.003543739	3	0.1431475	0.1501427
4c	0.1397771	0.034249039	3	0.1045402	0.1729444
4d	0.1377132	0.013678017	2	0.1280413	0.1473850
4e	0.9040759	0.103044742	3	0.7943491	0.9987920
4f	1.1732861	0.022310818	2	1.1575099	1.1890622
4k	0.2568298	0.074736324	3	0.1709235	0.3068963
4p	0.4487139	0.146863896	2	0.3448655	0.5525624
4q	0.2442968	0.033416047	3	0.2147887	0.2805819
4r	0.1716096	0.051448633	2	0.1352299	0.2079893
4s	0.1719498	0.057499706	2	0.1312914	0.2126082
4t	0.1948935	0.041308595	2	0.1656839	0.2241031
4u	0.3409402	0.174190218	2	0.2177691	0.4641113
Amphotericina	0.1573406	0.060319051	4	0.1024663	0.2312704

Alpha: 0.05 ; DF Error: 20

Critical Value of Studentized Range: 5.281538

Groups according to probability of means differences and alpha level(0.05)

Treatments with the same letter are not significantly different.

	log(IC50 + 1)	groups
4f	1.1732861	a
4e	0.9040759	b
4p	0.4487139	c
4u	0.3409402	cd
4k	0.2568298	cd
4q	0.2442968	cd
4t	0.1948935	cd
4s	0.1719498	d
4r	0.1716096	d
Amphotericina	0.1573406	d
4b	0.1463159	d
4c	0.1397771	d
4d	0.1377132	d

Original values:

	Group.1	x.1 (Mean)	x.2 (SD)
1	4b	0.157566667	0.004104063
2	4c	0.150466667	0.039335650
3	4d	0.147700000	0.015697771
4	4e	1.478333333	0.252224767

```
5           4f                  2.233000000   0.072124892
6           4k                  0.295200000   0.094711351
7           4p                  0.574750000   0.230446100
8           4q                  0.277200000   0.042880415
9           4r                  0.188000000   0.061094026
10           4s                 0.188600000   0.068306515
11           4t                 0.215700000   0.050204581
12           4u                 0.416950000   0.245578185
13 Amphotericina                0.172000000   0.071210158

> bartlett.test(log(CC50)~Drug)
            Bartlett test of homogeneity of variances
data:  log(CC50) by Drug
Bartlett's K-squared = 8.5922, df = 11, p-value = 0.6595
Analysis of Variance Table
Response:        log(CC50)
                  Df   Sum Sq   Mean Sq   F value    Pr(>F)
Drug              11   10.3815   0.94377    11.409   8.51e-08 ***
Residuals         29    2.3989   0.08272
---
Signif. codes:  0 '***' 0.001 '**' 0.01 '*' 0.05 '.' 0.1 ' ' 1

> HSD.test(CC50,"Drug",console=T)

Study: CC50m~ "Drug"

HSD Test for log(CC50)

Mean Square Error:  0.08271965

Drug,  means

     log.CC50.       std           r      Min       Max
4b  1.1719100   0.43666050   3  0.7323679  1.605631
4c  0.8128903   0.06772344   2  0.7650026  0.860778
4d  1.3342413   0.29842066   4  0.9783261  1.625705
4e  1.9649729   0.28245877   2  1.7652444  2.164701
4f  2.5482028   0.09670494   3  2.4379897  2.618855
4k  1.1645144   0.32332882   3  0.7911809  1.354029
4p  1.2466569   0.23752428   3  1.0466170  1.509175
4q  1.3739465   0.18446436   5  1.1568812  1.658228
4r  1.8612149   0.14882827   4  1.6420987  1.957698
```

4s	2.1056371	0.39517555	3	1.6787774	2.458734
4t	0.9820859	0.27456625	5	0.5300398	1.229641
4u	2.1711028	0.41188059	4	1.6152212	2.511224

Alpha: 0.05 ; DF Error: 29

Critical Value of Studentized Range: 5.014273

Groups according to probability of means differences and alpha level(0.05)

Treatments with the same letter are not significantly different.

	log(CC50)	groups
4f	2.5482028	a
4u	2.1711028	a
4s	2.1056371	ab
4e	1.9649729	abc
4r	1.8612149	abc
4q	1.3739465	bcd
4d	1.3342413	bcd
4p	1.2466569	cd
4b	1.1719100	cd
4k	1.1645144	cd
4t	0.9820859	d
4c	0.8128903	d

Original values:

	Group.1	x.1 (Mean)	x.2(sd)
1	4b	3.4360000	1.4597058
2	4c	2.2570000	0.1527351
3	4d	3.9225000	1.1214757
4	4e	7.2775000	2.0286894
5	4f	12.8233333	1.2077389
6	4k	3.3100000	0.9561553
7	4p	3.5463333	0.8714978
8	4q	4.0066000	0.7724440
9	4r	6.4827500	0.8992754
10	4s	8.6300000	3.1707698
11	4t	2.7450000	0.6646112
12	4u	9.2952500	3.3663162

Statistical Analyses S1. Kruskal Wallis test´s applied to comet assay of Leishmania mexicana cells exposed to bis(spiropyrazolone)cyclopropanes **4** (Figure 2).

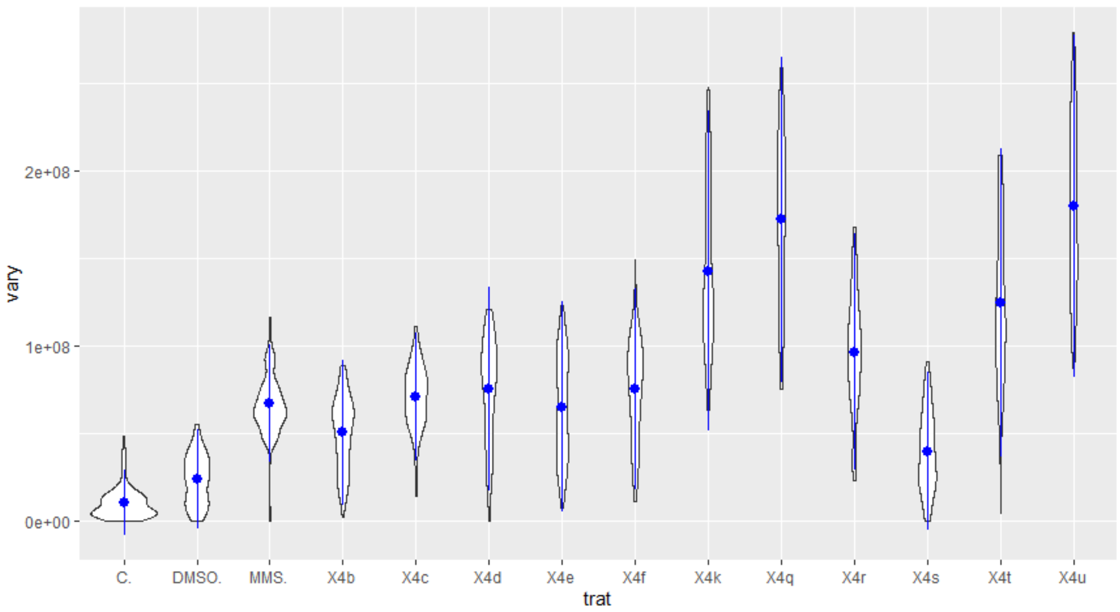

Study: Trat
Kruskal-Wallis test's
Ties or no Ties

Critical Value: 1004.543
Degrees of freedom: 13
Pvalue Chisq : 0

trat, means of the ranks

	vary	r
C.	97.280	100
DMSO.	207.115	100
MMS.	606.840	100
X4b	449.315	100
X4c	648.065	100
X4d	694.660	100
X4e	595.490	100
X4f	691.135	100
X4k	1111.660	100
X4q	1208.625	80
X4r	861.180	100
X4s	352.245	100
X4t	1016.210	100
X4u	1230.805	100

Post Hoc Analysis

P value adjustment method: bonferroni
t-Student: 3.463614
Alpha : 0.05
Groups according to probability of treatment differences and alpha level.

Treatments with the same letter are not significantly different.

	vary	groups
X4u	1230.805	a
X4q	1208.625	ab
X4k	1111.660	bc
X4t	1016.210	c
X4r	861.180	d
X4d	694.660	e
X4f	691.135	e
X4c	648.065	e
MMS.	606.840	e
X4e	595.490	e
X4b	449.315	f
X4s	352.245	f
DMSO.	207.115	g
C.	97.280	h

$statistics

Chisq	Df	p.chisq
1004.543	13	0

$parameters

test	p.ajusted	name.t	ntr	alpha
Kruskal-Wallis	bonferroni	trat	14	0.05

$means

	vary	rank	std	r	Min	Max	Q25	Q50	Q75
C.	10290000	97.280	9224796	100	0.0e+00	4.80e+07	4000000	8000000	14000000
DMSO.	23610000	207.115	14071890	100	0.0e+00	5.50e+07	11000000	25000000	35000000
MMS.	67050000	606.840	16924894	100	0.0e+00	1.16e+08	58000000	65000000	74250000
X4b	50400000	449.315	20682301	100	2.0e+06	8.90e+07	32750000	55000000	64000000
X4c	70710000	648.065	18193847	100	1.4e+07	1.11e+08	58750000	72500000	83250000
X4d	75120000	694.660	29056295	100	0.0e+00	1.21e+08	52000000	83500000	97250000
X4e	65120000	595.490	29979549	100	7.0e+06	1.23e+08	39750000	64500000	90250000
X4f	75100000	691.135	28784641	100	1.1e+07	1.49e+08	55000000	80500000	99000000
X4k	142780000	1111.660	45723716	100	6.3e+07	2.48e+08	110000000	128000000	182500000
X4q	172025000	1208.625	46560965	80	7.5e+07	2.59e+08	129750000	177500000	205750000
X4r	96310000	861.180	33823277	100	2.3e+07	1.68e+08	76500000	98500000	117500000
X4s	39720000	352.245	22688528	100	0.0e+00	9.10e+07	23000000	39500000	55250000
X4t	124410000	1016.210	44002317	100	4.0e+06	2.09e+08	95250000	116000000	157000000
X4u	179560000	1230.805	48875087	100	8.7e+07	2.79e+08	142000000	177500000	223000000

$comparison

	Difference	pvalue	Signif.	LCL	UCL
C. - DMSO.	-109.835	0.0187	*	-212.036162	-7.633838
C. - MMS.	-509.560	0.0000	***	-611.761162	-407.358838
C. - X4b	-352.035	0.0000	***	-454.236162	-249.833838
C. - X4c	-550.785	0.0000	***	-652.986162	-448.583838
C. - X4d	-597.380	0.0000	***	-699.581162	-495.178838
C. - X4e	-498.210	0.0000	***	-600.411162	-396.008838
C. - X4f	-593.855	0.0000	***	-696.056162	-491.653838
C. - X4k	-1014.380	0.0000	***	-1116.581162	-912.178838
C. - X4q	-1111.345	0.0000	***	-1219.745702	-1002.944298
C. - X4r	-763.900	0.0000	***	-866.101162	-661.698838
C. - X4s	-254.965	0.0000	***	-357.166162	-152.763838
C. - X4t	-918.930	0.0000	***	-1021.131162	-816.728838
C. - X4u	-1133.525	0.0000	***	-1235.726162	-1031.323838
DMSO. - MMS.	-399.725	0.0000	***	-501.926162	-297.523838
DMSO. - X4b	-242.200	0.0000	***	-344.401162	-139.998838
DMSO. - X4c	-440.950	0.0000	***	-543.151162	-338.748838
DMSO. - X4d	-487.545	0.0000	***	-589.746162	-385.343838
DMSO. - X4e	-388.375	0.0000	***	-490.576162	-286.173838
DMSO. - X4f	-484.020	0.0000	***	-586.221162	-381.818838
DMSO. - X4k	-904.545	0.0000	***	-1006.746162	-802.343838
DMSO. - X4q	-1001.510	0.0000	***	-1109.910702	-893.109298
DMSO. - X4r	-654.065	0.0000	***	-756.266162	-551.863838
DMSO. - X4s	-145.130	0.0001	***	-247.331162	-42.928838
DMSO. - X4t	-809.095	0.0000	***	-911.296162	-706.893838
DMSO. - X4u	-1023.690	0.0000	***	-1125.891162	-921.488838
MMS. - X4b	157.525	0.0000	***	55.323838	259.726162
MMS. - X4c	-41.225	1.0000		-143.426162	60.976162
MMS. - X4d	-87.820	0.2702		-190.021162	14.381162
MMS. - X4e	11.350	1.0000		-90.851162	113.551162
MMS. - X4f	-84.295	0.3954		-186.496162	17.906162
MMS. - X4k	-504.820	0.0000	***	-607.021162	-402.618838
MMS. - X4q	-601.785	0.0000	***	-710.185702	-493.384298
MMS. - X4r	-254.340	0.0000	***	-356.541162	-152.138838
MMS. - X4s	254.595	0.0000	***	152.393838	356.796162
MMS. - X4t	-409.370	0.0000	***	-511.571162	-307.168838
MMS. - X4u	-623.965	0.0000	***	-726.166162	-521.763838
X4b - X4c	-198.750	0.0000	***	-300.951162	-96.548838
X4b - X4d	-245.345	0.0000	***	-347.546162	-143.143838
X4b - X4e	-146.175	0.0001	***	-248.376162	-43.973838
X4b - X4f	-241.820	0.0000	***	-344.021162	-139.618838
X4b - X4k	-662.345	0.0000	***	-764.546162	-560.143838
X4b - X4q	-759.310	0.0000	***	-867.710702	-650.909298
X4b - X4r	-411.865	0.0000	***	-514.066162	-309.663838
X4b - X4s	97.070	0.0936	.	-5.131162	199.271162
X4b - X4t	-566.895	0.0000	***	-669.096162	-464.693838
X4b - X4u	-781.490	0.0000	***	-883.691162	-679.288838
X4c - X4d	-46.595	1.0000		-148.796162	55.606162
X4c - X4e	52.575	1.0000		-49.626162	154.776162
X4c - X4f	-43.070	1.0000		-145.271162	59.131162
X4c - X4k	-463.595	0.0000	***	-565.796162	-361.393838

X4c - X4q	-560.560	0.0000	***	-668.960702	-452.159298
X4c - X4r	-213.115	0.0000	***	-315.316162	-110.913838
X4c - X4s	295.820	0.0000	***	193.618838	398.021162
X4c - X4t	-368.145	0.0000	***	-470.346162	-265.943838
X4c - X4u	-582.740	0.0000	***	-684.941162	-480.538838
X4d - X4e	99.170	0.0727	.	-3.031162	201.371162
X4d - X4f	3.525	1.0000		-98.676162	105.726162
X4d - X4k	-417.000	0.0000	***	-519.201162	-314.798838
X4d - X4q	-513.965	0.0000	***	-622.365702	-405.564298
X4d - X4r	-166.520	0.0000	***	-268.721162	-64.318838
X4d - X4s	342.415	0.0000	***	240.213838	444.616162
X4d - X4t	-321.550	0.0000	***	-423.751162	-219.348838
X4d - X4u	-536.145	0.0000	***	-638.346162	-433.943838
X4e - X4f	-95.645	0.1108		-197.846162	6.556162
X4e - X4k	-516.170	0.0000	***	-618.371162	-413.968838
X4e - X4q	-613.135	0.0000	***	-721.535702	-504.734298
X4e - X4r	-265.690	0.0000	***	-367.891162	-163.488838
X4e - X4s	243.245	0.0000	***	141.043838	345.446162
X4e - X4t	-420.720	0.0000	***	-522.921162	-318.518838
X4e - X4u	-635.315	0.0000	***	-737.516162	-533.113838
X4f - X4k	-420.525	0.0000	***	-522.726162	-318.323838
X4f - X4q	-517.490	0.0000	***	-625.890702	-409.089298
X4f - X4r	-170.045	0.0000	***	-272.246162	-67.843838
X4f - X4s	338.890	0.0000	***	236.688838	441.091162
X4f - X4t	-325.075	0.0000	***	-427.276162	-222.873838
X4f - X4u	-539.670	0.0000	***	-641.871162	-437.468838
X4k - X4q	-96.965	0.1808		-205.365702	11.435702
X4k - X4r	250.480	0.0000	***	148.278838	352.681162
X4k - X4s	759.415	0.0000	***	657.213838	861.616162
X4k - X4t	95.450	0.1134		-6.751162	197.651162
X4k - X4u	-119.145	0.0052	**	-221.346162	-16.943838
X4q - X4r	347.445	0.0000	***	239.044298	455.845702
X4q - X4s	856.380	0.0000	***	747.979298	964.780702
X4q - X4t	192.415	0.0000	***	84.014298	300.815702
X4q - X4u	-22.180	1.0000		-130.580702	86.220702
X4r - X4s	508.935	0.0000	***	406.733838	611.136162
X4r - X4t	-155.030	0.0000	***	-257.231162	-52.828838
X4r - X4u	-369.625	0.0000	***	-471.826162	-267.423838
X4s - X4t	-663.965	0.0000	***	-766.166162	-561.763838
X4s - X4u	-878.560	0.0000	***	-980.761162	-776.358838
X4t - X4u	-214.595	0.0000	***	-316.796162	-112.393838

Statistical Analyses S2. Kruskal Wallis test´s applied to comet assay of CHO cells exposed to bis(spiropyrazolone)cyclopropanes **4** (Figure 3).

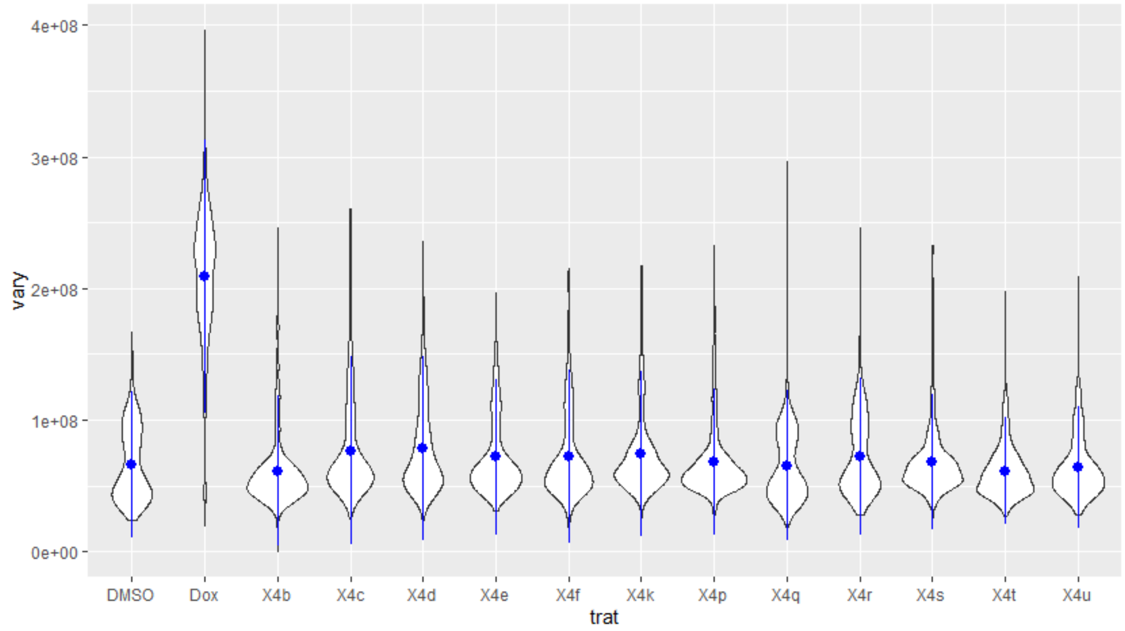

$statistics

Chisq	Df	p.chisq	t.value	MSD
1708.307	13	0	1.960247	245.1376

$parameters

test	p.ajusted	name.t	ntr	alpha
Kruskal-Wallis	none	trat	14	0.05

$means

	vary	rank	std	r	Min	Max	Q25	Q50	Q75
DMSO	66045840	3568.301	27896533	600	23429540.0	166553500	42784380	57045840	89134120
Dox	209447368	7865.864	51979579	600	19864180.0	396264900	183361600	217996600	240916800
X4b	61401527	3115.134	28639223	600	509337.8	246519500	45331070	53989810	65195240
X4c	76526316	4376.037	35814130	600	24957560.0	260781000	52843805	62648560	92954160
X4d	78343803	4521.320	34892342	600	23429540.0	235314100	52461800	65704580	95755520
X4e	71876061	4164.706	29613974	600	31069610.0	197113800	51443120	61120540	82640065
X4f	72036501	4055.789	32998506	600	18845500.0	215449900	49915110	61629880	83022070
X4k	74173175	4427.186	31543285	600	25976230.0	217487300	55008490	63667230	80093375
X4p	68013582	3946.407	27613069	600	27504240.0	232767400	51825125	60101870	71816640
X4q	65551784	3537.702	28621584	600	18336160.0	296944000	43293720	56791170	86714765
X4r	72132428	4106.395	29902529	600	27504240.0	246010200	48896430	61629880	94736840
X4s	68389643	4118.727	25773413	600	25976230.0	232258100	52971140	62648560	73344650
X4t	61194397	3386.662	20212790	600	26485570.0	198132400	46859090	57045840	69779290
X4u	64116299	3616.770	23301875	600	28013580.0	209337900	47877760	58573850	72325970

$comparison
NULL

$groups

	vary	groups
Dox	7865.864	a
X4d	4521.320	b

```
X4k  4427.186      b
X4c  4376.037     bc
X4e  4164.706     cd
X4s  4118.727      d
X4r  4106.395      d
X4f  4055.789      d
X4p  3946.407      d
X4u  3616.770      e
DMSO 3568.301      e
X4q  3537.702      e
X4t  3386.662      e
X4b  3115.134      f

$statistics
    Chisq Df p.chisq  t.value      MSD
  1708.307 13       0 3.456746 432.2815

$parameters
          test  p.ajusted name.t ntr alpha
  Kruskal-Wallis bonferroni   trat  14  0.05

$means
        vary     rank      std   r       Min       Max       Q25       Q50       Q75
DMSO  66045840 3568.301 27896533 600 23429540.0 166553500  42784380  57045840  89134120
Dox  209447368 7865.864 51979579 600 19864180.0 396264900 183361600 217996600 240916800
X4b   61401527 3115.134 28639223 600   509337.8 246519500  45331070  53989810  65195240
X4c   76526316 4376.037 35814130 600 24957560.0 260781000  52843805  62648560  92954160
X4d   78343803 4521.320 34892342 600 23429540.0 235314100  52461800  65704580  95755520
X4e   71876061 4164.706 29613974 600 31069610.0 197113800  51443120  61120540  82640065
X4f   72036501 4055.789 32998506 600 18845500.0 215449900  49915110  61629880  83022070
X4k   74173175 4427.186 31543285 600 25976230.0 217487300  55008490  63667230  80093375
X4p   68013582 3946.407 27613069 600 27504240.0 232767400  51825125  60101870  71816640
X4q   65551784 3537.702 28621584 600 18336160.0 296944000  43293720  56791170  86714765
X4r   72132428 4106.395 29902529 600 27504240.0 246010200  48896430  61629880  94736840
X4s   68389643 4118.727 25773413 600 25976230.0 232258100  52971140  62648560  73344650
X4t   61194397 3386.662 20212790 600 26485570.0 198132400  46859090  57045840  69779290
X4u   64116299 3616.770 23301875 600 28013580.0 209337900  47877760  58573850  72325970

$comparison
          Difference pvalue Signif.          LCL          UCL
DMSO - Dox -4297.56333 0.0000     *** -4729.844865 -3865.281801
DMSO - X4b   453.16667 0.0266       *    20.885135   885.448199
DMSO - X4c  -807.73583 0.0000     *** -1240.017365  -375.454301
DMSO - X4d  -953.01917 0.0000     *** -1385.300699  -520.737635
DMSO - X4e  -596.40500 0.0002     *** -1028.686532  -164.123468
DMSO - X4f  -487.48833 0.0089      **  -919.769865   -55.206801
DMSO - X4k  -858.88500 0.0000     *** -1291.166532  -426.603468
DMSO - X4p  -378.10583 0.2280          -810.387365    54.175699
DMSO - X4q    30.59917 1.0000          -401.682365   462.880699
DMSO - X4r  -538.09417 0.0016      **  -970.375699  -105.812635
DMSO - X4s  -550.42667 0.0010     ***  -982.708199  -118.145135
DMSO - X4t   181.63833 1.0000          -250.643199   613.919865
```

DMSO - X4u	-48.46917	1.0000		-480.750699	383.812365
Dox - X4b	4750.73000	0.0000	***	4318.448468	5183.011532
Dox - X4c	3489.82750	0.0000	***	3057.545968	3922.109032
Dox - X4d	3344.54417	0.0000	***	2912.262635	3776.825699
Dox - X4e	3701.15833	0.0000	***	3268.876801	4133.439865
Dox - X4f	3810.07500	0.0000	***	3377.793468	4242.356532
Dox - X4k	3438.67833	0.0000	***	3006.396801	3870.959865
Dox - X4p	3919.45750	0.0000	***	3487.175968	4351.739032
Dox - X4q	4328.16250	0.0000	***	3895.880968	4760.444032
Dox - X4r	3759.46917	0.0000	***	3327.187635	4191.750699
Dox - X4s	3747.13667	0.0000	***	3314.855135	4179.418199
Dox - X4t	4479.20167	0.0000	***	4046.920135	4911.483199
Dox - X4u	4249.09417	0.0000	***	3816.812635	4681.375699
X4b - X4c	-1260.90250	0.0000	***	-1693.184032	-828.620968
X4b - X4d	-1406.18583	0.0000	***	-1838.467365	-973.904301
X4b - X4e	-1049.57167	0.0000	***	-1481.853199	-617.290135
X4b - X4f	-940.65500	0.0000	***	-1372.936532	-508.373468
X4b - X4k	-1312.05167	0.0000	***	-1744.333199	-879.770135
X4b - X4p	-831.27250	0.0000	***	-1263.554032	-398.990968
X4b - X4q	-422.56750	0.0665	.	-854.849032	9.714032
X4b - X4r	-991.26083	0.0000	***	-1423.542365	-558.979301
X4b - X4s	-1003.59333	0.0000	***	-1435.874865	-571.311801
X4b - X4t	-271.52833	1.0000		-703.809865	160.753199
X4b - X4u	-501.63583	0.0055	**	-933.917365	-69.354301
X4c - X4d	-145.28333	1.0000		-577.564865	286.998199
X4c - X4e	211.33083	1.0000		-220.950699	643.612365
X4c - X4f	320.24750	0.9517		-112.034032	752.529032
X4c - X4k	-51.14917	1.0000		-483.430699	381.132365
X4c - X4p	429.63000	0.0541	.	-2.651532	861.911532
X4c - X4q	838.33500	0.0000	***	406.053468	1270.616532
X4c - X4r	269.64167	1.0000		-162.639865	701.923199
X4c - X4s	257.30917	1.0000		-174.972365	689.590699
X4c - X4t	989.37417	0.0000	***	557.092635	1421.655699
X4c - X4u	759.26667	0.0000	***	326.985135	1191.548199
X4d - X4e	356.61417	0.3967		-75.667365	788.895699
X4d - X4f	465.53083	0.0181	*	33.249301	897.812365
X4d - X4k	94.13417	1.0000		-338.147365	526.415699
X4d - X4p	574.91333	0.0004	***	142.631801	1007.194865
X4d - X4q	983.61833	0.0000	***	551.336801	1415.899865
X4d - X4r	414.92500	0.0829	.	-17.356532	847.206532
X4d - X4s	402.59250	0.1174		-29.689032	834.874032
X4d - X4t	1134.65750	0.0000	***	702.375968	1566.939032
X4d - X4u	904.55000	0.0000	***	472.268468	1336.831532
X4e - X4f	108.91667	1.0000		-323.364865	541.198199
X4e - X4k	-262.48000	1.0000		-694.761532	169.801532
X4e - X4p	218.29917	1.0000		-213.982365	650.580699
X4e - X4q	627.00417	0.0000	***	194.722635	1059.285699
X4e - X4r	58.31083	1.0000		-373.970699	490.592365
X4e - X4s	45.97833	1.0000		-386.303199	478.259865
X4e - X4t	778.04333	0.0000	***	345.761801	1210.324865
X4e - X4u	547.93583	0.0011	**	115.654301	980.217365
X4f - X4k	-371.39667	0.2719		-803.678199	60.884865
X4f - X4p	109.38250	1.0000		-322.899032	541.664032

```
X4f - X4q    518.08750 0.0032   **    85.805968   950.369032
X4f - X4r    -50.60583 1.0000        -482.887365   381.675699
X4f - X4s    -62.93833 1.0000        -495.219865   369.343199
X4f - X4t    669.12667 0.0000   ***  236.845135  1101.408199
X4f - X4u    439.01917 0.0409   *      6.737635   871.300699
X4k - X4p    480.77917 0.0111   *     48.497635   913.060699
X4k - X4q    889.48417 0.0000   ***  457.202635  1321.765699
X4k - X4r    320.79083 0.9399        -111.490699   753.072365
X4k - X4s    308.45833 1.0000        -123.823199   740.739865
X4k - X4t   1040.52333 0.0000   ***  608.241801  1472.804865
X4k - X4u    810.41583 0.0000   ***  378.134301  1242.697365
X4p - X4q    408.70500 0.0989   .    -23.576532   840.986532
X4p - X4r   -159.98833 1.0000        -592.269865   272.293199
X4p - X4s   -172.32083 1.0000        -604.602365   259.960699
X4p - X4t    559.74417 0.0007   ***  127.462635   992.025699
X4p - X4u    329.63667 0.7649        -102.644865   761.918199
X4q - X4r   -568.69333 0.0005   *** -1000.974865  -136.411801
X4q - X4s   -581.02583 0.0003   *** -1013.307365  -148.744301
X4q - X4t    151.03917 1.0000        -281.242365   583.320699
X4q - X4u    -79.06833 1.0000        -511.349865   353.213199
X4r - X4s    -12.33250 1.0000        -444.614032   419.949032
X4r - X4t    719.73250 0.0000   ***  287.450968  1152.014032
X4r - X4u    489.62500 0.0083   **    57.343468   921.906532
X4s - X4t    732.06500 0.0000   ***  299.783468  1164.346532
X4s - X4u    501.95750 0.0055   **    69.675968   934.239032
X4t - X4u   -230.10750 1.0000        -662.389032   202.174032

$groups
NULL
```
